# Effects on Dopaminergic Neurons Are Secondary in COX-Deficient Locomotor Dysfunction in *Drosophila*

**DOI:** 10.1016/j.isci.2020.101362

**Published:** 2020-07-12

**Authors:** Cagri Yalgin, Bohdana Rovenko, Ana Andjelković, Margot Neefjes, Burak Oymak, Eric Dufour, Ville Hietakangas, Howard T. Jacobs

**Affiliations:** 1 Faculty of Medicine and Health Technology, FI-33014 Tampere University, Finland; 2 Institute of Biotechnology, FI-00014 University of Helsinki, Finland; 3 Faculty of Biological and Environmental Sciences, FI-00014 University of Helsinki, Finland

**Keywords:** Cell Biology, Molecular Biology, Neuroscience

## Abstract

Dopaminergic (DA) neurons have been implicated as key targets in neurological disorders, notably those involving locomotor impairment, and are considered to be highly vulnerable to mitochondrial dysfunction, a common feature of such diseases. Here we investigated a *Drosophila* model of locomotor disorders in which functional impairment is brought about by pan-neuronal RNAi knockdown of subunit COX7A of cytochrome oxidase (COX). Despite minimal neuronal loss by apoptosis, the expression and activity of tyrosine hydroxylase was decreased by half. Surprisingly, COX7A knockdown specifically targeted to DA neurons did not produce locomotor defect. Instead, using various drivers, we found that COX7A knockdown in specific groups of cholinergic and glutamatergic neurons underlay the phenotype. Based on our main finding, the vulnerability of DA neurons to mitochondrial dysfunction as a cause of impaired locomotion in other organisms, including mammals, warrants detailed investigation.

## Highlights

• Neuronal knockdown of a COX subunit produces locomotor defect in flies
• Specific subsets of cholinergic and glutamatergic (not DA) neurons are vulnerable
• Decreased tyrosine hydroxylase is a secondary or side effect
• Extensive neuronal cell death is not involved in the phenotype

## Graphical Abstract

**Figure:**
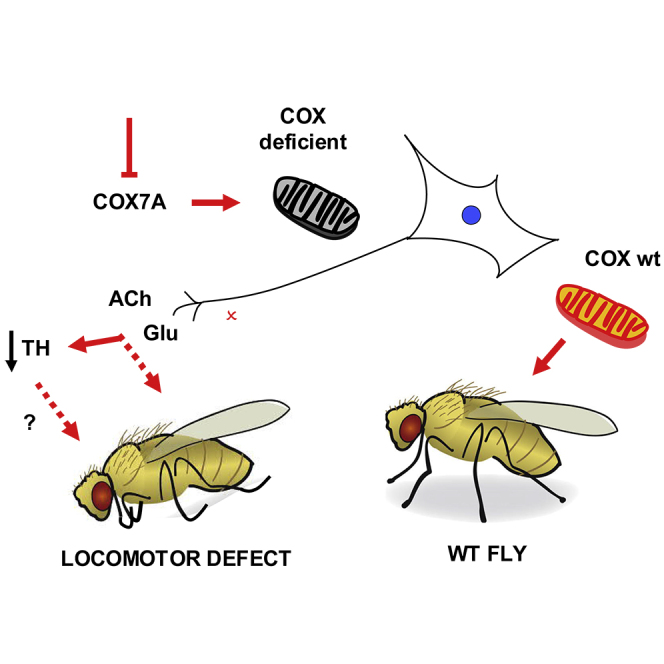

## Introduction

Mitochondrial stress, whether resulting from impaired respiration, oxidative damage, or defective protein quality control, has been proposed as a major underlying process in neurological diseases, notably those where locomotion is impaired, such as Parkinson's disease (PD; recently reviewed by Ge et al., 2020). The main pathological target in such diseases is dopaminergic (DA) neurons (Lees et al., 2009), and these are believed to be specifically sensitive to mitochondrial dysfunction (Bose and Beal, 2016), whether induced by toxins (Terron et al., 2018), genetic predisposition (Deng et al., 2018), or somatic accumulation of genetic damage, such as to mitochondrial DNA (Bender et al., 2006; Kraytsberg et al., 2006). Many questions remain open, as to why DA neurons are especially sensitive to mitochondrial dysfunction and how this results in loss of function and eventually produces disease.

The fruit fly *Drosophila melanogaster* has emerged as a useful animal model for studying pathological processes and possible treatment options for neurological disorders (Jeibmann and Paulus, 2009; McGurk et al., 2015), including the role of mitochondria (Guo, 2012). DA neurons in *Drosophila* have been implicated in a number of behavioral processes, including feeding, sleep, and locomotion, and fly models of locomotor dysfunction strongly support a role for mitochondrial stress in the phenotype.

In the *Drosophila* brain there are rather few DA neurons, mostly located in anatomically distinct clusters specified during embryogenesis (Hartenstein et al., 2017). Because of their functional and anatomical similarities with DA neurons in vertebrates, they are of great interest in the fields of neurodegeneration and other neurological disorders, both movement related and psychiatric (White et al., 2010). In addition to the panoply of genetic tools available to probe cellular and physiological processes in the fly, a number of simple, quantifiable behavioral paradigms are well established in *Drosophila*. Prominent among them is the negative geotaxis (climbing) assay, which has been widely used to measure locomotor competence in adults. It is frequently considered a marker for neurodegeneration, especially when used to score the effects of manipulating genes implicated in human neurodegenerative disease (Fernius et al., 2017). These include, for example, the Alzheimer-associated beta-amyloid peptide (Marcora et al., 2014), the familial PD gene *Pink1* (Guo, 2012), or the *Drosophila* homologue of *FUS* (*cabeza*), implicated in one form of ALS (Frickenhaus et al., 2015).

In a previous study, we observed that pan-neuronal RNAi-mediated knockdown of various different subunits of cytochrome oxidase (COX, respiratory complex IV, cIV), the terminal enzyme of the mitochondrial respiratory chain, results in a severe locomotor impairment as measured by the negative geotaxis assay (Kemppainen et al., 2014). The phenotype was most severe when “core” subunits of the complex, such as COX4, were targeted, with all flies dying at the early larval stages. However, when peripheral subunit COX7A was targeted specifically, flies generally died as pupae, and even eclosed as adults if COX7A knockdown was confined to neurons, or if the alternative oxidase (AOX) from *Ciona intestinalis* was expressed to provide a metabolic bypass for cIV (Kemppainen et al., 2014; Andjelković et al., 2015).

The functional role of the COX7A subunit is not fully known, although it is clearly required for cIV assembly and full COX activity (reviewed by Mansilla et al., 2018). One mammalian paralog, Scafi (also known as COX7A-related protein, COX7RP, or COX7AL2), has been implicated in the formation of respiratory supercomplexes (Lapuente-Brun et al., 2013; Ikeda et al., 2013).

In addition to the canonical COX7A gene, the *Drosophila* genome has two homologs thereof, namely, COX7AL, expressed only in testis, and CG34172, expressed mainly in adult carcass and other muscle-containing tissues (heart, gut), with all three showing greater similarity to the two mammalian COX7A isoforms, COX7A1 (heart) and COX7A2 (liver) than to Scafi, and therefore considered as standard subunits of cIV. Given that CG34172 is only minimally expressed in the nervous system, combined with the specificity of available GAL4 drivers, this affords an opportunity to study the biological effects of neural knockdown of COX without having a major effect on muscle or any other tissues.

Given the prevailing view in the field that DA neurons are especially vulnerable to mitochondrial dysfunction, we set out to investigate how COX deficiency impacts them in the fly, taking as our starting point the previous observations on pan-neuronal COX7A knockdown (Kemppainen et al., 2014; Andjelković et al., 2015). Although we observed a clear decrease in the level of the key DA biosynthetic enzyme tyrosine hydroxylase (TH), these effects were found to be secondary in nature. Although such findings cannot be directly translated to mammals, they highlight the need to re-examine thoroughly the link between mitochondrial dysfunction, DA neuron degeneration, and locomotor impairment in other contexts.

## Results

### Pan-Neuronal Knockdown of COX7A Decreases TH Expression in DA Neurons

We first confirmed the effectiveness of RNAi directed against COX7A, using VDRC (RRID:SCR_013805) line 106661 plus the pan-neuronal *elav-GAL4*^*C155*^ driver, in combination with *UAS-Dcr2* to potentiate its effects on locomotion. The driver and *UAS-Dcr-2* alone produced no locomotor impairment (Andjelković et al., 2015), so this was included as a control in the experiments to profile the effects of knockdown. Western blots showed that pan-neuronal COX7A knockdown resulted in an ∼50% decrease in the amount of COX4 protein in fly heads (Figure S1A), a proxy for assembled cIV, and a similar decrease in respiratory function (Figure S1B), showing clearly that the phenotype obtained is associated with mitochondrial dysfunction.

Previous studies (Kemppainen et al., 2014) found no gross anatomical abnormalities from COX knockdown during development, implying that it does not result in substantial cell death. To profile ongoing cell death in the brains of flies with pan-neuronal COX7A knockdown, we used two approaches. TUNEL staining revealed a very low number of apoptotic cells, despite strong signals in the positive control (Figure S1C). However, although low, the number of TUNEL-positive cells in knockdown brains was significantly higher than in controls (Figure S2A) and included both neuronal and glial cells (Figure S2B). Using the Apoliner reporter system (Bardet et al., 2008), in which apoptotic cells are marked by the nuclear translocation of GFP, we observed only very few such examples (Figures S3 and S4). We also observed no increase in signal compared with controls, when knockdown brains were stained with dihydroethidium (Figure S5) as a crude marker for superoxide production.

**Figure 1:**
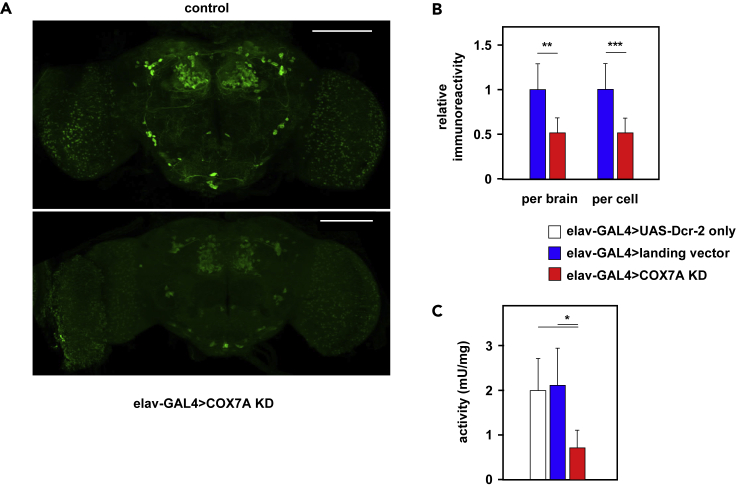
Pan-Neuronal Knockdown of COX7A Leads to TH Deficiency (A) Immunohistochemistry for TH on brains from male flies with and without pan-neuronal knockdown of COX7A (representative images). Genotypes as follows–control (designated as elav-GAL4>landing vector in B and C): *elav-GAL4*^*C155*^, *UAS-Dcr-2* / Y*; P*{*attP, y*^*+*^*, w*^*3*^}*/+*. elav-GAL4>COX7A KD: *elav-GAL4*^*C155*^, *UAS-Dcr-2* / Y*; UAS-RNAi*^*COX7A*^ / *+*, where *UAS-RNAi*^*COX7A*^ denotes the dsRNA-encoding insertion in VDRC line 106661. Rabbit anti-TH antibody, 1:500, used with Alexa 488 goat anti-rabbit secondary antibody, 1:500. Scale bars, 100 μm. Note that batches of control and knockdown brains were processed in parallel using the same reagents. Counterstaining for Elav (Figure S6) indicated that low TH signal was not due to a general issue with antibody penetration. The range of staining intensities likely reflects both technical variability in sample preparation and visualization, as well as biological variation. (B) Quantitation of TH signal from immunohistochemistry on multiple specimens of the types shown in (A). Signal was quantitated in dopaminergic neurons of the PAL (protocerebral anterior lateral) cluster and plotted as mean signal intensity (±SD) for each brain (left-hand columns: elav-GAL4>landing vector controls, n = 4, elav-GAL4>COX7A KD, n = 8) or for each analyzed cell considered as a separate data point (right-hand columns: elav-GAL4>landing vector, n = 11, elav-GAL4>COX7A KD, n = 27). Note that all flies also carried *UAS-Dcr-2*. The PAL cluster was selected, being easily identified, close to the anterior surface of the brain, and comprising only a few cells. (C) TH activity from extracts of female fly heads of the indicated genotypes: elav-GAL4>UAS-Dcr-2 only – *elav-GAL4*^*C155*^, *UAS-Dcr2*, elav-GAL4>landing vector – *elav-GAL4*^*C155*^, *UAS-Dcr-2*; *P*{*attP, y*^*+*^*, w*^*3*^} / + and elav-GAL4>COX7A KD – *elav-GAL4*^*C155*^, *UAS-Dcr-2*; *UAS-RNAi*^*COX7A*^/+. Significant differences (Student's t test or, where more than two classes were compared, one-way ANOVA with Tukey *post hoc* HSD test) denoted as ∗, ∗∗, and ∗∗∗, representing p < 0.05, 0.01, and 0.001, respectively. See also Figures S1–S5 for validation of knockdown and its effects and Figure S6 for validation of immunohistochemistry.

Pan-neuronal COX7A knockdown produced no obvious difference in the number of TH-positive neurons but nevertheless revealed a marked decrease in TH signal (Figure 1A). To quantify the effect, we focused on a specific cluster of DA neurons, the PAL (protocerebral anterior lateral) cluster, avoiding issues arising from the differing signal intensities from different such clusters (Mao and Davis, 2009). The average signal intensity, whether computed per brain or per cell (Figure 1B), showed a significant (∼50%) decrease in knockdown brains. TH activity, measured in protein extracts from isolated heads, showed a similar decrease (Figure 1C). Decreased TH activity in knockdown brains is consistent with a specific effect on DA neurons.

### Locomotor Defect Is Caused by COX7A Knockdown in Specific Groups of Neurons

**Figure 2:**
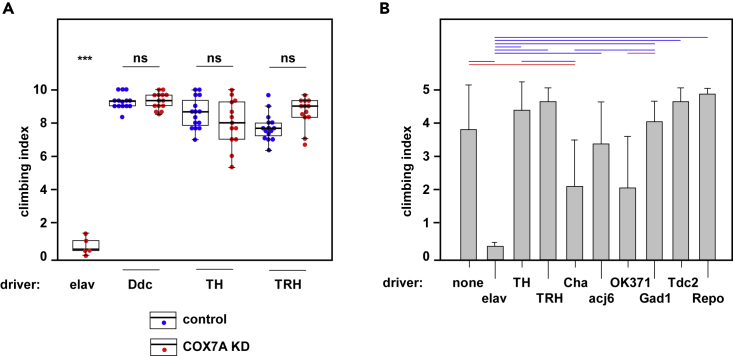
Locomotor Impairment Is due to COX7A Knockdown in Neurons of Specific Neurotransmitter Identities Climbing index for COX7A-KD flies derived by crossing the indicated GAL4 driver or control males (homozygous or balanced, as indicated in Transparent Methods) to *UAS-RNAi*^*COX7A*^; *UAS-Dcr-2* females or (A, controls) to *UAS-Dcr-2* females without the RNAi construct. (A) Boxplots indicate medians (bold lines), 25^th^ and 75^th^ percentiles (box limits), and 1.5 times the interquartile range (Tukey-style whiskers, truncated where they would cross the zero or maximum lines) for batches of 10 virgin female flies. Colored dots represent individual data points for each batch (means of three tests) as indicated. Significant differences (one-way ANOVA with Tukey *post hoc* HSD test), indicated as ∗∗∗, p < 0.001 for comparison with all other data classes, ns, p > 0.05 for the pairwise comparisons indicated. Other comparisons omitted for clarity. Note that the *elav*-GAL4 driver was included here as a positive control. (B) Means ± SD (n ≥ 4, except for *repo*-GAL4, n = 3) for batches of five virgin female flies. Statistical analysis (one-way ANOVA with Tukey *post hoc* HSD test; significant differences indicated by red and blue bars, denoting p < 0.05 and p < 0.01, respectively. For repeat experiments using *Cha*-GAL4 and OK371 drivers, alongside *UAS-Dcr-2* controls, see Figures 3B and 3C. Note that *TH*-GAL4 drives expression only poorly, if at all, in a small subset of DA neurons that are, conversely, targeted by *Ddc*-GAL4 (Riemensperger et al., 2013); both drivers are therefore needed to cover all DA neurons. Based on GFP controls, drivers were active in cells of the predicted identity (Figures S7–S9), although the *TRH*-GAL4 driver did drive expression in a small number of TH-positive (i.e., DA) neurons (Figure S9B). Effects in DA neurons were not simply delayed (see Figure S10).

**Figure 3:**
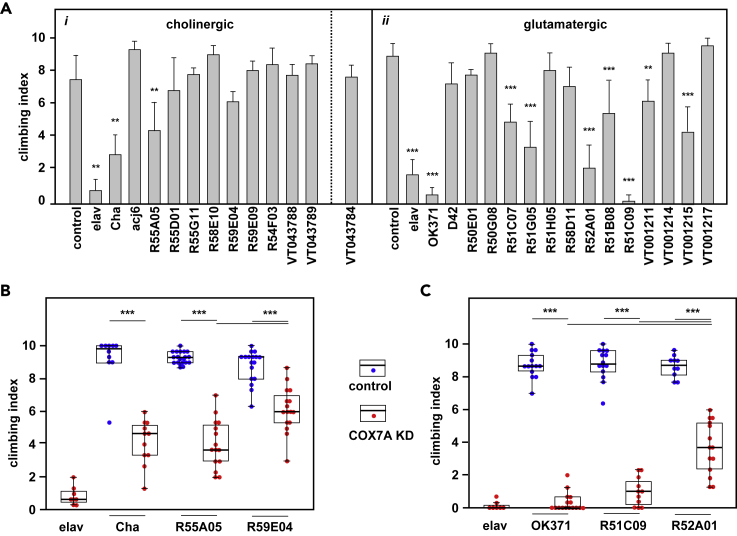
Locomotor Impairment due to COX7A Knockdown in Specific Sub-groups of Cholinergic and Glutamatergic Neurons Climbing index for COX7A-KD flies derived by crossing the indicated GAL4 drivers, from the FlyLight (Jenett et al., 2012) and Vienna Tiles collections (Tirian and Dickson, 2017), or control males to *UAS-RNAi*^*COX7A*^; *UAS-Dcr-2* females or (B, controls) to *UAS-Dcr-2* females without the RNAi construct. (A) Means ± SD (n ≥ 4) for batches of 10 virgin female flies. Significant differences (one-way ANOVA with Tukey *post hoc* HSD test) indicated as ∗, ∗∗, and ∗∗∗, denoting p < 0.05, 0.01, and 0.001, respectively, in comparison with controls. Other comparisons omitted for clarity. (i) and (ii) represent separate series of experiments for cholinergic and glutamatergic drivers, as indicated. As denoted by the dotted line, for logistical reasons, experiments with the cholinergic VT043784 driver were performed and analyzed together with those using the glutamatergic drivers shown in (ii). (B and C) Boxplots for selected (B) cholinergic and (C) glutamatergic drivers, using the same conventions and statistical analyses as in Figure 2A.

To test whether DA neurons are the functionally relevant target in generating locomotor defect by COX7A knockdown, we used the drivers *TH*-GAL4, which selectively targets DA neurons, *Ddc*-GAL4, which targets both serotonergic and DA neurons, and *TRH*-GAL4, specific for serotonergic neurons. To ensure effective knockdown, we included *UAS-Dcr-2* in all knockdown experiments. As negative controls we combined *UAS-Dcr-2* with each driver (except for *elav*-GAL4, which was included here as a positive control). Neither of the DA drivers (nor the one targeting only serotonergic neurons) gave a significant locomotor defect (Figure 2A). Using a wider selection of drivers (Figure 2B), we observed a significant locomotor defect when COX7A knockdown was driven by *Cha*-GAL4 (cholinergic neurons) or by OK371 (glutamatergic neurons) but not by *acj6*-GAL4 (Figures 2B and 3A), targeting a restricted group of cholinergic neurons (Lee and Salvaterra, 2002). Drivers targeting GABAergic or octopaminergic neurons or glia also had no effect on locomotor activity (Figure 2B).

Using GFP as a reporter we observed that the *Cha*-GAL4 driver is not fully specific for cholinergic neurons (see Figures S11–S13). We therefore proceeded to test additional drivers, incorporating only fragments of the *ChAT* gene, to identify more restricted subsets of cholinergic neurons responsible for the locomotor phenotype and confirm their neurotransmitter identities. Given the large number of neurons targeted by OK371 (Figure S14), we took a similar approach to narrow down the susceptible glutamatergic neurons, using drivers incorporating fragments from the *VGlut* gene. We identified strong candidates from both sets (Figure 3A) and confirmed them against *UAS-Dcr-2* controls (Figures 3B and 3C). In summary, the locomotor defect produced by neuronal knockdown of COX7A is dependent on restricted subsets of cholinergic and glutamatergic neurons, rather than on DA neurons as initially assumed.

### Several Largely Non-overlapping Sets of Neurons Are Susceptible to COX7A Knockdown

**Figure 4:**
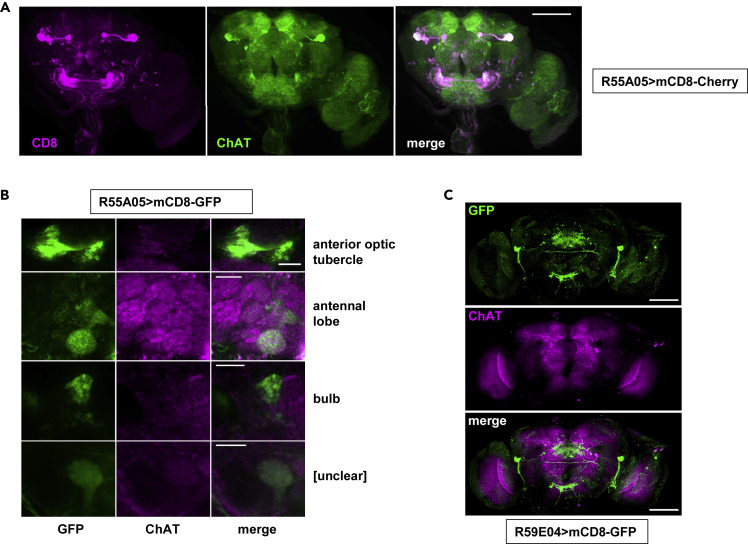
Neuronal Targets of GAL4 Drivers R55A05 and R59E04 Immunohistochemistry of brains from individual flies expressing mCD8 reporters under the control of the GAL4 drivers indicated. (A) Single optical section co-stained for CD8 (rat anti-CD8, 1:200, followed by Alexa 568 goat anti-rat) and ChAT (mouse anti-ChAT, 1:100, followed by Alexa 488 goat anti-mouse). Scale bar, 100 μm. For equivalent maximum intensity projection of the whole brain stained for CD8, see Figure S15. (B) Higher magnification of some specific brain regions co-stained for GFP (rabbit anti-GFP, 1:1,000, followed by Alexa 488 goat anti-rabbit) and ChAT (mouse anti-ChAT, 1:100, followed by Alexa 647 goat anti-mouse). Scale bar, 20 μm. These are maximum intensity projections compiled from a few adjacent optical sections, sufficient to reveal the shapes of the co-stained structures. The fourth structure (marked “[unclear]”) shows a structure that we were unable to identify with confidence from Virtual Fly Brain (v2.virtualflybrain.org). Note that, because overlap appeared so extensive in the first experiment (Figure 4A), we here combined a green and a far-red fluor to minimize any possible bleeding between channels. (C) Maximum intensity projection stained for GFP (rat anti-CD8, 1:200, followed by Alexa 647 goat anti-rat) and for ChAT (mouse anti-ChAT, 1:100, followed by Alexa 488 goat anti-mouse). Scale bar, 100 μm. See also Figures S16–S20: note that these two drivers targeted largely non-overlapping sets of cholinergic neurons, whereas neither was active in DA neurons.

For further study we selected the two cholinergic (R55A05 and R59E04) and the two glutamatergic drivers (R52A01 and R51C09) giving the strongest locomotor impairment. The expression patterns of these drivers are already known and were verified using fluorescent reporters before proceeding. Expression driven by the cholinergic driver R55A05 labeled a number of specific brain structures (Figures 4A and 4B), with widespread colocalization with ChAT, although in some brain regions the ChAT signal was very weak. There was no convincing colocalization with TH or other specific neural markers (see Figures S16 and S17). The second cholinergic driver (R59E04) exhibited an almost completely distinct expression pattern (Figures 4C and S18), with variable and incomplete colocalization with ChAT (Figures 4C and S19), and no overlap with TH (see Figure S20).

**Figure 5:**
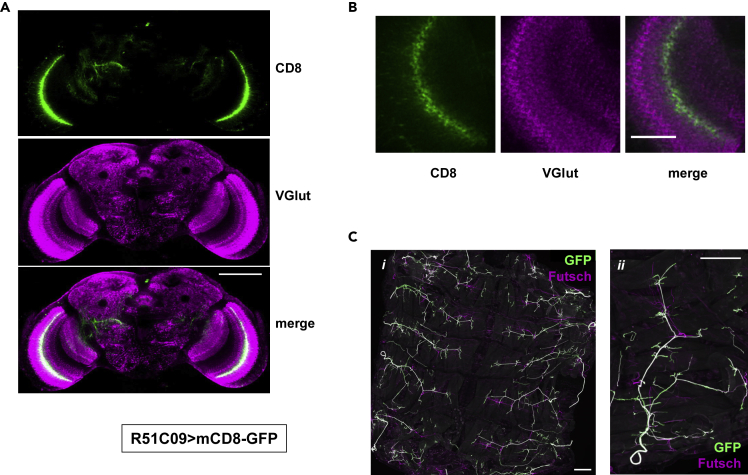
Neuronal Targets of GAL4 Driver R51C09 Immunohistochemistry of (A) whole brains and (B) portion of optic lobes from adult females, and (C) larval subcuticular neurons, from *Drosophila* expressing mCD8-GFP under the control of GAL4 driver R51C09. (A and B) Single optical sections of samples co-stained for CD8 and VGlut. Scale bar, 100 μm in (A) and 20 μm in (B). (C) Maximum intensity projections of samples co-stained for GFP and the microtubule-binding protein Futsch. (*i*) Low magnification, anterior to the top, dorsal midline in the middle. (*ii*) Higher magnification, dorsal side to the top, anterior to left. Scale bars, 200 μm. Note that there was no colocalization with GABA or TH (Figure S21).

**Figure 6:**
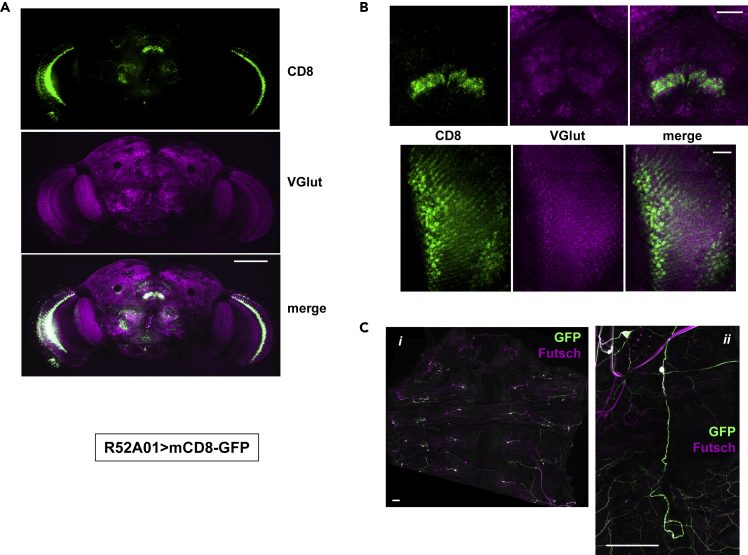
Neuronal Targets of GAL4 Driver R52A01 Immunohistochemistry of (A) whole brains and (B) selected brain areas from adult females, and (C) larval subcuticular neurons, from *Drosophila* expressing mCD8-GFP under the control of GAL4 driver R52A01. (A and B) Single optical sections of samples co-stained for CD8 and VGlut. Scale bar, 100 μm in (A) and 20 μm in (B). (C) Maximum intensity projections of samples co-stained for GFP and the microtubule-binding protein Futsch. (*i*) Low magnification, anterior to the top, dorsal midline in the middle. (*ii*) Higher magnification, dorsal side to the top, anterior to left. Scale bars, 100 μm. Note that there was no colocalization with GABA or TH (Figure S22).

The two selected glutamatergic drivers showed limited expression in the brain (Figures 5 and 6), most prominently in the optic lobe (Figures 5A and 6A). Because motoneurons are glutamatergic in *Drosophila*, we investigated whether they were targets of either driver, by examining the subcuticular neurons of the larval PNS, where motoneurons are easily distinguished. R51C09 directed expression in a pair of prominent axons, repeating in each hemisegment of the trunk (Figure 5C). Their cell bodies were absent, consistent with their being motoneurons, since motoneuron cell bodies lie in the ventral nerve cord, which was removed in the dissection of the larvae. R52A01 also directed expression in prominent subcuticular neurons repeating in each hemisegment (Figure 6C). As the cell bodies of these neurons were present in the dissected larvae, they cannot be motoneurons and are almost certainly sensory neurons. Using a dual-reporter system we confirmed that there was very little overlap in the brain-expression patterns of the two selected cholinergic drivers or the two selected glutamatergic drivers (see Figures S23 and S24). We conclude that locomotor impairment can be brought about by mitochondrial dysfunction in any of several largely independent sets of neurons.

### Only a Minor Fraction of COX7A Knockdown-Sensitive and DA Neurons Interact Directly

**Figure 7:**
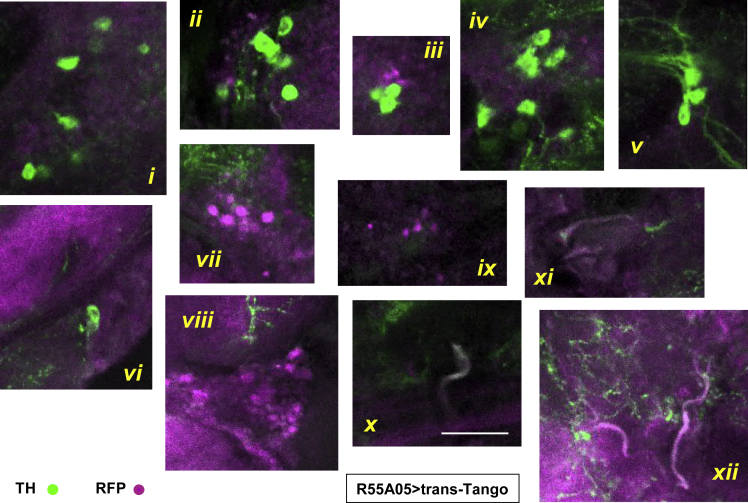
A Minor Fraction of R55A05 Post-synaptic Neurons Are Dopaminergic Immunohistochemistry for TH (green, 1:500) and RFP (magenta) from brain of a female fly post-synaptically expressing RFP under the control of trans-Tango, driven by R55A05. Genotype: *UAS-myrGFP*, *QUAS-mtdTomato-3xHA* / +; *trans-Tango* / +; *R55A05-GAL4* / +. Zoomed images from individual optical sections; scale bar, 20 μm (all images to same scale). Images optimized for brightness and contrast but not manipulated in any other way. Overlap signals (white, pale magenta, and pale green, panels *viii*, *x–xii*) represent only a small minority of cells, not including (panels *i*–*vi*) the major clusters of DA neurons. Nevertheless, strong post-synaptic signals are seen both in TH-negative cell bodies (panels *ii*, *iii*, *viii*, *ix*) and in regions with abundant neurites (panels *vi*–*viii*, *xii*). For side-by-side comparisons of signals from the separate channels see Figures S27–S29. For the whole brain images from which these panels were derived, see Video S1 and Figures S30 and S31.

Based on the above findings, the neurons vulnerable to mitochondrial dysfunction are not dopaminergic, even though TH was depleted by pan-neuronal knockdown of COX7A. This raises the possibility that DA neurons and those sensitive to COX7A knockdown are part of the same neural circuits controlling locomotion. Consistent with this, dendritic projections from sensitive cholinergic neurons are found in the same areas of the brain as those from TH-positive neurons (Figures S25 and S26), although this does not demonstrate direct interaction. Moreover, using the *trans*-Tango reporter system (Talay et al., 2017), we found only a very low proportion of TH-positive neurons connected post-synaptically to those targeted by the R55A05 driver (Figure 7, Figures S27–S31, Video S1), and this did not include any of the prominent clusters of DA neurons where the loss of TH signal was quantified in earlier experiments. Any connections between the cholinergic neurons targeted by this driver and major groups of DA neurons involved in locomotor functions must therefore be indirect.

### Apoptosis of Neurons Sensitive to COX7A Knockdown Is Lethal

Although we found no evidence of widespread cell death in the brains of adult COX7A knockdown flies, this does not exclude death of vulnerable neurons during development. To test this, we set up crosses using males from each of the selected driver lines mated with balanced females expressing Hid, a potent inducer of apoptosis (Sandu et al., 2010), under GAL4 control (see Transparent Methods). In the case of all four selected drivers, all eclosing progeny carried the balancer markers. Therefore, the death of the targeted cells is developmentally lethal, excluding widespread neuronal death as the mechanism by which COX7A knockdown results in locomotor impairment.

### TH Deficiency Does Not Correlate with Locomotor Defect in COX7A Knockdown Flies

**Figure 8:**
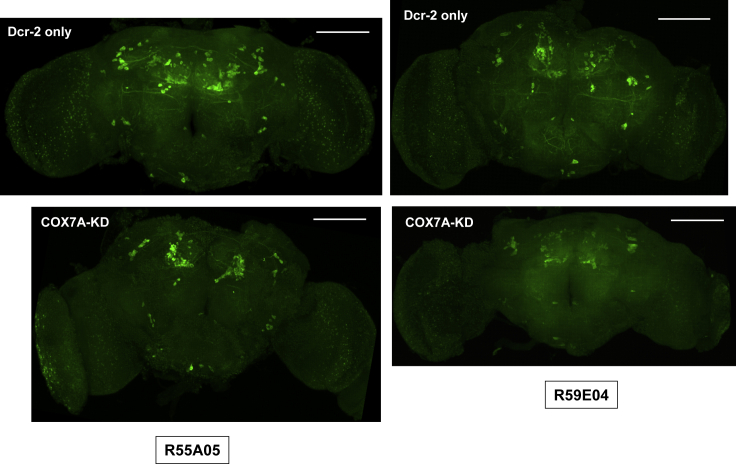
TH Deficiency Resulting from COX7A Knockdown in Subsets of Cholinergic Neurons Immunohistochemistry for TH (1:500) in brains from flies with COX7A knockdown or controls with only Dcr-2 overexpression, as indicated, using the GAL4 drivers shown (maximum intensity projections). Genotypes – Dcr-2 only: +; *R55A05-GAL4* (or *R59E04-GAL4*)/*UAS-Dcr-2*, COX7A-KD: *UAS-RNAi*^*COX7A*^ / +; *R55A05-GAL4* (or *R59E04-GAL4*)/*UAS-Dcr-2*. Scale bars, 100 μm. Contrast and brightness have been similarly adjusted in each image, giving uniform background fluorescence. See Figures S32–S35 for these and images of other brains from flies of the same genotypes, alongside images showing counter-stain for Elav.

**Figure 9:**
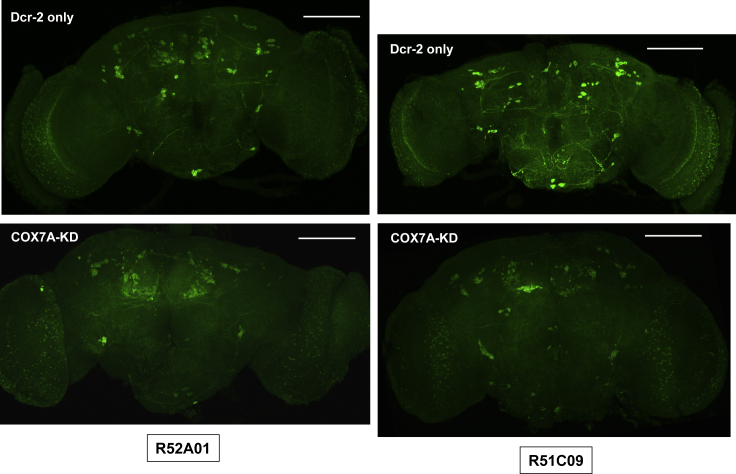
TH Deficiency Resulting from COX7A Knockdown in Subsets of Glutamatergic Neurons Immunohistochemistry for TH (1:500) in brains from flies with COX7A knockdown or controls with only Dcr-2 overexpression, as indicated, using the GAL4 drivers shown (maximum intensity projections). Genotypes – Dcr-2 only: +; *R52A01-GAL4* (or *R51C09-GAL4*)/*UAS-Dcr-2*, COX7A-KD: *UAS-RNAi*^*COX7A*^ / +; *R52A01-GAL4* (or *R51C09-GAL4*)/*UAS-Dcr-2*. Scale bars, 100 μm. Contrast and brightness have been similarly adjusted in each image, giving uniform background fluorescence. See Figures S36–S39 for these and images of brains from flies of the same genotypes, alongside images showing counterstain for Elav.

Given that the connections between targeted and DA neurons appear to be mostly indirect, we tested whether the TH deficiency produced by pan-neuronal COX7A knockdown could be recapitulated by targeting COX7A knockdown to specific groups of neurons, using the selected drivers. The R55A05 GAL4 driver gave, at most, a modest, diminution of TH signal (Figure 8), whereas that produced by the R59E04 driver, with a weaker locomotor effect, was generally more pronounced (Figure 8). Similar findings were obtained using the glutamatergic drivers (Figure 9). These findings imply that TH deficiency and impaired locomotion are not strictly correlated and suggest that TH deficiency may not be systematically instrumental in the behavioral phenotype.

## Discussion

In this study we showed that several groups of susceptible cholinergic and glutamatergic neurons, but not dopaminergic (DA) neurons, underlie the locomotor impairment brought about by RNAi-mediated knockdown of a subunit of cytochrome *c* oxidase in the *Drosophila* nervous system. This challenges the widely held assumption that DA neurons are key targets of mitochondrial dysfunction, leading to impaired locomotor function, at least in the fly.

### Mitochondrial Dysfunction in Specific Cholinergic and Glutamatergic Neurons Is Instrumental in Locomotor Impairment

Pan-cholinergic and pan-glutamatergic COX knockdown both produced a severe locomotor phenotype. We used drivers targeting much more restricted subsets of neurons to narrow down this vulnerability and exclude cells with other neurotransmitter identities. However, the two cholinergic drivers giving significant locomotor impairment had almost completely non-overlapping expression profiles, with the one showing the stronger effects (R55A05) expressed in numerous brain regions. Thus, it is not yet possible to say which such regions are the more critical. On the glutamatergic side, the two strongest drivers also showed only partially overlapping expression patterns in the brain (Figures 5 and 6), with prominent expression also in different cells of the larval peripheral nervous system (PNS), one in motoneurons (Figure 5C) and the other in a subclass of sensory neurons (Figure 6C). Note also that COX7A knockdown driven by D42, which targets at least some adult motoneurons (Yeh et al., 1995; Sanyal et al., 2003), gave no such phenotype (Figure 3A). Locomotor impairment may therefore be due to mitochondrial dysfunction in a variety of specific cells in the brain, PNS or both, in diverse combinations.

Our study illustrates the limitations of the widely used negative geotaxis assay, in that a similar locomotor impairment can be independently produced by a primary defect in any of a number of distinct subsets of neurons, with different functions and neurotransmitter identities, possibly not involving a uniform molecular mechanism. Experiments using the assay should therefore be interpreted with caution. The current study also prompts an analysis of a broader range of behavioral phenotypes in flies with neuronal COX deficiency.

### Effects on DA Neurons

*Ddc*-GAL4 and *TH*-GAL4 most strongly target different subsets of DA neurons (Riemensperger et al., 2013), but neither was able to produce locomotor impairment when used to drive COX7A knockdown. This contrasts with the findings of Riemensperger et al. (2013), who found that the expression of the PD-associated human α-synuclein A30P variant produced a locomotor defect when expressed using the *Ddc*-GAL4 but not the *TH*-GAL4 (or *TRH*-GAL4) drivers. Although we cannot exclude that simultaneous COX deficiency in both groups of DA neurons is required to produce locomotor impairment, we did not detect TH-positive cells among those targeted by any of the four drivers that did produce the phenotype. Therefore, it is highly unlikely that COX deficiency in DA neurons contributes cell-autonomously to a locomotor phenotype, except perhaps in very aged flies.

Pan-neuronal COX7A knockdown did lead to a deficiency of TH, but its relationship to locomotor impairment is unclear. Although a non-cell-autonomous impairment of DA neuron function is a plausible explanation for our findings, TH deficiency might also be a secondary effect of dysfunction in relevant circuits that is not instrumental in the locomotor phenotype. Consistent with this, the individual drivers tested had varying and more subtle effects on TH levels (Figures 8 and 9). The two phenotypes (TH deficiency and impaired locomotion) are thus not tightly correlated.

### Locomotor Effects of Mitochondrial Dysfunction Are Not Mediated by DA Neuronal Death

We were unable to detect a prominent signature of cell death associated with pan-neuronal knockdown of COX7A. Moreover, there was no obvious difference in the number of PAL neurons, in contrast to the findings of Humphrey et al. (2012). Although we might have missed a very small decrease in the number of DA neurons, it is unlikely to account for the severe locomotor impairment produced. Although long-term loss of inputs to these neurons may result in their eventual loss, our data implicate COX deficiency in other group of neurons, not DA cell death, as the underlying cause of locomotor impairment.

### Comparison with Previous Studies of Mitochondrial Dysfunction in DA Neurons

Our findings contrast with previous reports of progressive DA-cell loss and impaired locomotion, when a mitochondrial insult was targeted to DA neurons using the same *TH*-GAL4 driver as here (Humphrey et al., 2012), involving RNAi against the catalytic subunit of the mitochondrial DNA polymerase, Polγ (*tamas*). Conversely, applying *Cha*-GAL4 produced no such phenotype, whereas in the current report, RNAi against a subunit of cIV produced an opposite result. The discrepancy with our findings could have various explanations, which need to be explored in future studies.

First, the phenotype described by Humphrey et al. (2012) is a progressive one with late onset. *tamas* knockdown flies initially showed only a very mild degree of locomotor impairment, but that steadily worsened during adult life, whereas COX7A knockdown had dramatic (but stable) effects in young adult flies. The two phenotypes studied, i.e., age-related neurodegeneration and developmental locomotor impairment, are fundamentally different and may be produced by entirely different mechanisms. Note that most cases of heritable mitochondrial dysfunction affecting the CNS in humans manifest in infancy and are often fatal within the first weeks or months of life (Gorman et al., 2016).

Second, *tamas* and COX7A knockdown should result in quite distinct metabolic effects. The latter results in a clear but isolated deficiency of cytochrome oxidase, whereas the former should produce mtDNA depletion, affecting all four enzymatic components of the oxidative phosphorylation (OXPHOS) system that are dependent on mtDNA-encoded gene products. Different classes of neuron may have quite distinct susceptibilities to these different metabolic stresses. AOX expression in the Polγ model (Humphrey et al., 2012) also had much less dramatic effects than in the COX7A model (Kemppainen et al., 2014; Andjelković et al., 2015).

Finally, in a different study (Bahhir et al., 2019), stress imposed upon mtDNA by the ubiquitous expression of a bacterial type I restriction endonuclease produced a range of metabolic abnormalities unrelated to OXPHOS, including decreased levels of dopamine, and accompanying behavioral changes, notably in feeding. Dietary supplementation with L-DOPA restored wild-type feeding behavior and delayed the onset of lethality in the model. Although the detailed mechanism of the effect remains to be elucidated, mitochondrial dysfunction and its effects on DA-producing cells clearly should not be considered as a single, “all or none” phenomenon.

### Possible Relevance to Neurological Disease

Owing to the involvement of mitochondrial gene products and toxin targets in human neurological disease, combined with the evidence for DA neuron degeneration as a pathomechanism in progressive locomotor disorders, it is widely assumed that DA neurons are particularly vulnerable to mitochondrial dysfunction. Although our study may not be directly translatable to the human context, relates to only one component of the OXPHOS system (cIV), and concerns a developmental rather than a degenerative phenotype, neurological involvement is a common feature of early-onset mitochondrial disease (Gorman et al., 2016). Our findings indicate that the direct neuronal targets of mitochondrial dysfunction leading to locomotor impairment, at least in early-onset disease, may not be DA neurons, but some other class of neural cells, with any effects on DA neurons being secondary to the underlying defect. Mitochondrial dysfunction has also been widely proposed as an instrumental factor in a variety of neurodegenerative diseases (Smith et al., 2019; Lim et al., 2020). Neuronal classes other than DA neurons have recently been implicated in neurodegenerative disease in humans (Liu, 2020). Our findings provide a possible model whereby mitochondrial dysfunction in such cells might be an underlying factor in the pathology, operating long before overt neurodegeneration manifests.

*Drosophila* has proven to be a useful model for understanding fundamental biological processes in animals, such as the axial patterning of the nervous system (Estacio-Gómez and Díaz-Benjumea, 2014) or circadian behavior (Dubowy and Sehgal, 2017). In this light, and considering the conserved metabolism and function of DA neurons, it would be appropriate to re-examine this vulnerability to mitochondrial dysfunction of DA and other classes of neuron in other organisms, including mammals, and to test the effects of different types of mitochondrial insult, bearing in mind that the neuroanatomical differences (e.g., in size) between fly and mammalian brains may dictate a distinct outcome.

### Limitations of the Study

As discussed above, the study was conducted on a single model organism (*Drosophila melanogaster*), using only one, albeit common, type of mitochondrial dysfunction, COX deficiency, here resulting from knockdown of a COX subunit. It focused on a single behavioral readout, locomotor impairment, and was concerned only with an intrinsic, developmentally determined, neural phenotype, rather than with age-related degeneration. Finally, although excluding a widely assumed mechanism whereby mitochondrial dysfunction results in locomotor impairment (i.e., a cell-autonomous effect in DA neurons), it leaves open the molecular mechanism(s) that do operate here.

### Resource Availability

#### Lead Contact

Further information and requests for resources and reagents should be directed to and will be fulfilled by the Lead Contact, Howard T. Jacobs (howard.jacobs@tuni.fi).

#### Materials Availability

The study did not generate any new, unique reagents. All materials used in the work were sourced from public or commercial resources (*Drosophila* stock centers, antibody suppliers, manufacturers of standard laboratory equipment and reagents), as described under Transparent Methods (see Supplemental Information). If any such reagents or strains should become unavailable from these sources, the authors will be happy to assist in procuring them elsewhere, as far as they are able.

#### Data and Code Availability

This study did not generate or use any new datasets or machine code. All of the primary experimental data used in compiling this paper are included in the figures and Supplemental Information. For images presented here as maximum projections, the original image stacks are available upon request, although they do not provide any salient additional information relevant to the study.

## Methods

All methods can be found in the accompanying Transparent Methods supplemental file.

Video S1. Post-synaptic Connections to DA Neurons, Related to Figure 7

## Supplemental Information

Document S1. Transparent Methods, Figures S1–S39, and Table S2

Table S1. *Drosophila* Strains Used in the Study, Related to Figures 1–9 and Transparent Methods

## Footnotes

Supplemental Information can be found online at https://doi.org/10.1016/j.isci.2020.101362.
