# Supplementary material for: Effects on Dopaminergic Neurons Are Secondary in COX-Deficient Locomotor Dysfunction in Drosophila
Source: iScience. 2020 Jul 12;23(8):101362. doi: 10.1016/j.isci.2020.101362 (PMC7394922; doi:10.1016/j.isci.2020.101362)
Supplement: Document S1. Transparent Methods, Figures S1–S39, and Table S2 [file mmc1.pdf]

iScience, Volume *23*

Supplemental Information

Effects on Dopaminergic Neurons Are Secondary in COX-Deficient Locomotor Dysfunction in *Drosophila*

Cagri Yalgin, Bohdana Rovenko, Ana Andjelković, Margot Neefjes, Burak Oymak, Eric Dufour, Ville Hietakangas, and Howard T. Jacobs

SUPPLEMENTAL INFORMATION

Contents

Within this file:

Transparent Methods

Supplemental References

Supplemental Tables

Table S2

Legends to Supplemental Figures

Supplemental Figures

Figures S1-S39

In separate files

Supplemental Table S1 (separate .xls file)

Supplemental Movie S1 (separate .avi file)

TRANSPARENT METHODS

Drosophila strains and culture

Drosophila strains used in the study, and their sources, are summarized in Table S1. Flies were maintained in standard high-sugar medium (Kemppainen et al., 2016) on a 12 h light/dark cycle at 25 °C. Crosses were generally implemented in triplicate, with flies tipped to new vials on three successive days after mating.

Repetitive iterative negative geotaxis (RING) assay

Eggs from parents crossed two days earlier were collected over three consecutive days, and cultured at 25 °C until eclosion. Virgin female progeny were then kept in food vials at 25 °C until day 3, when they were sorted into batches of 10 flies and maintained at 25 °C for a further 2 days. On day 5, flies were tipped from vials to 50 ml Falcon tubes without the use of CO_2 (Bartholomew et al., 2015). Up to six Falcon tubes were placed in rows as described (Nichols et al., 2012). After a 10 min waiting period, flies were tipped down and their subsequent behavior recorded using a DFK 21AF04 camera (The Imaging Source, Bremen, Germany) and Media Recorder 2 software (Noldus, Wageningen, Netherlands). The climbing index (Kemppainen et al., 2014) for each vial was manually calculated from recordings as the mean number of flies which climbed 6 cm in 10 s in three trials. Climbing indices from different genotypes were compared by one-way ANOVA with Tukey *post hoc* HSD test. Box plots were drawn with BoxPlotR (boxplot.tyerslab.com), with Tukey-style whiskers (Krzywinski and Altman, 2014).

Evaluation of apoptotic phenotypes

To determine the phenotype produced by inducing cell-death during development in a specific set of cells defined by a given driver, crosses were set up using males from the driver in question

(either homozygous or combined with a genetically marked balancer chromosome) with balanced females of genotype *UAS-Stinger*, *UAS-hid* / *CyO*. Hid being a potent inducer of the intrinsic pathway of apoptosis (Sandu et al., 2010), this approach allowed the extent of apoptosis to be profiled in surviving non-balancer progeny by the absence of GFP expression in the target cells, and this correlated with any observed phenotype(s).

Lifespan measurements

Flies were mated for 2 days at room temperature. Eggs were collected into fresh vials and cultured to eclosion at 25 °C. Eclosed females were transferred to fresh vials (5 vials per group, 20 flies per vial) and tipped to new vials every 2-3 days until no live flies remained. At each transfer, the number of dead flies was recorded, from which a median lifespan for each vial was calculated.

Western blotting

Flies were anaesthetized and decapitated on ice and heads were snap-frozen for storage at -80 °C. Protein extracts were prepared from batches of 30 fly heads essentially as described by Fernandez-Ayala et al. (2009), by homogenization using a disposable plastic pestle, in PBS containing 1.5% (w/v) Triton X-100 plus, per 25 ml, one cOmplete™ EDTA-free Protease Inhibitor Cocktail tablet (Roche). After incubation at room temperature for 5 min, samples were centrifuged at 15,000 g_{max} for 10 min at room temperature, and supernatants transferred to fresh tubes on ice. Protein concentrations were determined using the Bradford assay. After the addition of an equal volume of SDS sample buffer (Laemmli 2× concentrate, Sigma-Aldrich), samples were heated for 5 min at 100 °C, briefly centrifuged to remove particulates, and 20 μg of each extract was loaded onto 18-well precast Any kD™ Criterion™ TGX Stain-Free™ Protein Gels (Bio-Rad), which were run and blotted as described (Andjelković et al., 2015). For detection of

ATP5A, α-actinin or GAPDH, blots were blocked in PBS-0.1% Tween (PBS-T) containing 5% nonfat milk for 2 h then reacted overnight in the same buffer at 4 °C with primary antibody (ATP5A: Abcam mouse monoclonal ab14748, 1:100,000; α-actinin: rabbit polyclonal C-20 sc-7454-R, Santa Cruz Biotechnology, 1:7,000); GAPDH: goat anti-GAPDH (C Terminus), EB06377, Everest Biotech, 1:5,000) in PBS-T containing 5% nonfat milk at 4 °C, and washed five times for 7 min in PBS-T. After incubation with secondary antibody (respectively, peroxidase-labeled horse anti-mouse IgG (H+L), Vector Laboratories PI-2000, 1:10,000; rabbit anti-goat IgG (H+L) HRP, Invitrogen, ThermoFisher Scientific 31402, 1:5,000 and peroxidase-labeled goat anti-rabbit IgG (H+L), Vector Laboratories PI-1000, 1:10,000) for 1 h at room temperature with further washes, blot signals were visualized by chemiluminescence (Immun Star® Luminol enhancer and substrate, Bio-Rad) and documented both by X-ray film as well as using a Bio-Rad ChemiDoc imager. For detection of Cox4 a similar procedure was used, except that the blocking agent was 5% BSA instead of nonfat milk, primary antibody was Abcam COXIV rabbit polyclonal, ab16056 (1:500) and secondary antibody was peroxidase-labeled goat anti-rabbit IgG (1:10,000; PI-1000; Vector Laboratories).

Tyrosine hydroxylase (TH) assay

The activity of tyrosine hydroxylase was measured spectrophotometrically by the formation of dopachrome, essentially as described earlier (Vermeer et al., 2013; Figueira et al., 2017). On the day of eclosion, flies were snap-frozen in liquid nitrogen, vortexed for 20 s at 4 °C to separate fly heads from bodies, then passed through a household sieve to collect the heads, which were stored at -80 °C unless processed immediately. Heads were homogenized (1 mg per 10 μl, using a plastic micropestle in a 1.5 ml Eppendorf tube) in ice-cold 50 mM Tris/HCl (pH 7.4) containing 0.1 mM phenylmethylsulfonyl fluoride (PMSF) and 0.1 mM dithiothreitol (DTT). TH activity was assayed in a reaction mixture containing 100 mM HEPES/KOH (pH 7.0), 0.25 mM

tetrahydrobiopterin, 500 μM ferrous sulphate, 200 μM tyrosine, 100 μM sodium periodate, and 20-40 μg of supernatant protein, with a blank without protein extract used for background subtraction. The reaction was monitored on a 2200 EnSpire plate reader (Perkin Elmer, US) at 475 nm for 30 min at 21 °C. Production of L-DOPA (L-3,4-dihydroxyphenylalanine) was determined using a molar extinction coefficient for dopachrome of $\varepsilon = 3700\ M^{-1}\ cm^{-1}$. One unit of tyrosine hydroxylase was defined as the amount of enzyme generating 1 μmol of L-DOPA per min. In figures TH activity is expressed in international milliunits (mU) per milligram of soluble protein (mU/mg protein). Protein content was measured by the Bradford (1976) method, with bovine serum albumin (1-8 μg) used as a standard.

Respirometry

Respirometry was conducted essentially as previously (Andjelković et al., 2015), except using batches of 50 freshly isolated heads from adult males instead of whole flies as source material, and employing the O2K oxygraph instrument (Oroboros).

TUNEL assay

Cell death was profiled essentially as described by Ghosh et al. (2011) and Trunova and Giniger (2012). Brains were dissected out in HL3.1 buffer (70 mM NaCl, 5 mM KCl, 20 mM $MgCl_2$, 10 mM $NaHCO_3$, 5 mM HEPES/NaOH, 115 mM sucrose, 5 mM trehalose, pH 7.2) and immediately fixed in freshly made (Yalgin et al., 2011) ice-cold 4% paraformaldehyde in 0.1% PBS (pH 7.2) for 20 min. After 3 washes for 20 min in 0.1% Triton X-100 in PBS (PBS-Tx), brains were permeabilized in 100 mM citrate in PBS-Tx at 65 °C for 30 min, then subjected to TUNEL reaction (In Situ Cell Death Detection Kit, fluorescein, Roche, Cat. No. 11684795910) for 3 h at 37 °C in the dark, followed by three washes for 20 min in PBS-Tx in the dark at room temperature. Brains were then immunostained for fluorescein according to the protocol below

(see also Table S2). Positive control brains were made by subjecting fixed wild-type brains to DNase I according to manufacturer's instructions (ThermoFisher Scientific, Cat. no. #EN0521) after the permeabilization step.

Immunohistochemistry

Brains were dissected out and fixed as for the TUNEL assay and blocked in freshly prepared 5% bovine serum albumin (BSA; Sigma #A7906) for 20 min at room temperature. They were then incubated in primary antibody solution (see Table S2), diluted in 5% BSA in PBS-Tx for ~36 hours at 4 °C, washed 3 times for 20 min in PBS-Tx, and incubated in secondary antibody solution, diluted in 5% BSA in PBS-Tx for 2 nights at 4 °C (Wu and Luo, 2006). After 3 final washes for 20 min in PBS-Tx, brains were mounted in 80% glycerol with coverslips raised by double-sided tape. Dissection of larvae for immunohistochemistry was as previously reported (Yalgin et al., 2011).

Image acquisition and analyses

Images from whole-mount brains were acquired confocally with a Zeiss LSM 700, and examined manually using Fiji ImageJ software (RRID: SCR_003070). Quantitation of TH signal in immunostained brains used images acquired with a Zeiss LSM 700 using a 10x objective. Brains with the same genotype were imaged under the same coverslip. Up to four brains were scanned at one time and then separated into separate TIFF files using ImageJ. Brightness was adjusted using the same settings for all samples in a given experiment. To assess signals quantitatively, the mean signal intensity of each cell body was measured from the image stack using the 'measure' function of ImageJ. The background signal intensity was separately measured, then subtracted from each ROI measurement. Measurements were statistically compared using

Microsoft Excel. Samples that had been mounted with the posterior surface facing the coverslip were not used in the analyses.

SUPPLEMENTAL REFERENCES

[In addition to references already cited in the main paper]

Bartholomew, N.R., Burdett, J.M., VandenBrooks, J.M., Quinlan, M.C., and Call, G.B. (2015). Impaired climbing and flight behaviour in *Drosophila melanogaster* following carbon dioxide anaesthesia. Sci. Rep. *5*, 15298.

Chou, Y., Spletter, M., Yaksi, E., Leong, J.C., Wilson, R.I., and Luo, L. (2010). Diversity and wiring variability of olfactory local interneurons in the *Drosophila* antennal lobe. Nat. Neurosci. *13*, 439–449.

Dietzl, G., Chen, D., Schnorrer, F., Su, K.C., Barinova, Y., Fellner, M., Gasser, B., Kinsey, K., Oppel. S., Scheiblauer, S., et al. (2007). A genome-wide transgenic RNAi library for conditional gene inactivation in *Drosophila*. Nature *448*, 151–156.

Fernandez-Ayala, D.J.M., Sanz, A., Vartiainen, S., Kemppainen, K., Babusiak, M., Mustalahti, E., Costa, R., Tuomela, T., Zeviani, M., Chung, J., et al. (2009). Expression of the *Ciona intestinalis* alternative oxidase (AOX) in *Drosophila* complements defects in mitochondrial oxidative phosphorylation. Cell Metab. *9*, 449–460.

Figueira, F.H., de Quadros Oliveira, N., de Aguiar, L.M., Escarrone, A.L., Primel, E.G., Barros, D.M., and da Rosa, C.E. (2017). Exposure to atrazine alters behaviour and disrupts the dopaminergic system in *Drosophila melanogaster*. Comp. Biochem. Physiol. C Toxicol. Pharmacol. *202*, 94–102.

Friggi-Grelin, F., Coulom, H., Meller, M., Gomez, D., Hirsh, J., and Birman, S. (2003). Targeted gene expression in *Drosophila* dopaminergic cells using regulatory sequences from tyrosine hydroxylase. J. Neurobiol. *54*, 618–627.

Ghosh, A., Manrique-Hoyos, N., Voigt, A., Schulz, J. B., Kreutzfeldt, M., Merkler, D., and Simons, M. (2011). Targeted ablation of oligodendrocytes triggers axonal damage. PloS One *6*, e22735.

Hummel, T., Krukkert, K., Roos, J., Davis, G., and Klämbt, C. (2000). Drosophila Futsch/22C10 is a MAP1B-like protein required for dendritic and axonal development. Neuron *26*, 357–370.

Kemppainen, E., George, J., Garipler. G., Tuomela, T., Kiviranta, E., Soga, T., Dunn, C.D., and Jacobs, H.T. (2016). Mitochondrial dysfunction plus high-sugar diet provokes a metabolic crisis that inhibits growth. PLoS One *11*, e0145836

Krzywinski, M., and Altman, N. (2014). Visualizing samples with box plots. Nat. Methods *11*, 119–120.

Kvon, E.Z., Kazmar, T., Stampfel, G., Yáñez-Cuna. J.O., Pagani, M,, Schernhuber, K., Dickson, B.J., Stark, A. (2014). Genome-scale functional characterization of *Drosophila* developmental enhancers in *vivo*. Nature *512*, 91–95.

Li, H., Chaney, S., Roberts, I.J., Forte, M., and Hirsh, J. (2000). Ectopic G-protein expression in dopamine and serotonin neurons blocks cocaine sensitization in *Drosophila melanogaster*. Curr. Biol. *10*, 211–214.

Mahr, A., and Aberle, H. (2006). The expression pattern of the *Drosophila* vesicular glutamate transporter: a marker protein for motoneurons and glutamatergic centers in the brain. 6, 299–309.

Nichols, C.D., Becnel, J., and Pandey, U.B. (2012). Methods to assay *Drosophila* behavior. J. Vis. Exp. (*61*), 3795.

Pfeiffer, B.D., Ngo, T.-T.B., Hibbard, K.L., Murphy, C., Jenett, A., Truman, J.W., and Rubin; G.M. (2010). Refinement of tools for targeted gene expression in *Drosophila*. Genetics *186*, 735–755.

Trunova, S., and Giniger, E. (2012). Absence of the Cdk5 activator p35 causes adult-onset neurodegeneration in the central brain of *Drosophila*. Dis. Model Mech. *5*, 210–219.

Vermeer, L.M., Higgins, C.A., Roman, D.L., Doorn, J.A. (2013). Real-time monitoring of tyrosine hydroxylase activity using a plate reader assay. Anal. Biochem. 432,11–15.

Wu, J. S., and Luo, L. (2006). A protocol for dissecting *Drosophila melanogaster* brains for live imaging or immunostaining. Nat. Protoc. *1*, 2110–2115.

Yalgin, C., Karim, M.R., Moore, A.W. (2011). Immunohistological labeling of microtubules in sensory neuron dendrites, tracheae, and muscles in the *Drosophila* larva body wall. J. Vis. Exp. (*57*), 3662.

SUPPLEMENTAL TABLES

Table S2. Antibodies used in immunohistochemistry, Related to Figures 1 and 4-9

**Antibody**	**Supplier**	**Dilution**	**RRID, Reference or Comment**
Rat anti-Elav, clone 7E8A10	Developmental Studies Hybridoma Bank	1:200	RRID:AB_528218
Mouse anti-Repo clone 8D12	Developmental Studies Hybridoma Bank	1:200	RRID:AB_528448
Rabbit anti-TH	Merck Millipore #AB152	1:200 or 500 (see figure legends)	RRID:AB_390204
Rabbit anti-GABA	Sigma #A2052	1:200 (1:500 in some experiments)	RRID:AB_477652
Mouse anti-ChAT, clone ChAT4B1	Developmental Studies Hybridoma Bank	1:100	RRID:AB_528122, Chou et al., 2010
Rabbit anti-fluorescein	Molecular Probes A-889	1:500	RRID:AB_221561
Rabbit anti-GFP	Abcam #ab6556	1:1000	RRID:AB_305564
Mouse anti-GFP, clone 1G9	Developmental Studies Hybridoma Bank	1:1000 (differs in some experiments: see figure legends)	RRID:AB_2617420
Rat anti-RFP, clone 5F8	Chromotek #5F8-100	1:200	RRID:AB_2336064
Mouse anti-actin, JLA-20-S	Developmental Studies Hybridoma Bank	1:200	RRID:AB_528068, for Westerns
Rat anti-CD8, clone 5H10	ThermoFisher Scientific MCD0800	1:200	RRID:AB_10392843

Rabbit anti-VGlut, affinity purified	kind gift of Dr H. Aberle	1:500	RRID:AB_2315544, Mahr and Aberle, 2006
Mouse anti-Futsch, clone 22C10	Developmental Studies Hybridoma Bank	1:1000	RRID:AB_528403, Hummel et al., 2000
Alexa 647 goat anti-rabbit	ThermoFisher Scientific A21245	1:500	RRID:AB_2535813
Alexa 488 goat anti-rabbit	ThermoFisher Scientific A11008	1:500	RRID:AB_143165
Alexa 647 goat anti-mouse	ThermoFisher Scientific A21240	1:500	RRID:AB_2535809
Alexa 488 goat anti-mouse	ThermoFisher Scientific A11001	1:500	RRID:AB_2534069
Alexa 594 goat anti-rabbit	ThermoFisher Scientific A11012	1:500	RRID:AB_2534079
Alexa 594 goat anti-mouse	ThermoFisher Scientific A11005	1:500	RRID:AB_2534073
Alexa 647 goat anti-rat	ThermoFisher Scientific A21247	1:500	RRID:AB_141778
Alexa 568 goat anti-rat	ThermoFisher Scientific A11077	1:500	RRID:AB_2534121
Alexa 568 goat ant-mouse	ThermoFisher Scientific A11004	1:500	RRID:AB_2534072
Alexa 568 goat anti-rabbit	ThermoFisher Scientific A11011	1:500	RRID:AB_143157
Alexa 488 goat anti-rat	ThermoFisher Scientific A11006	1:500	RRID:AB_2534074

LEGENDS TO SUPPLEMENTAL FIGURES

Figure S1. Supplemental data on effects of pan-neuronal COX7A knockdown (COX assembly, respirometry and apoptosis), Related to Figure 1

(A) Western blots of protein extracts (amounts loaded in the various gel tracks, as indicated, in μg) from pooled heads of 30 male flies of each genotype shown: con (control) – *elav-GAL4*^{C155}, *UAS-Dcr-2* / Y *; P{attP, y*$^{+}$*, w*^{3}} / +, and KD (COX7A knockdown) – *elav-GAL4*^{C155}, *UAS-Dcr-2* / Y *; UAS-RNAi*^{COX7A} / +. Horizontally separated tracks are from different gels, probed with antibodies as indicated in Transparent Methods; molecular weights (in kDa) of closest markers run on same gels, following Ponceau S staining of the membranes, as shown. The samples in (i) and (ii) represent two biological replicates. The extent of COX4 depletion was quantitated by densitometry and normalized against α-actinin as a loading reference, giving a residual signal of 34% of the control value for experiment (i) and 48% for experiment (ii). The latter value may be more reliable due to low signal intensities in (i). (B) Respirometry on extracts from pooled heads of 50 male flies of each genotype shown: con (control) and *elav*-GAL4>COX7A KD as above, normalized for protein content, using cI-, cIII- and cIV-linked substrate mixes. No error bars are shown since this was a single large-scale experiment using fresh material. Replicate experiments gave similar data. (C) TUNEL-staining of brain from *elav*-GAL4>COX7A KD male, prepared on the day of eclosion, alongside a *w*^{1118} positive control brain (DNase I-treated). TUNEL stain was amplified by immunohistochemistry (rabbit anti-fluorescein, followed by Alexa 488 goat anti-rabbit, see Table S2). Scale bar 50 μm.

Figure S2. Supplemental data on effects of pan-neuronal COX7A knockdown (apoptotic cells), Related to Figure 1

(A) Number of TUNEL-positive cells per brain (n = 10 for each class, as indicated). Boxplot

shows interquartile range (box), median (bold line), Tukey-style whiskers (Krzywinski and Altman, 2014) and outliers (open circles). *** denotes significant difference (Student's *t* test, $p < 0.001$). Note that this experiment was performed on 21-day old flies not carrying *UAS-Dcr-2*, i.e. the same control and knockdown genotypes as originally studied by Kemppainen et al. (2014) and by Andjelković et al. (2015), showing a milder knockdown phenotype than when *UAS-Dcr-2* is also present. (B) Immunocytochemistry for fluorescein (TUNEL) and nuclear markers for (i) glia (Repo) and (ii) neurons (Elav) in single optical sections from two elav-GAL4>COX7A KD knockdown brains (including *UAS-Dcr-2*). Scale bars 20 μm. Note that both classes of neural cells were affected, even though the driver itself is active only in neurons.

Figures S3 and S4. Supplemental data on effects of pan-neuronal COX7A knockdown (Apoliner), Related to Figure 1

Images of brains of elav-GAL4>COX7A KD males also expressing Apoliner (derived from the cross *UAS-RNAi*^{COX7A} / + ; *UAS-Apoliner* / *TM3, Sb* males to *elav-GAL4*^{C155} ; *UAS-Dcr-2* virgin females), prepared on the day of eclosion. Genotypes were verified by PCR on residual carcasses. GFP was detected by mouse anti-GFP primary antibody (1:2,000) and Alexa 488 goat anti-mouse secondary antibody (1:500), and Elav by rat anti-Elav (1:200) and Alexa 647 goat anti-rat secondary antibody (1:500). For full details of antibodies see Table S2. Images show single optical sections at 2 μm resolution. Fig. S3A (high) and Fig. S4 (low) magnification images, scale bars 20 μm and 100 μm, respectively. At highest magnification in Fig. S3B, one cell in the population appears to show clear co-localization of GFP with the Elav neuronal nuclear marker, although this may be an artifact due to low vertical resolution and high intensity of the cytoplasmic signal. Scale bar 5 μm.

Figure S5. Supplemental data on effects of pan-neuronal COX7A knockdown (ROS), Related to Figure 1

DHE staining of control and elav-GAL4>COX7A KD brains. Signal intensity on an arbitrary scale is as indicated. Scale bars 100 μm.

Figure S6. Counterstaining for Elav validates immunohistochemistry for TH, Related to Figure 1

To check that the depletion of TH observed in COX7A KD brains was not due to differential antibody penetration in different samples, brains of controls (genotype – *elav-GAL4*^{C155} / Y ; *UAS-Dcr-2* / *P{attP,y*$^{+}$*,w*$^{3'}$*}*) and elav-Gal4>COX7A KD flies (genotype – *elav-Gal4*^{C155} / Y; *UAS-Dcr-2* / *UAS-RNAi*^{COX7A}) were counterstained for Elav, a pan-neuronal nuclear marker, as well as TH (1:1,000), as indicated. Zoomed images (scale bars 20 μm; optimized for overall contrast and brightness but otherwise using same settings for controls and knockdown brains) show variable relative signal within a class, but generally lower TH signals in knockdown brains, as quantified in Fig. 1.

Figures S7, S8 and S9. Expression patterns produced by various neuronal GAL4 drivers (*TH*-, *Ddc*-, *TRH*-GAL4), Related to Figure 2

Immunohistochemistry for GFP and the indicated markers (TH – 1:200, Elav, Chat, GABA), of brains of flies expressing nuclear GFP (nGFP, 'Stinger') under the control of the indicated drivers, at different magnifications, as indicated by the scale bars. Fig. S7 – maximum intensity projection of (i, ii) brains of two flies expressing nGFP under the control of *TH*-GALl4. Scale bars 100 μm. Fig. S8 – maximum intensity projection of (i, ii) brains of two flies expressing nGFP under the control of *Ddc*-GAL4. Scale bars 100 μm. Fig. S9A – single optical section from such a specimen, at higher magnification. Scale bar 10 μm. Fig. S9B – maximum intensity

projection of brain of a fly expressing nGFP under the control of *TRH*-GAL4. Scale bar 100 µm.

Figure S10. Phenotypes produced by various neuronal GAL4 drivers, Related to Figure 2

(A) Locomotor impairment produced by COX7A knockdown using various drivers, as indicated, measured 7 or 10 days after eclosion (two separate experiments separated by dashed line). Box-plot nomenclature: boxes denote interquartile range (IQR), bold lines the median, with standard, Tukey-style whiskers (Krzywinski and Altman, 2014). (B) Median lifespan (mean $\pm$ SD, n = 5 except for controls, n = 4) of female flies in which COX7A was knocked down using the indicated drivers. Horizontal lines indicate significant differences (one-way ANOVA with Tukey *post hoc* HSD test, blue lines: $p < 0.01$, red lines: $p < 0.05$). Note that, based on these data, effects of COX7A knockdown in DA neurons were not simply delayed, compared with other neuronal classes. By 10 days of age (A), no effects on locomotor performance were seen and (B) lifespan was only slightly affected, using either of the dopaminergic or the serotonergic drivers.

Figures S11, S12 and S13. Expression pattern produced by the *Cha*-GAL4 driver, Related to Figure 2

Fig. S11A – low magnification images of a series of confocal planes, showing extensive overlap between expressed nGFP, driven by *Cha*-GAL4, and Elav, a pan-neuronal marker. Scale bar 100 µm. Fig. S11B – high magnification image (single optical section) showing the wide quantitative variation in GFP signal between neuronal nuclei, indicative of varying expression using the *Cha*-GAL4 driver. Intensity of staining arbitrarily indicated numerically as 1-5, in increasing intensity. Scale bar 5 µm. Fig. S11C – high magnification image (single optical section) showing that most nGFP-positive cells using the *Cha*-GAL4 driver are also positive for ChAT (choline acteyltransferase), but in many cases at very low levels. Scale bar 5 µm. Fig. S12 – maximum intensity projection of whole brain of a fly expressing nGFP under the control of *Cha*-Gal4.

Scale bar 100 μm. Fig. S13A – high magnification image (single optical section) confirming that some nGFP-positive cells using the *Cha*-GAL4 driver are also positive for TH. Scale bar 10 μm. Fig. S13B – high magnification image (single optical section) showing that some GFP-positive cells are also positive for GABA. Note that, in many cell bodies, the GABA antibody stains mainly the nucleus. Scale bar 20 μm. Thus, in line with Lucin et al. (2019), the *Cha*-GAL4 driver is not 100% specific for cholinergic neurons.

Figure S14. Expression patterns produced by the OK371 driver, Related to Figure 2

Maximum intensity projection of the brain from a fly expressing nGFP under the control of OK371, immunostained for GFP and Elav, as shown. Scale bar 100 μm.

Figures S15, S16 and S17. Expression driven by R55A05 is confined to cholinergic neurons, Related to Figures 3 and 4

Immunohistochemistry of brains from 5-day old male flies expressing various reporters under the control of the R55A05 GAL4 driver. Fig. S15 – maximum intensity projection of the whole brain stained for CD8 (genotype: *R55A05-GAL4* / *UAS-mCD8::GFP*). For a single optical section of an equivalent brain co-stained for CD8 and ChAT, see Fig. 3A. Scale bar 50 μm. Fig. S16A, S16B, S17A and S17B – maximum intensity projections of whole brains stained for (nuclear) GFP and the neural markers indicated: Fig. S16 – GABA, as a marker for GABAergic neurons, Fig. S17A – TH (1:200), as a marker for DA neurons, and Fig. S17B – Repo, as a marker for glial cells. Genotype: *UAS-Stinger* / + ; *R55A05-GAL4* / +. Scale bars: 100 μm, except Fig. S17B – 50 μm.

Figures S18, S19 and S20. R59E04 drives expression in a subset of cholinergic, but not DA neurons, Related to Figures 3 and 4

Fig. S18 – reference images showing expression patterns using R55A05 and R54E09 GAL4 drivers (Jenett et al., 2012), in the brain and ventral nerve cord as indicated – publicly available images generated by the Janelia FlyLight Project Team and the laboratories of Gerald M. Rubin, James W. Truman, Richard S. Mann, and Christopher Q. Doe, reproduced under Creative Commons Licence (CC BY 4.0). See also https://www.janelia.org/open-science/janelia-flylight-expression-patterns-gal4-and-lexa-driver-lines. Scale bars 100 µm, GFP and Bruchpilot (background control) signals as indicated. Fig. S19 – zoomed immunohistochemistry images from bottom panel of Fig. 4C: maximum projection image of three brain regions (i-iii) from a fly expressing (membrane-bound) mCD8-GFP under the control of the R59E04 GAL4 driver, co-stained for GFP and ChAT. Scale bars 20 µm. Fig. S20 – immunohistochemistry of brain from a 5-day old male fly expressing nuclear GFP under the control of the R59E04 GAL4 driver (genotype: *UAS-Stinger* / + ; *R59E04-GAL4* / +). Maximum intensity projection co-stained for GFP and for TH (1:200). Scale bar100 µm. Note that the discontinuity in the image in Fig. S20 is due to separate acquisition of the z-stacks using a relatively aged microscope that does not digitally smooth out such boundaries when stacks are combined.

Figure S21. R51C09 does not drive expression in GABAergic or DA neurons, Related to Figure 5

Immunohistochemistry of brains from 5-day old male flies expressing nuclear GFP under the control of the R51C09 GAL4 driver (genotype: *UAS-Stinger* / + ; *R51C09-GAL4* / +). Maximum intensity projection of the whole brain stained for GFP and for (A) GABA and (B) TH (1:500). Scale bars 100 µm. Note that the reporter used here (Stinger, nuclear GFP) enables colocalization to be visualized more reliably (green nuclei surrounded by magenta cytoplasm, rather than white

areas which could arise from signals in different cells not resolved by the optics). The GFP reporter used in Fig. 5 is the membrane bound (mCD8) version, which prominently labels projections in the optic lobe, whereas the nuclear GFP does not.

Figure S22. R52A01 does not drive expression in GABAergic or DA neurons, Related to Figure 6

Immunohistochemistry of brains from 5-day old male flies expressing nuclear GFP under the control of the R52A01 GAL4 driver (genotype: *UAS-Stinger* / + ; *R52A01-GAL4* / +). Maximum intensity projection of the whole brain stained for GFP and for (A) GABA and (B) TH (1:500). Scale bars 100 μm. Note that the reporter used here (Stinger, nuclear GFP) enables colocalization to be visualized more reliably (green nuclei surrounded by magenta cytoplasm, rather than white areas which could arise from signals in different cells not resolved by the optics). The GFP reporter used in Fig. 6 is the membrane bound (mCD8) version, which prominently labels projections in the optic lobe, whereas the nuclear GFP does not.

Figure S23. Minimal overlap of expression directed by cholinergic drivers R55A05 and R59E04, Related to Figures 4-6

(A) Maximum intensity projection, (B) zoomed substack from (A) and (C, D) single optical sections from brain from a female fly co-expressing GFP (green) under the control of driver R59E04 and RFP (magenta) under the control of R55A05, using the LexA and GAL4 expression systems, respectively. Genotype was *10XUAS-IVS-mCD8::RFP*, *13XLexAop2-mCD8::GFP* / + ; *R59E04-lexA* / + ; *R55A05-GAL4* /+. Scale bars (A) 100 μm, (B, C, D) 20 μm. Note the almost total lack of overlap, even for brain regions where neurites targeted by each driver intermingle, as in (B). In a small number of specific brain regions, e.g., in (C), there is overlap of the signals, whereas in most of the brain, e.g., in (D), they are completely separate.

Figure S24. Minimal overlap of expression directed by glutamatergic drivers R52A01 and R51C09, Related to Figures 4-6

(A) Maximum intensity projection, (B) single optical section and (C) zoomed area from (B) of brain from a female fly co-expressing GFP (green) under the control of driver R52A01 and RFP (magenta) under the control of R51C09, using the LexA and GAL4 expression systems, respectively. Genotype was *10XUAS-IVS-mCD8::RFP*, *13XLexAop2-mCD8::GFP* / + ; *R52A01-lexA* / + ; *R51C09-GAL4* /+. Scale bars (A, B) 100 µm, (C) 20 µm. Note specific brain structures in (C), where both reporters are expressed, indicating a small degree of overlap between the drivers. See also images from the FlyLight collection at: http://flweb.janelia.org/cgi-bin/view_flew_imagery.cgi?line=R51C09 and http://flweb.janelia.org/cgi-bin/view_flew_imagery.cgi?line=R52A01.

Figures S25 through S31. Brain regions where DA neurons and R55A05 (or R59E04) neurons project show a small degree of overlap, Related to Figure 7

Fig. S25 and S26 – immunohistochemistry for TH (1:200, green) and CD8 (magenta) of brains from male fly expressing mCD8-Cherry under the control of Fig. S25 – R55A05 and Fig. S26 – R59E04. Genotypes – *R55A05-GAL4* (or *R59E04-GAL4*) / *UAS-mCD8::Cherry*. Maximum projection images of the whole brain (scale bar 100 µm), with zoomed areas as indicated. Fig. S27, S28 and S29 – individual TH and RFP signals from optical sections of the brain analyzed in Fig. 7, showing minimal overlap between (TH-positive) DA neurons and neurons post-synaptically expressing RFP under the control of trans-Tango, driven by R55A05. Scale bars 10 µm. Panel numbers correspond with those of Fig. 7. The clusters of neurons intensely stained for TH (panels *ii*, *iii*, and *v*) are negative for RFP, whilst the cell-bodies of a few neurons staining intensely for RFP are essentially negative for TH (panel *vii*). A few neurites are stained for both

markers, as shown by the small minority of signals that overlap (panels *x* and *xii*). Fig. S30 and S31 – optical sections of the brain analyzed in Fig. 7, showing the location of the DA neuron clusters visualized in panels *i*-*v* thereof. Scale bars in zoomed images 10 µm.

Figures S32 through S39. TH deficiency in subsets of neurons with COX7A knockdown, Related to Figures 8 and 9

Immunohistochemistry for TH (1:1,000) and Elav in brains from flies with COX7A knockdown or controls with only Dcr-2 overexpression, as indicated, using the GAL4 drivers shown (maximum intensity projections). Individual specimens also shown in Fig. 8 or 9 (TH only) indicated by *. Genotypes – Dcr-2 only: + ; *R55A05-GAL4* (or *R59E04-GAL4*, or *R52A01-GAL4* or *R51C09-GAL4*) / *UAS-Dcr-2*, COX7A-KD: *UAS-RNAi*^{COX7A} / + ; *R55A05-GAL4* (or *R59E04-GAL4*, or *R52A01-GAL4* or *R51C09-GAL4*) / *UAS-Dcr-2*. Scale bars 100 µm. Contrast and brightness have been similarly adjusted in each image, giving uniform background fluorescence. Sample variation and the subtle nature of the observed differences preclude reliable quantitation.

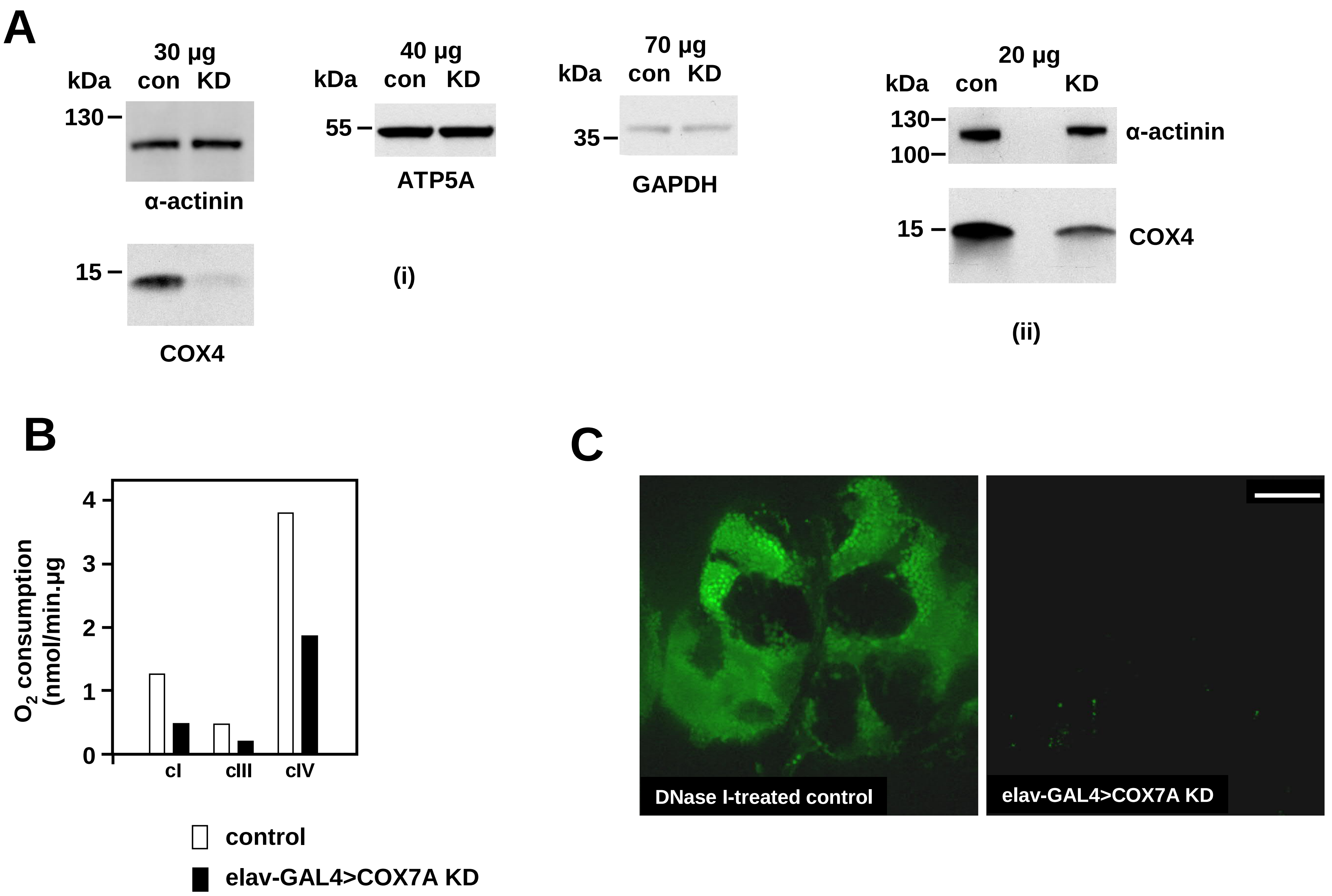

Yalgin et al, Figure S1

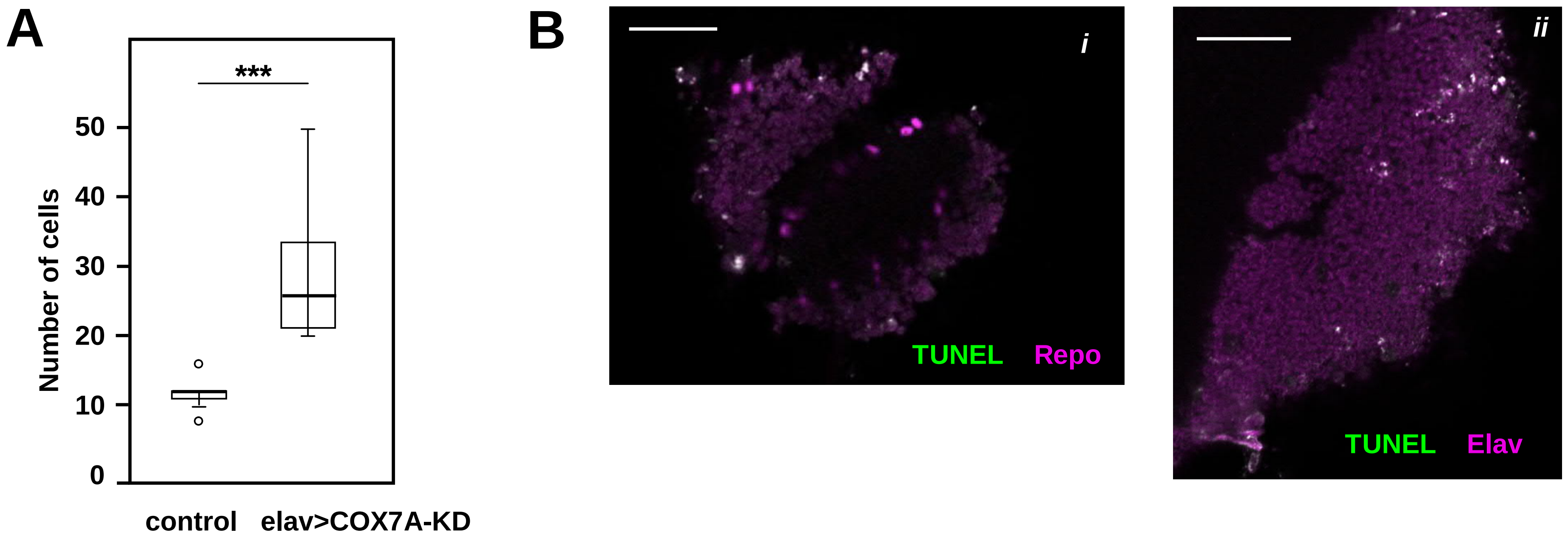

Yalgin et al, Figure S2

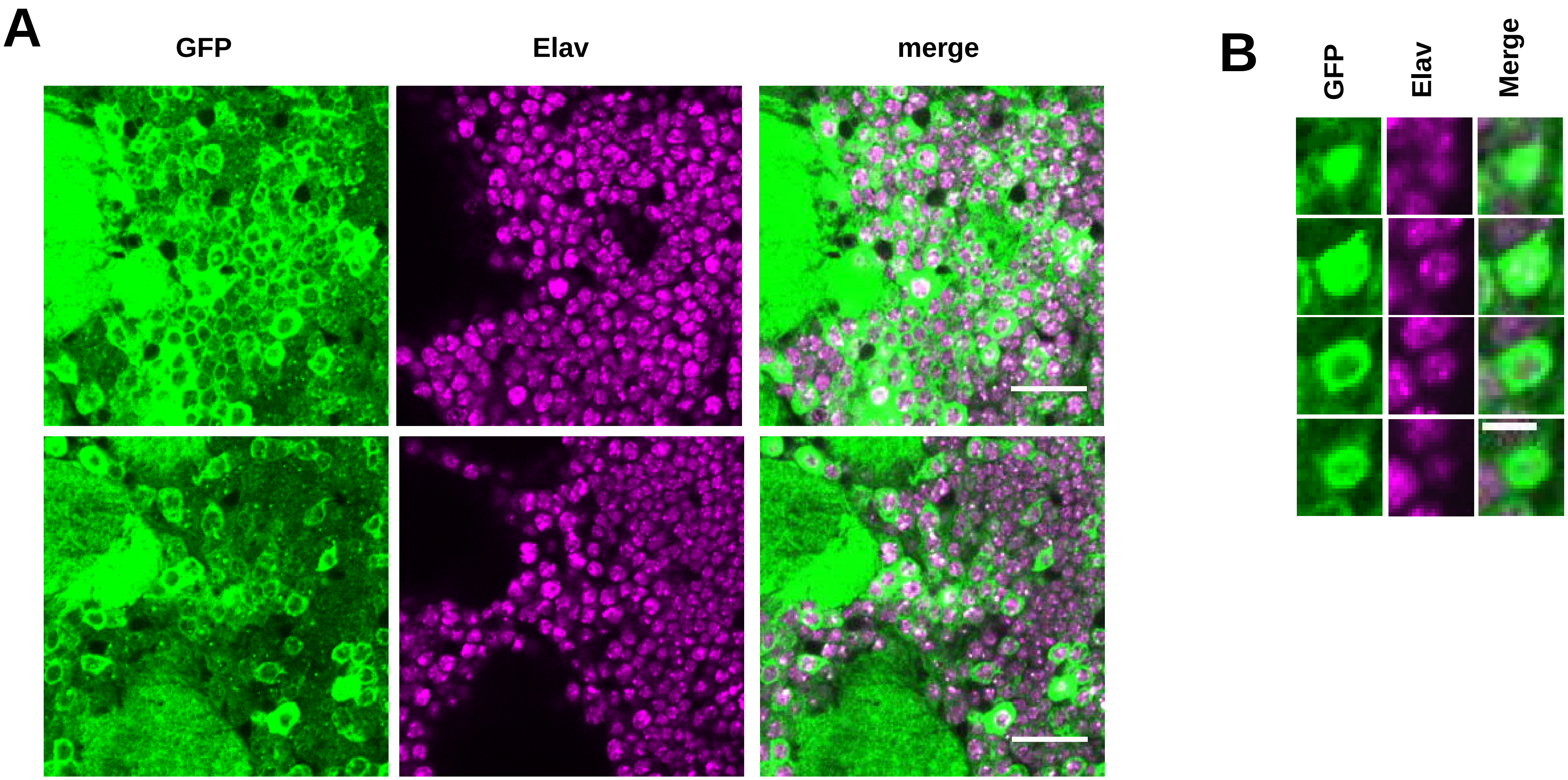

Yalgin et al, Figure S3

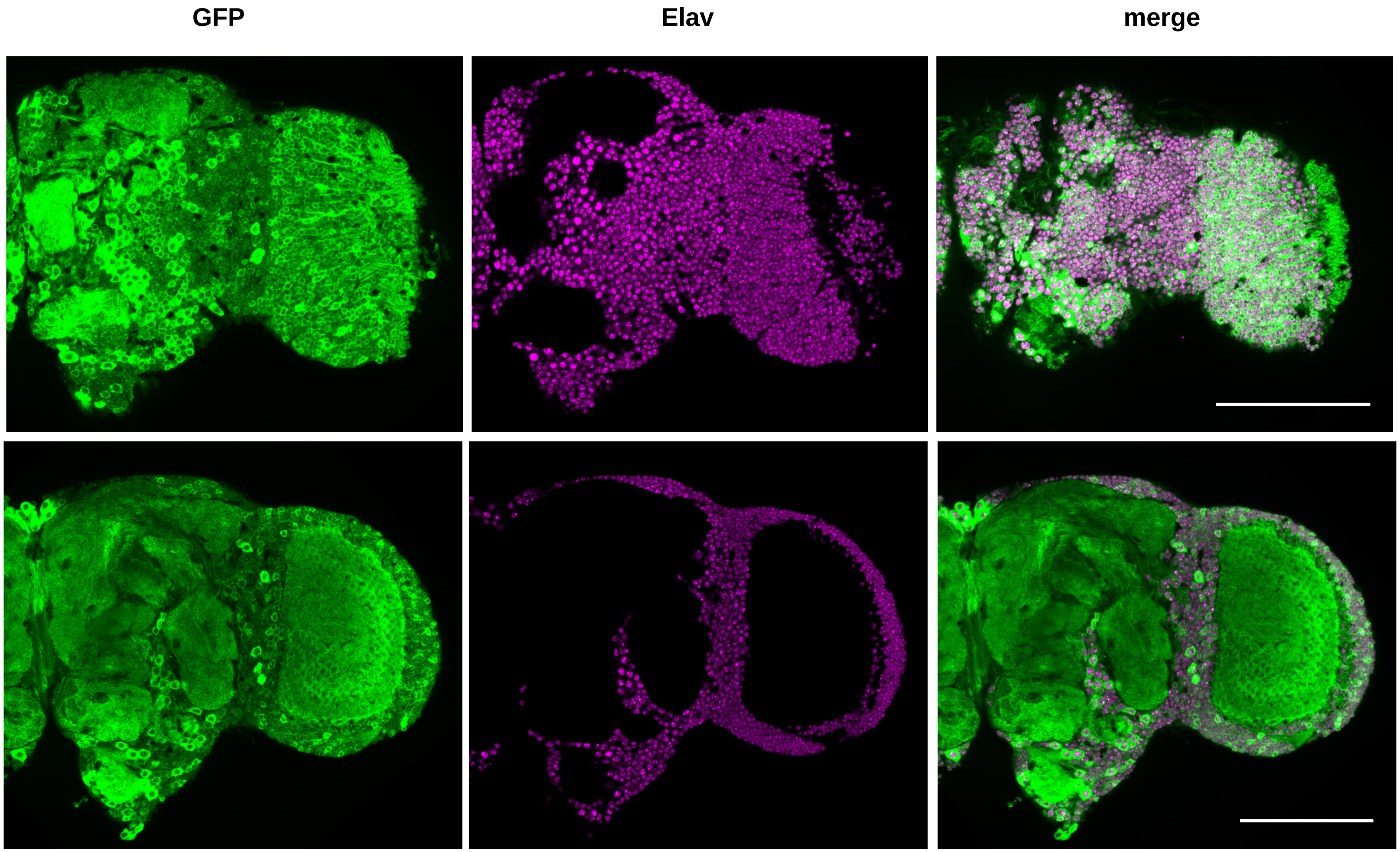

Yalgin et al, Figure S4

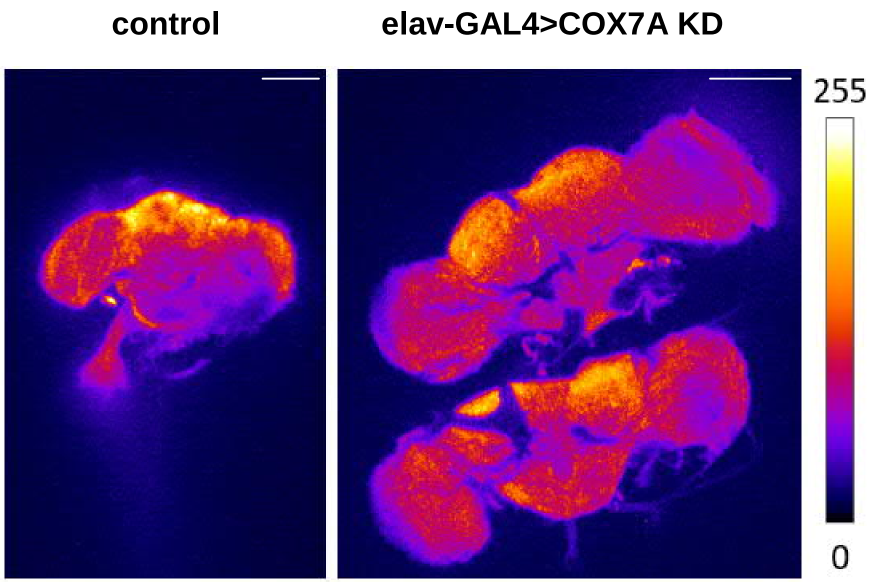

Yalgin et al, Figure S5

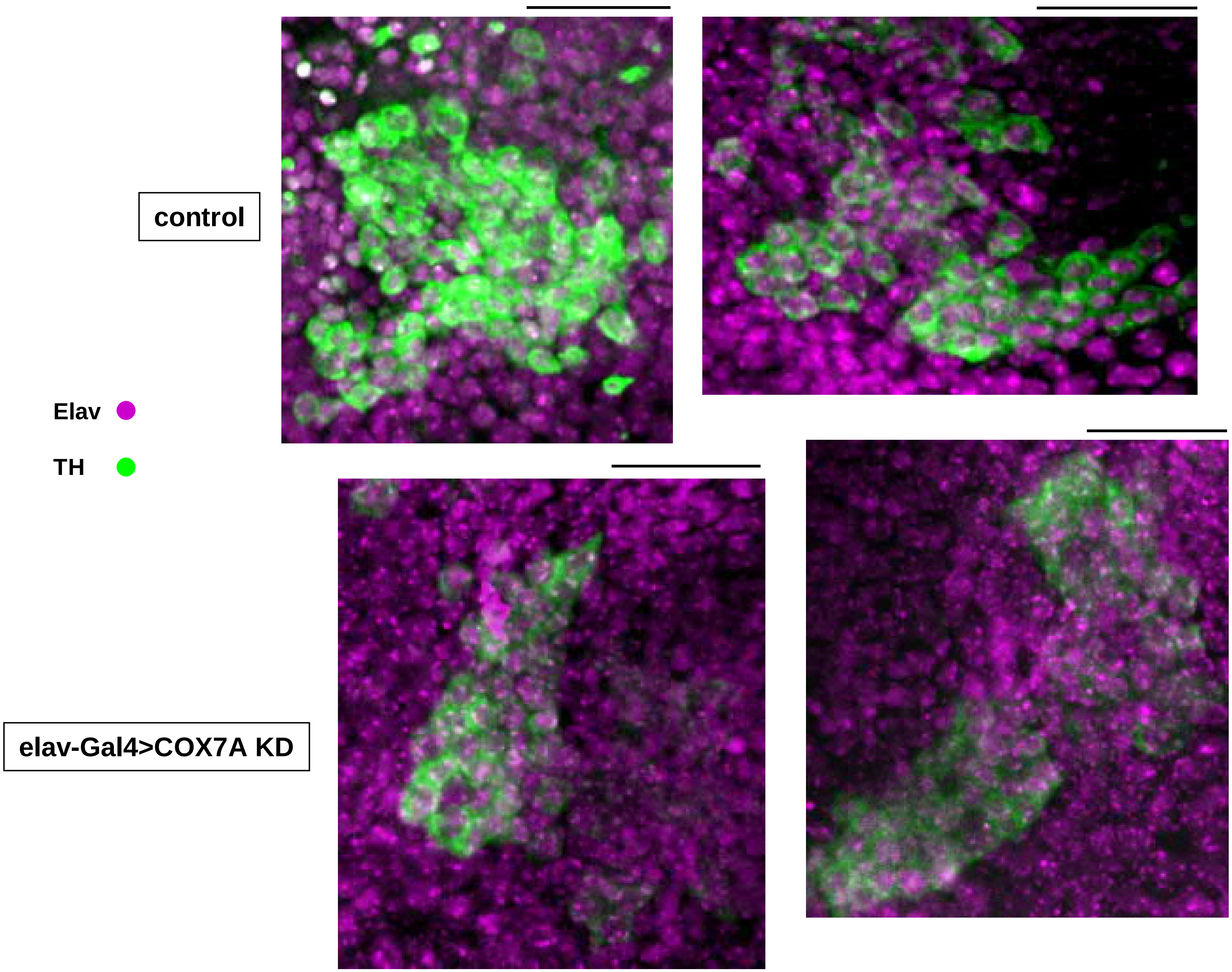

Yalgin et al, Figure S6

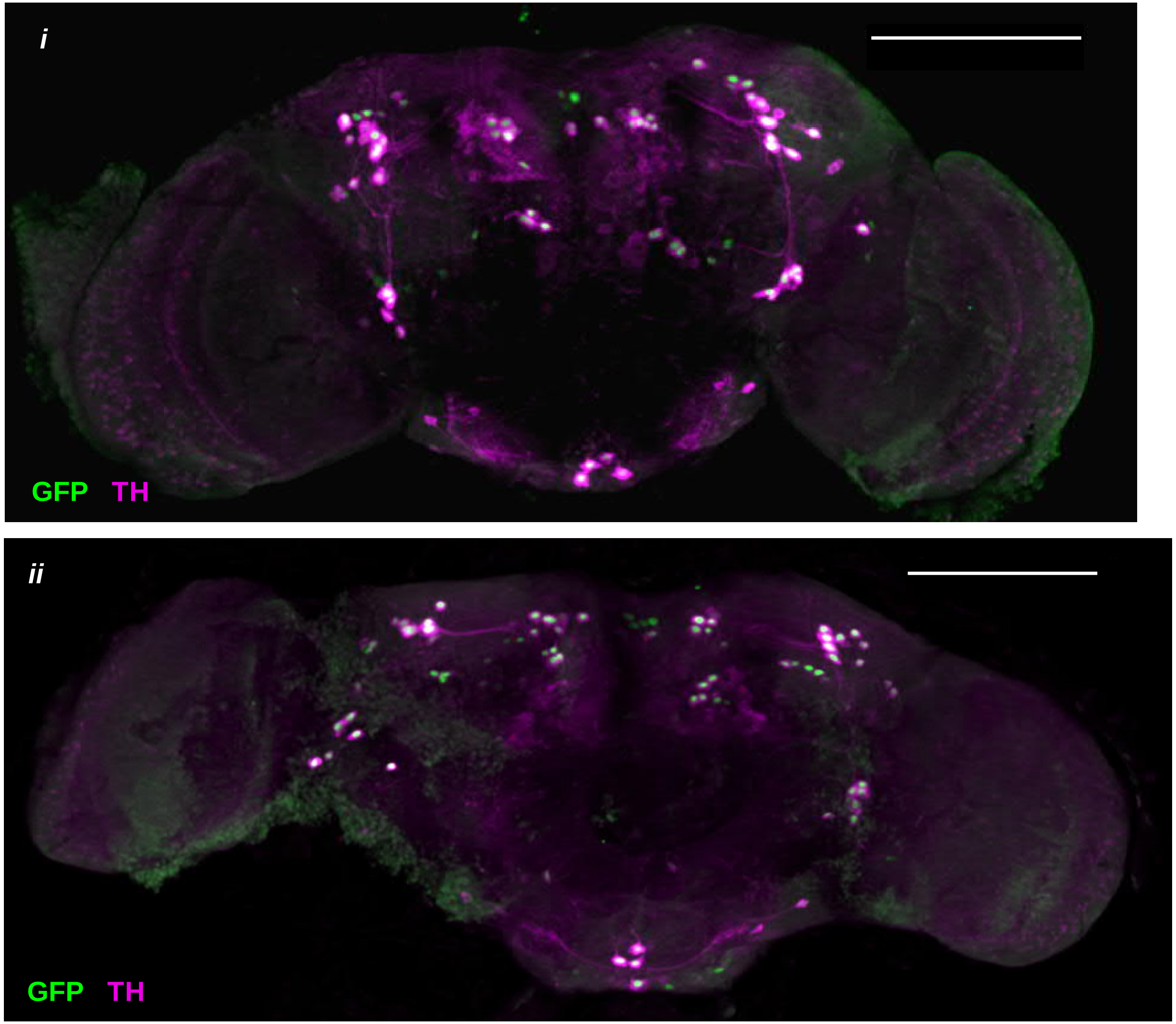

TH-GAL4>nGFP

Yalgin et al, Figure S7

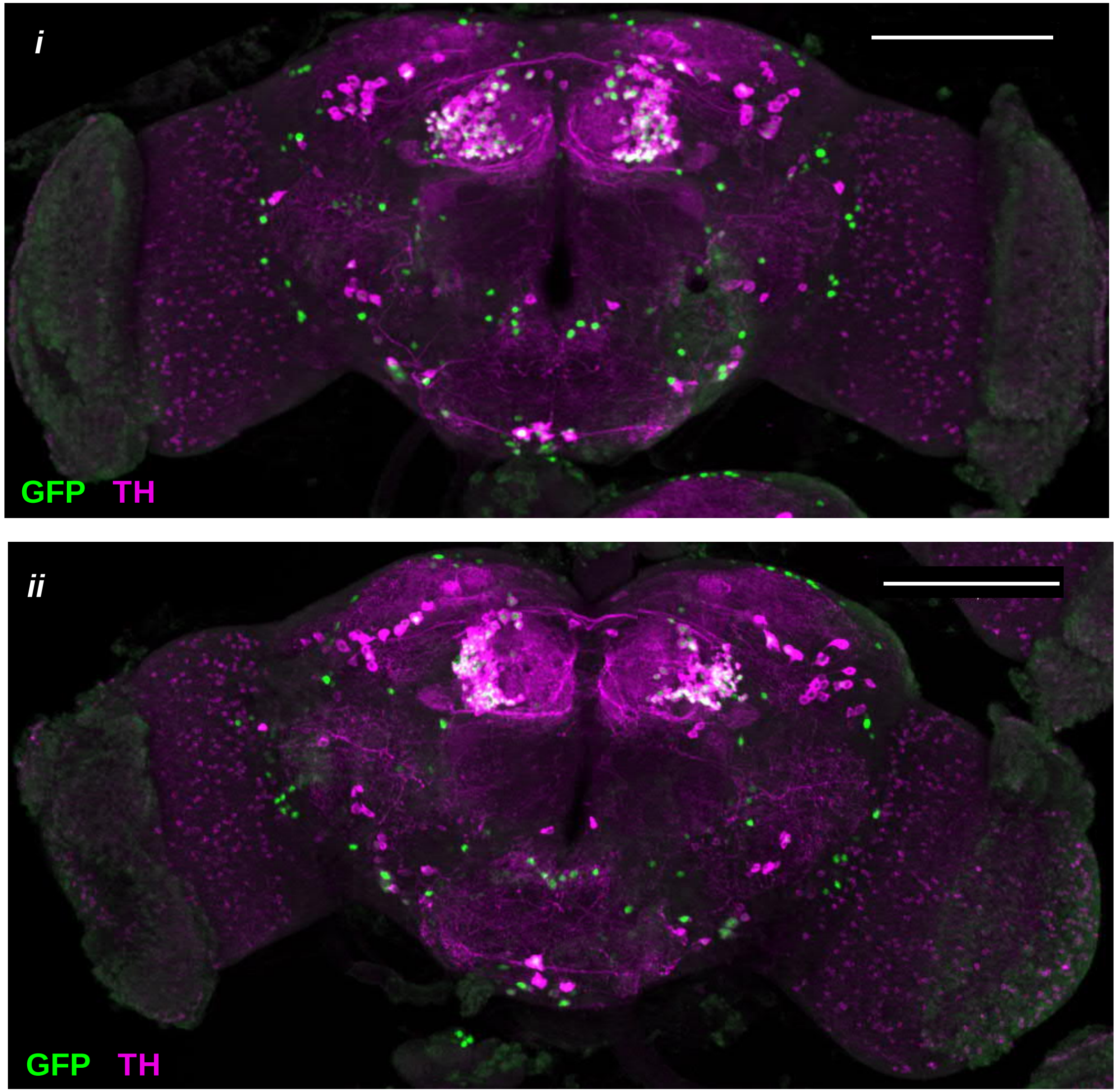

Ddc-GAL4>nGFP

Yalgin et al, Figure S8

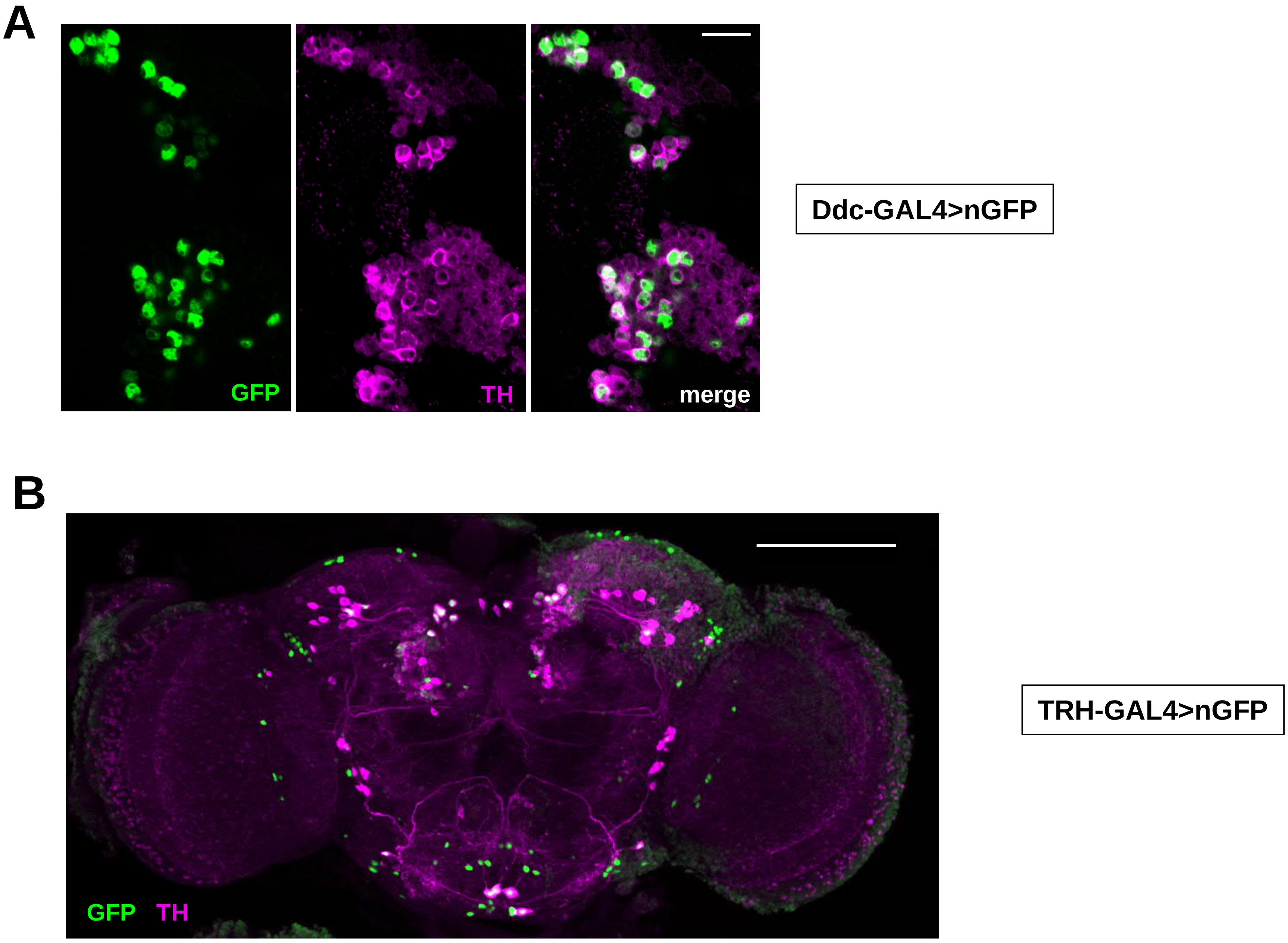

Yalgin et al, Figure S9

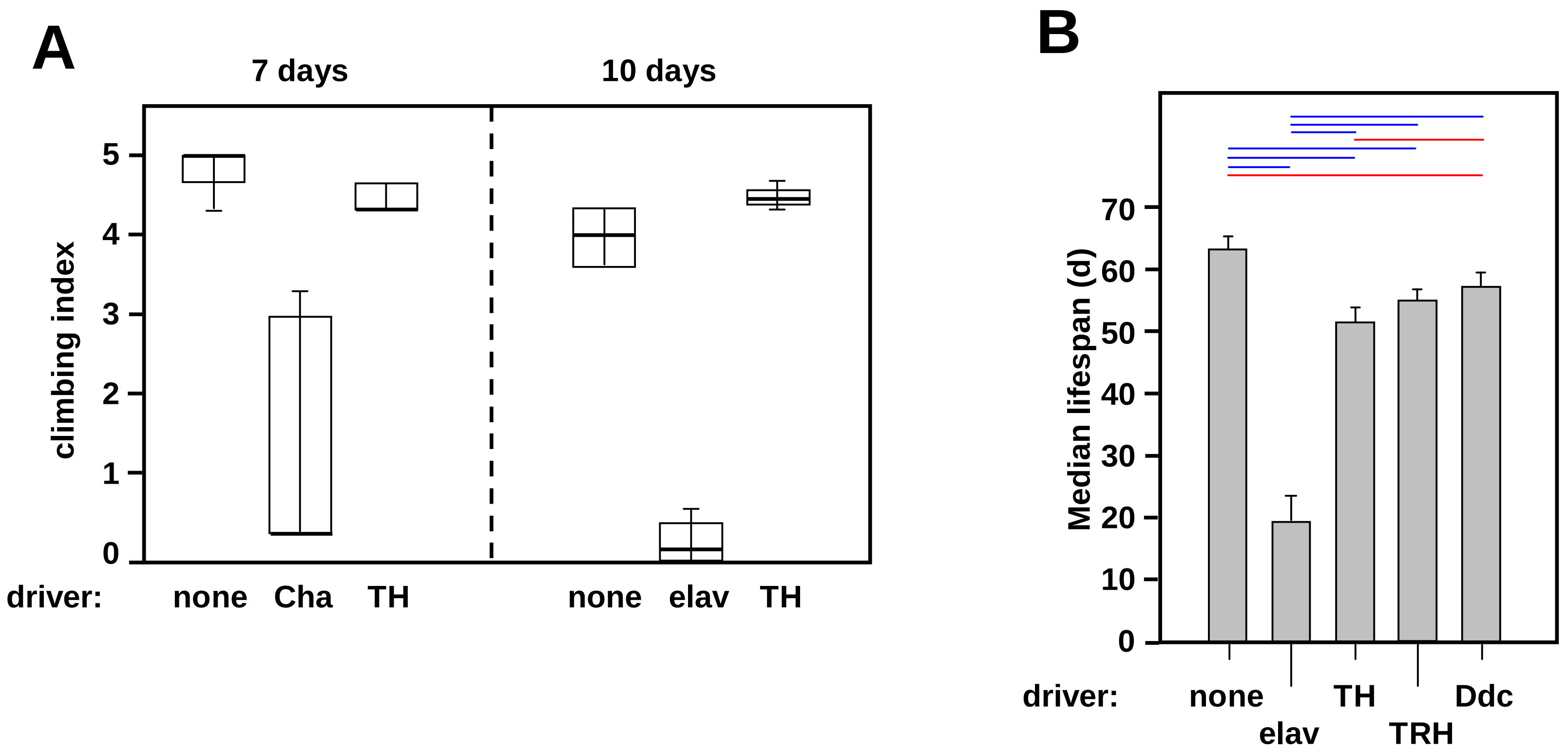

Yalgin et al, Figure S10

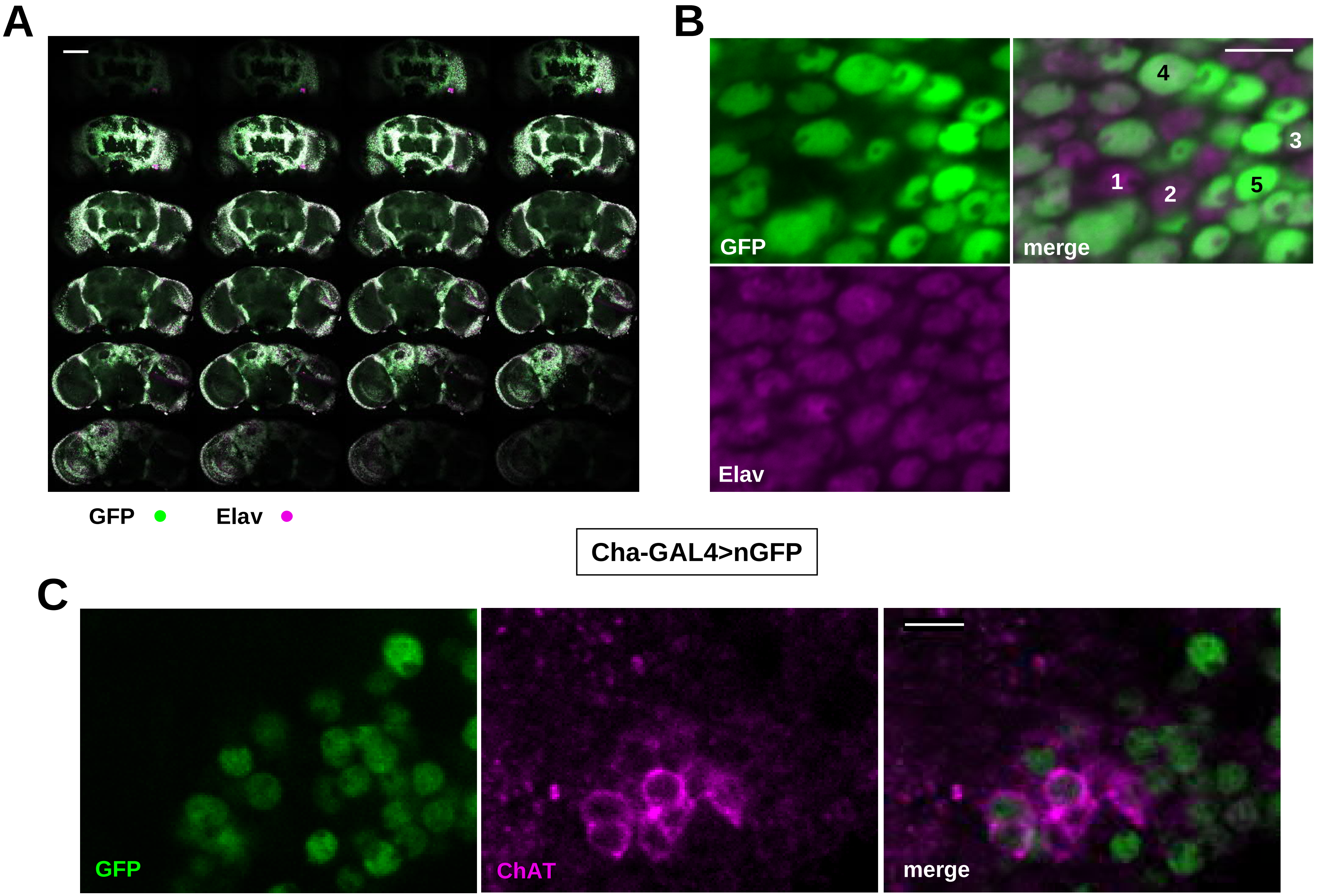

Yalgin et al, Figure S11

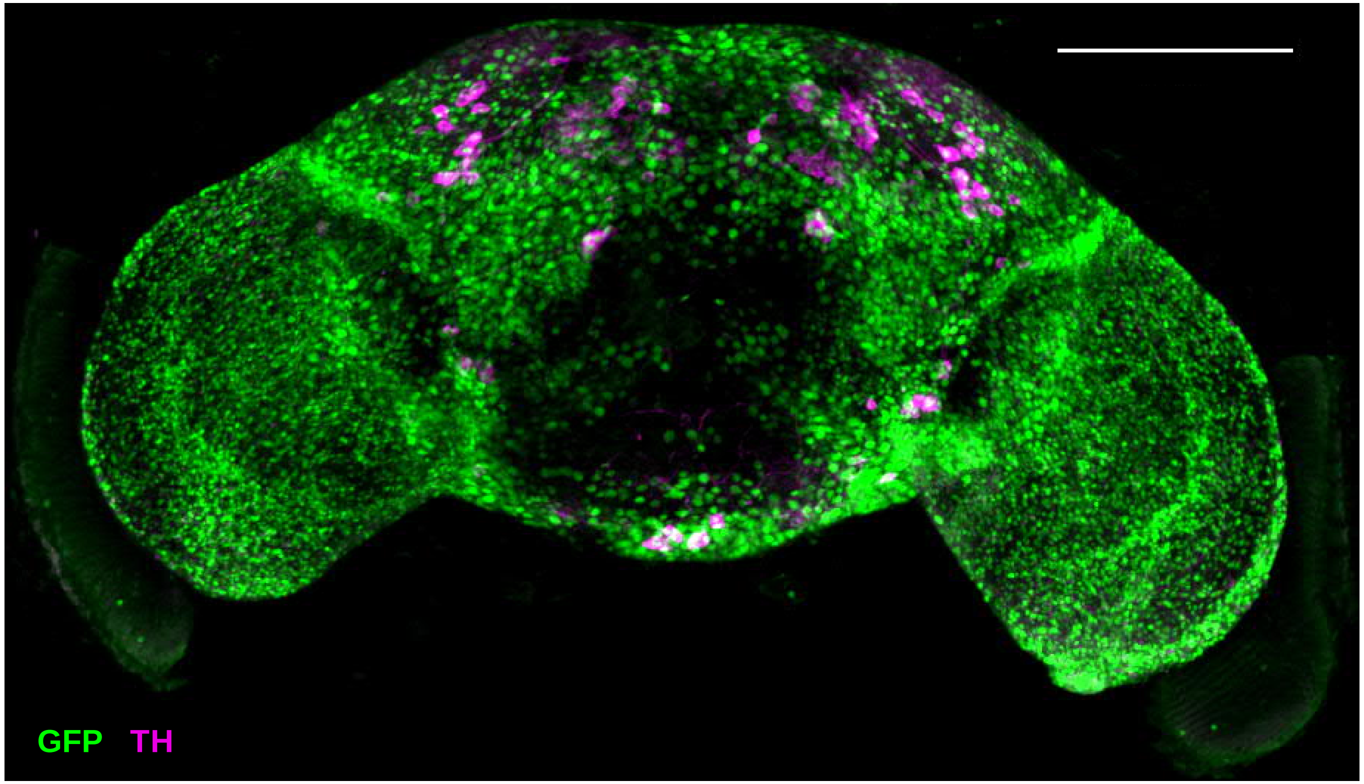

Cha-GAL4>nGFP

Yalgin et al, Figure S12

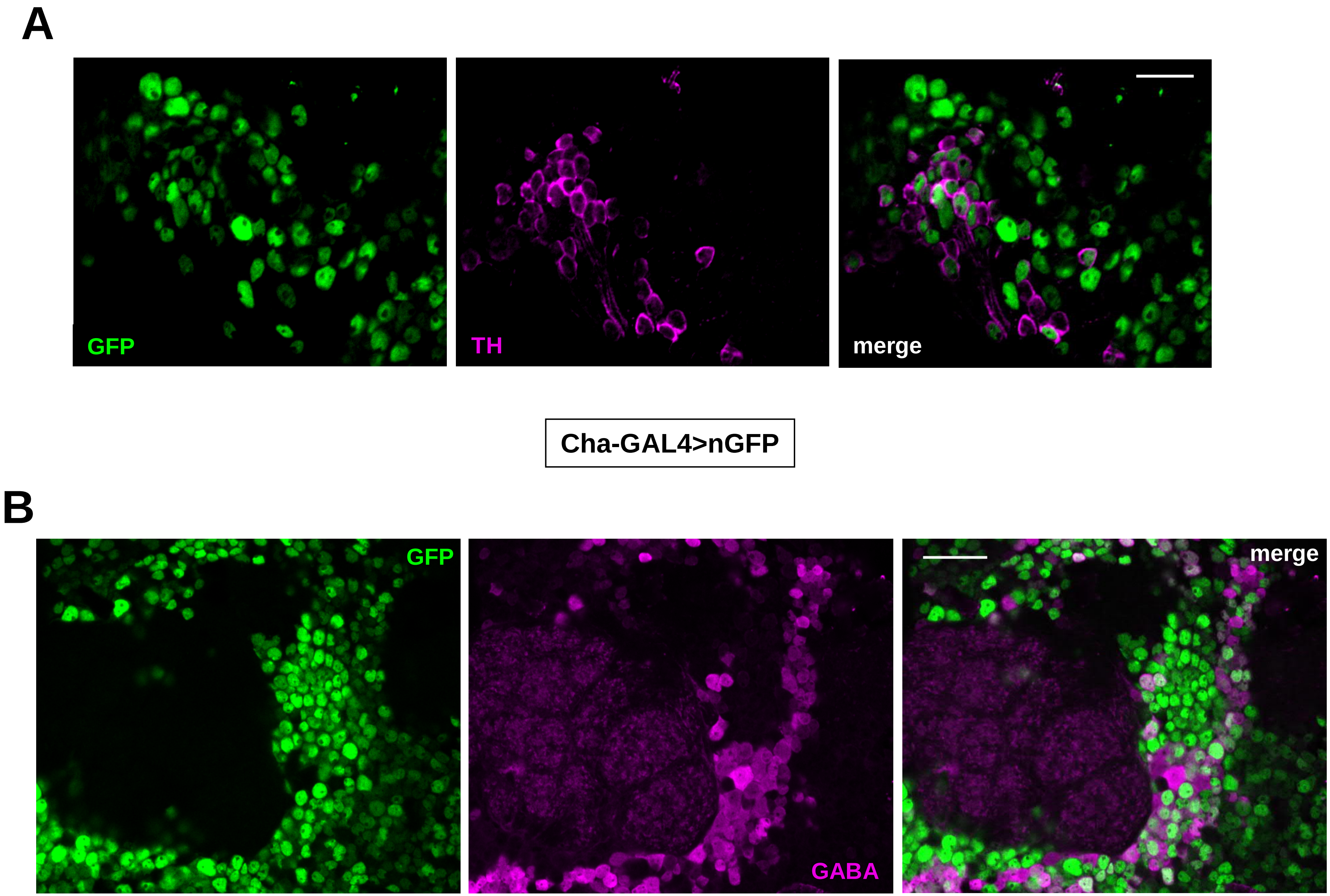

Yalgin et al, Figure S13

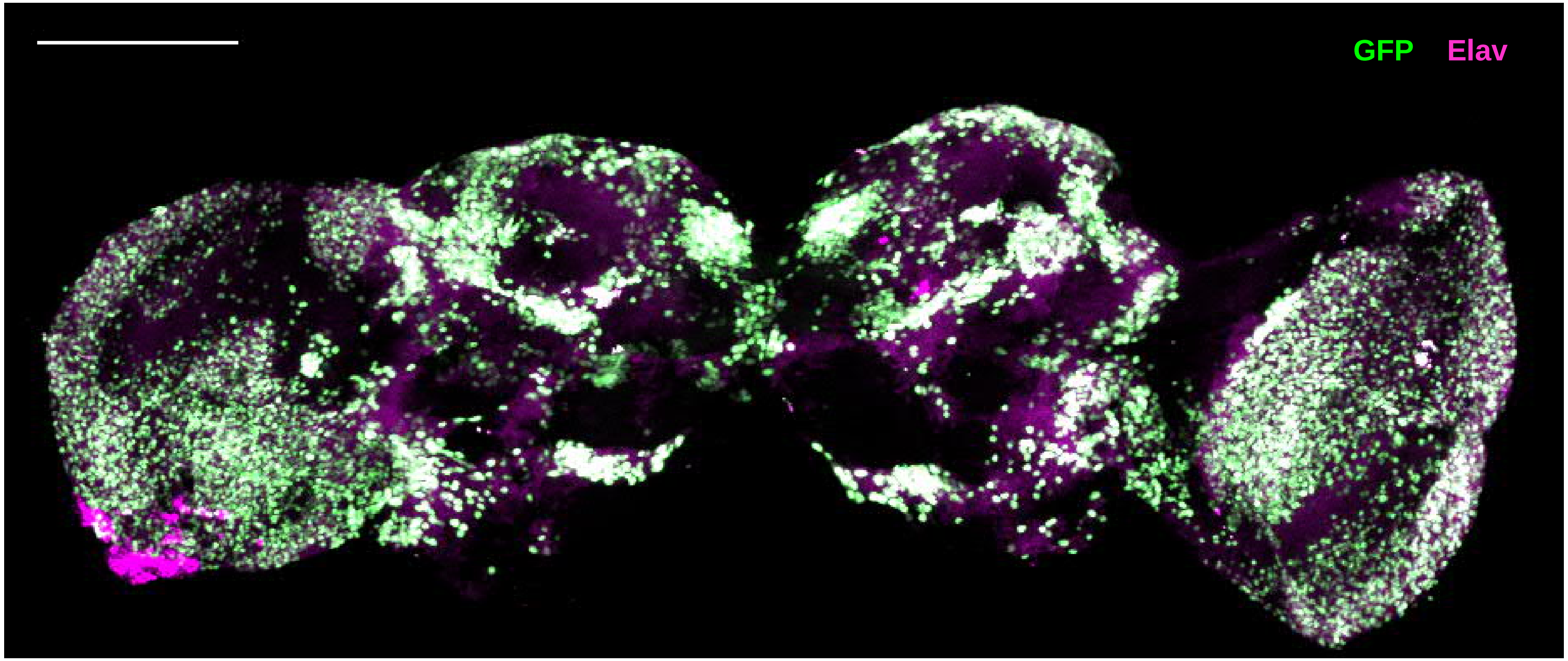

OK371>nGFP

Yalgin et al, Figure S14

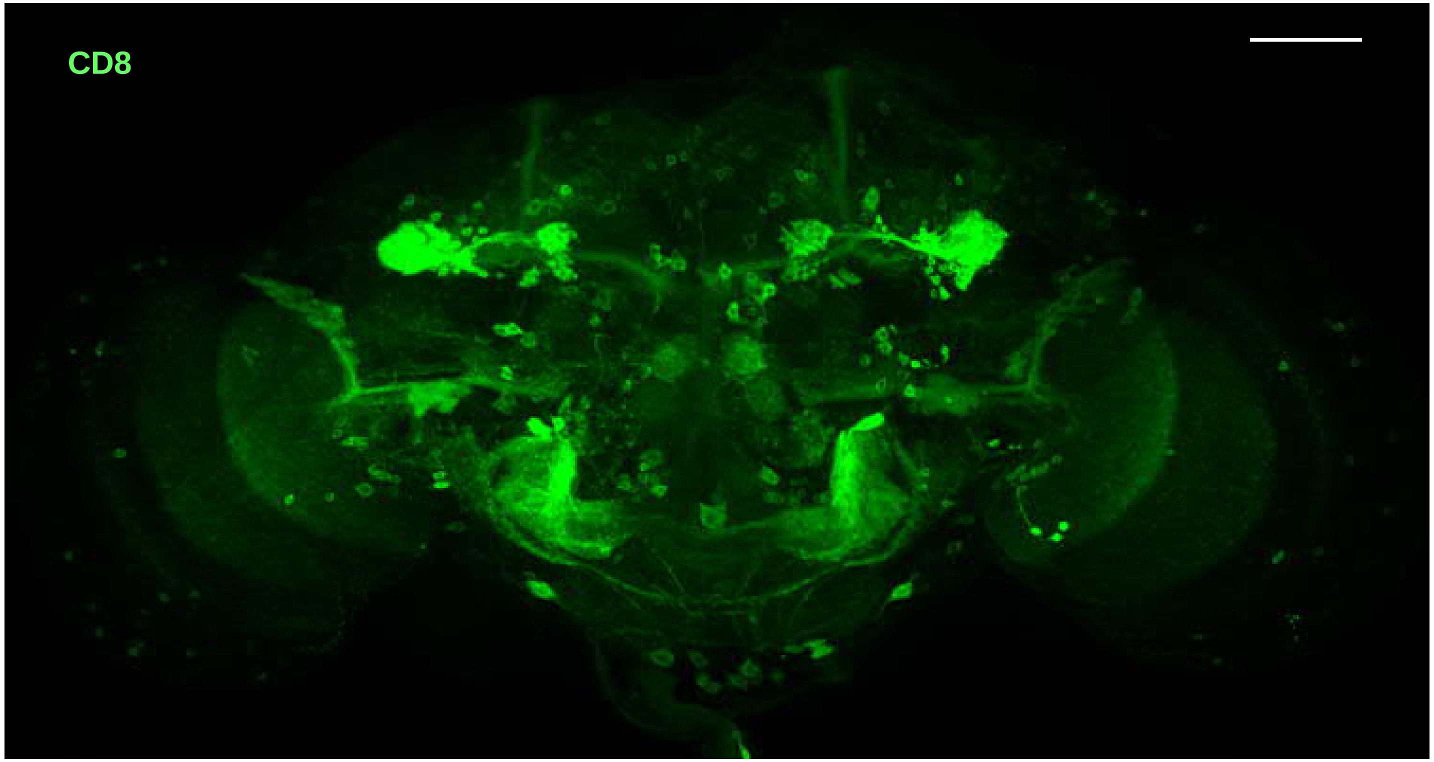

R55A05>mCD8-Cherry

Yalgin et al, Figure S15

A

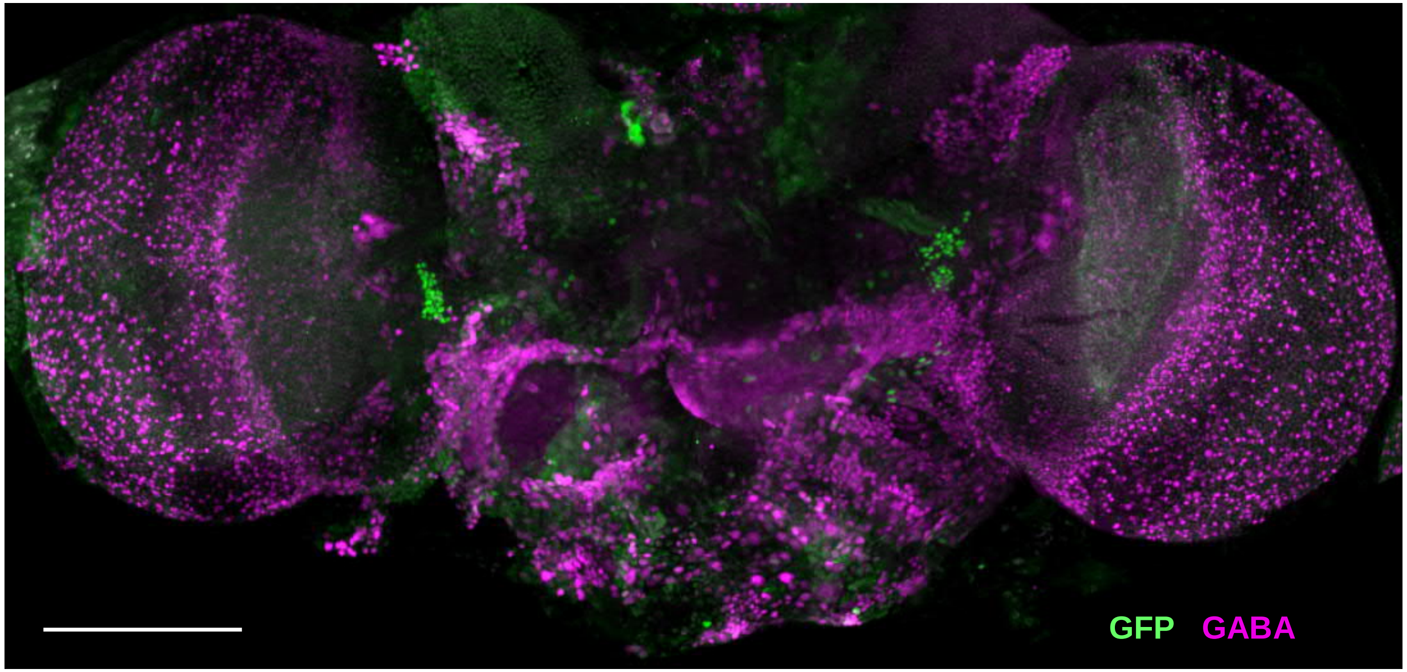

B

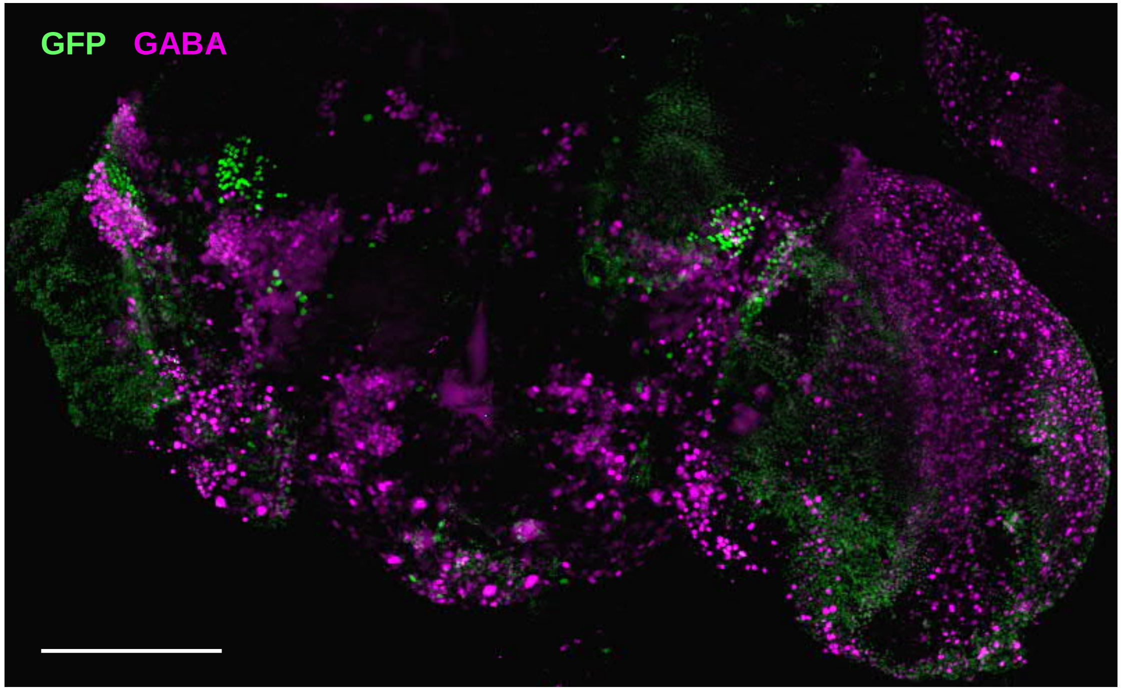

R55A05>nGFP

Yalgin et al, Figure S16

A

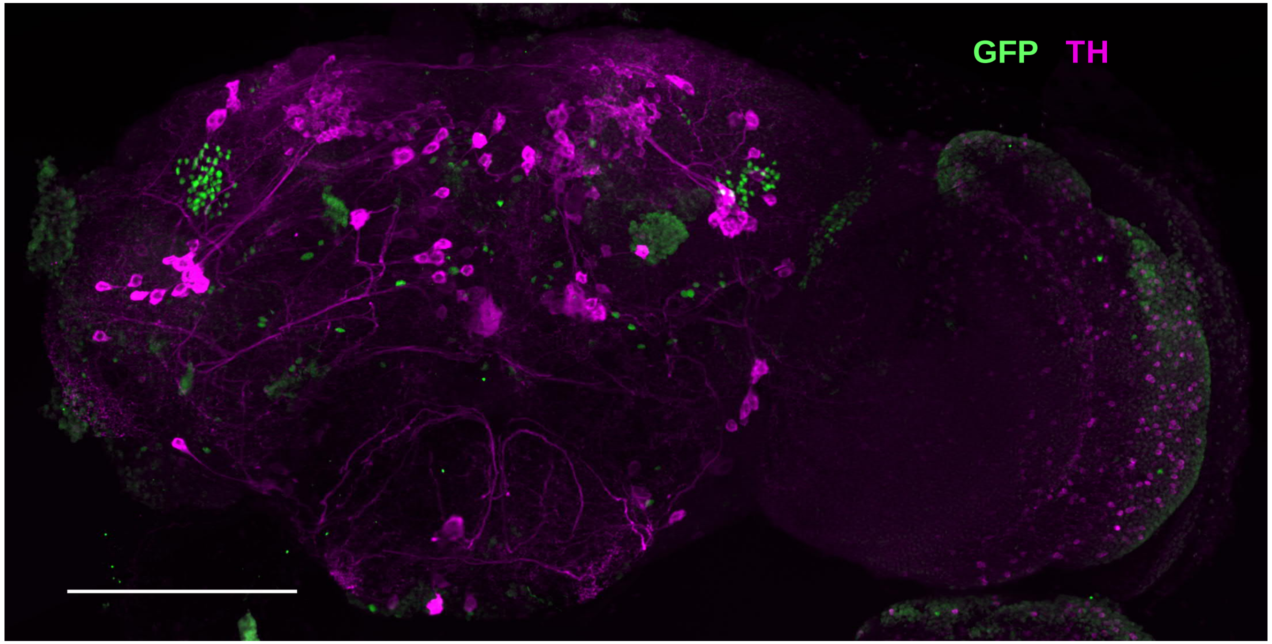

R55A05>nGFP

B

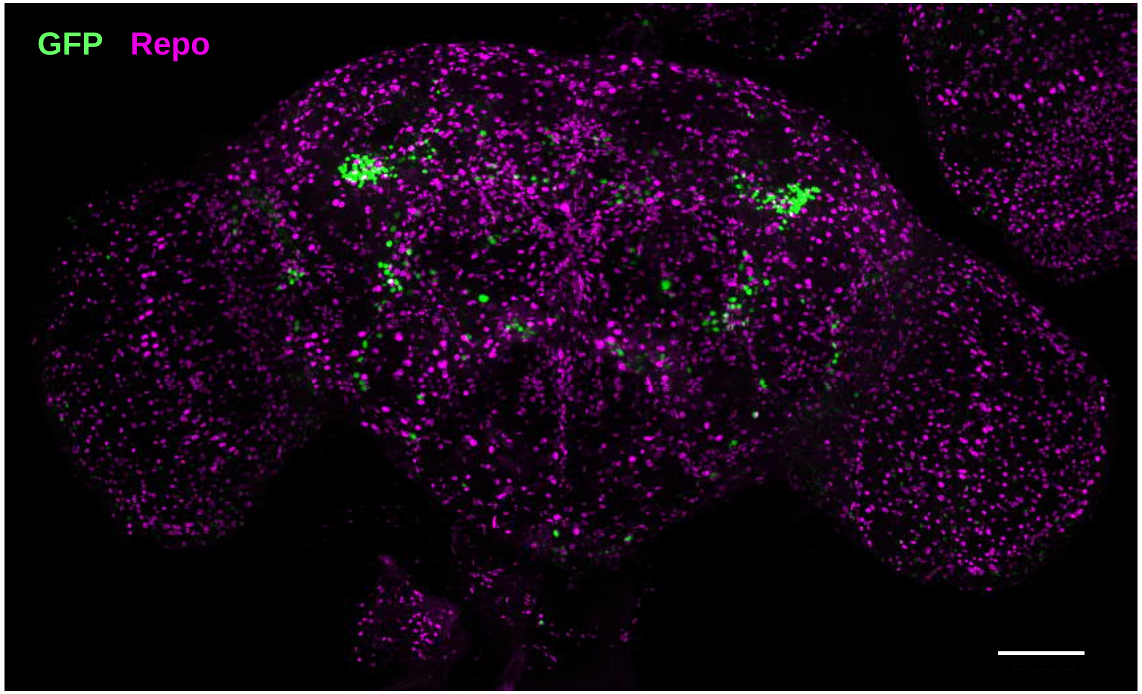

Yalgin et al, Figure S17

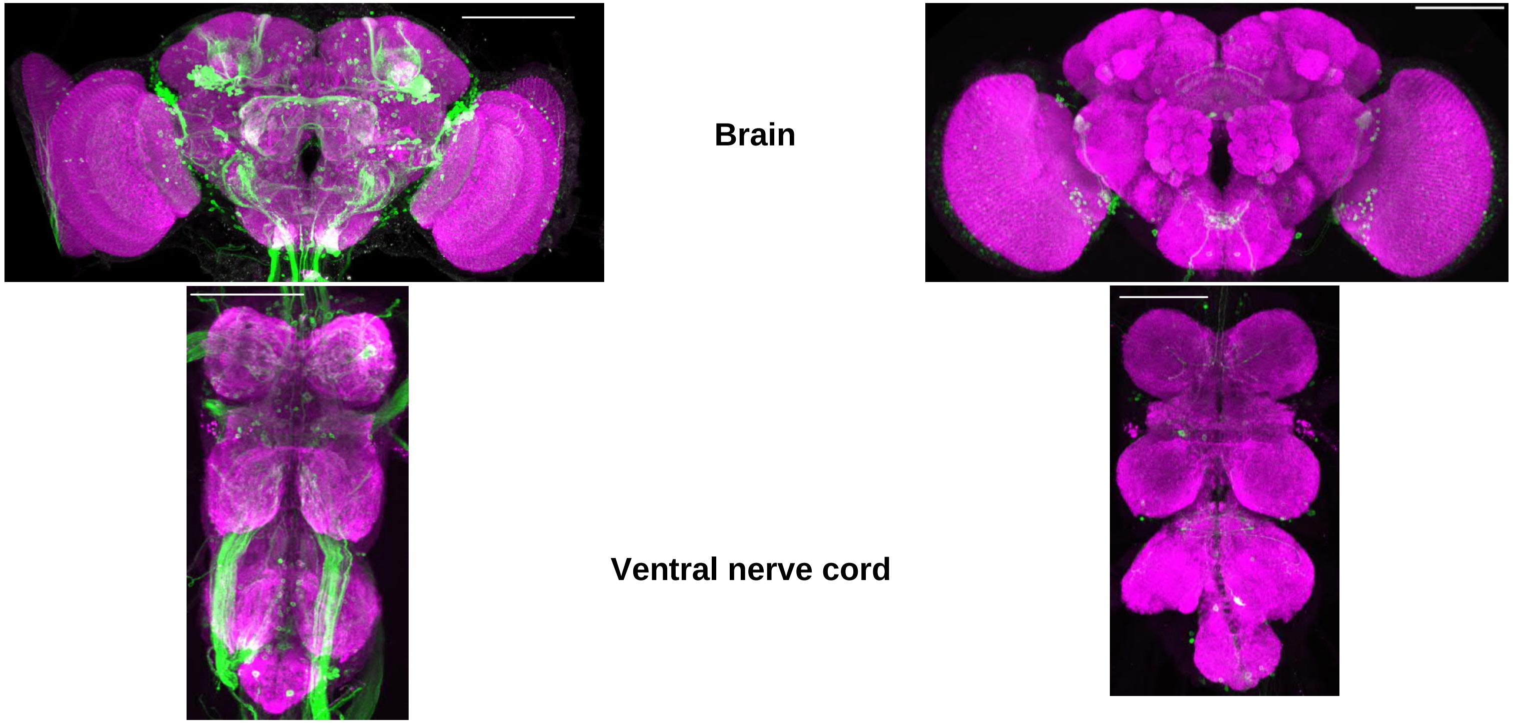

Images reproduced from publicly available Janelia Flylight website: see legend

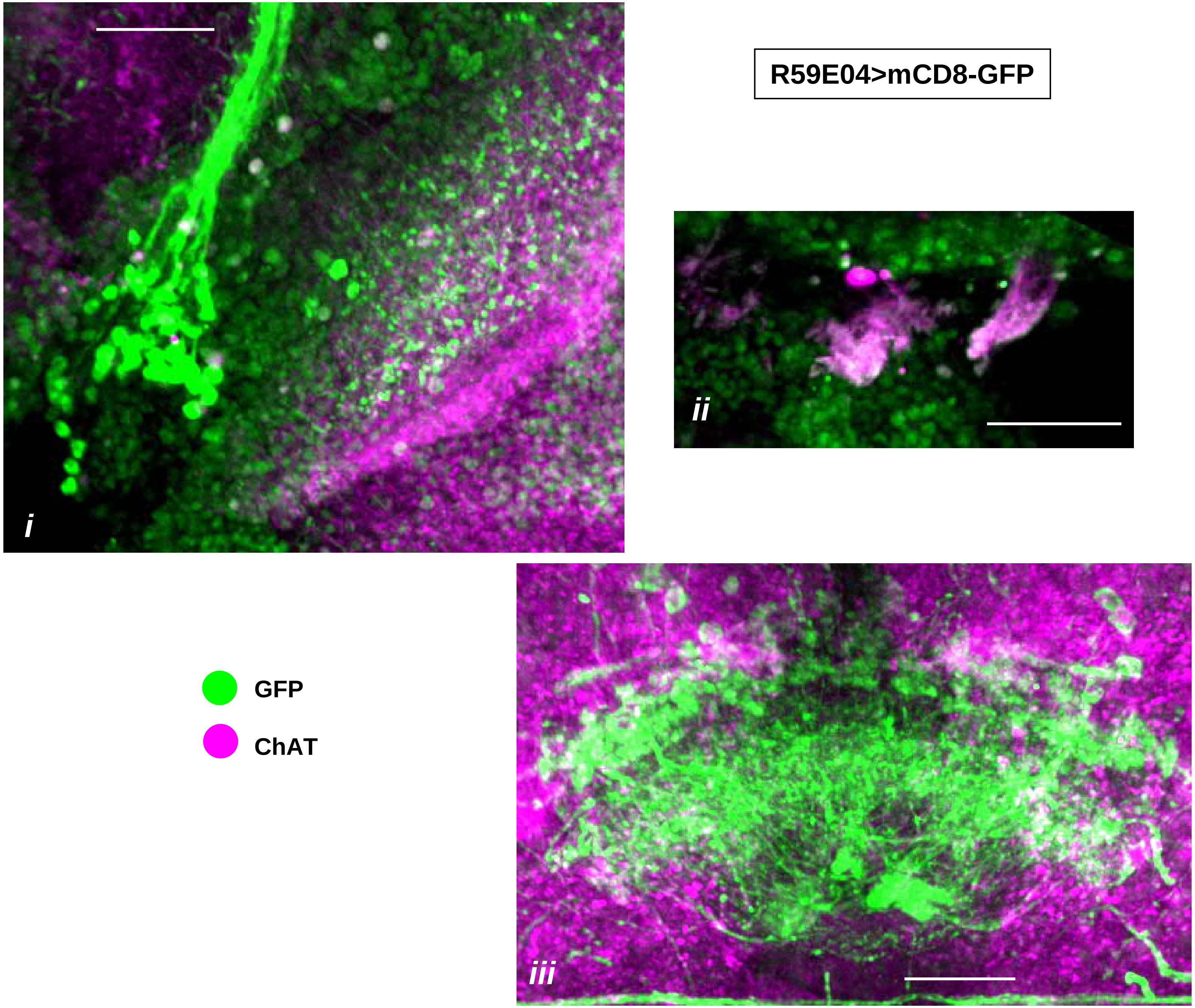

Yalgin et al, Figure S19

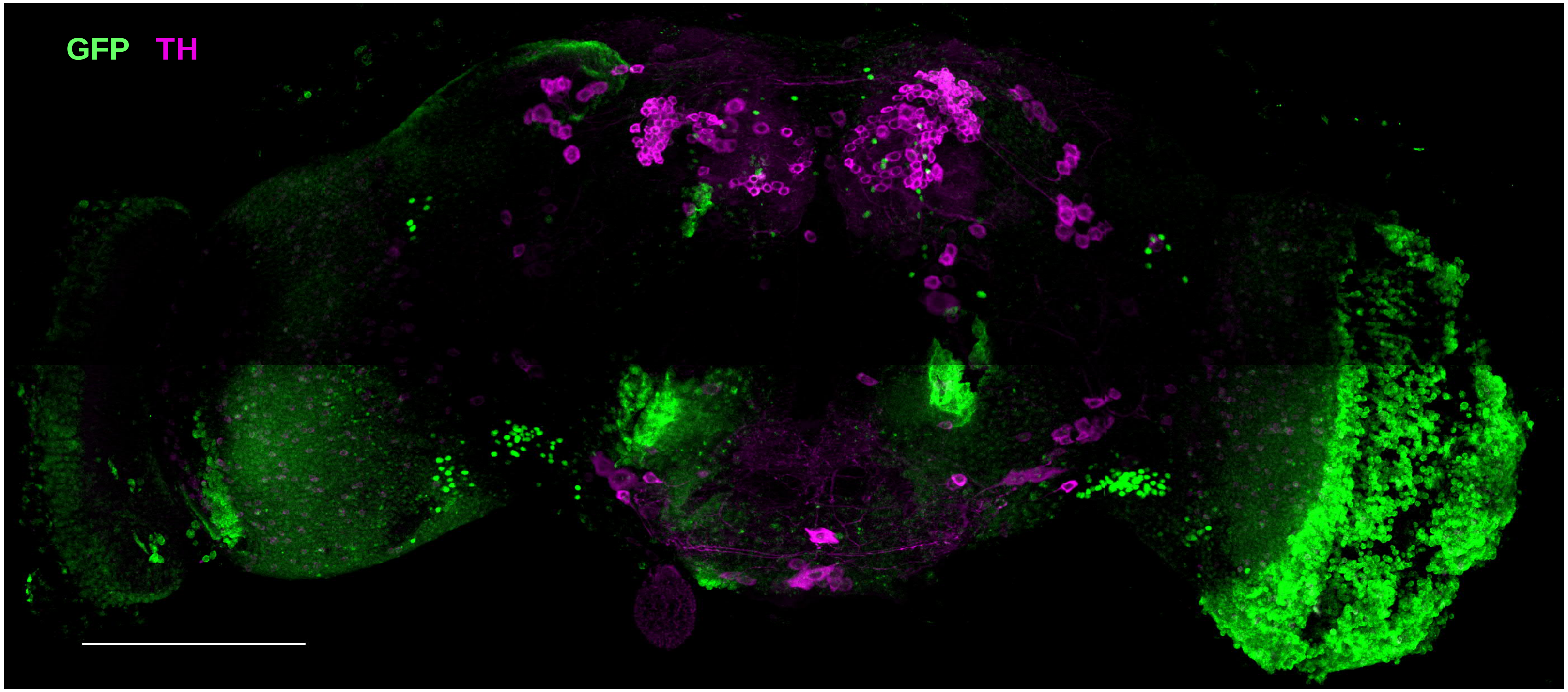

R59E04>nGFP

Yalgin et al, Figure S20

A

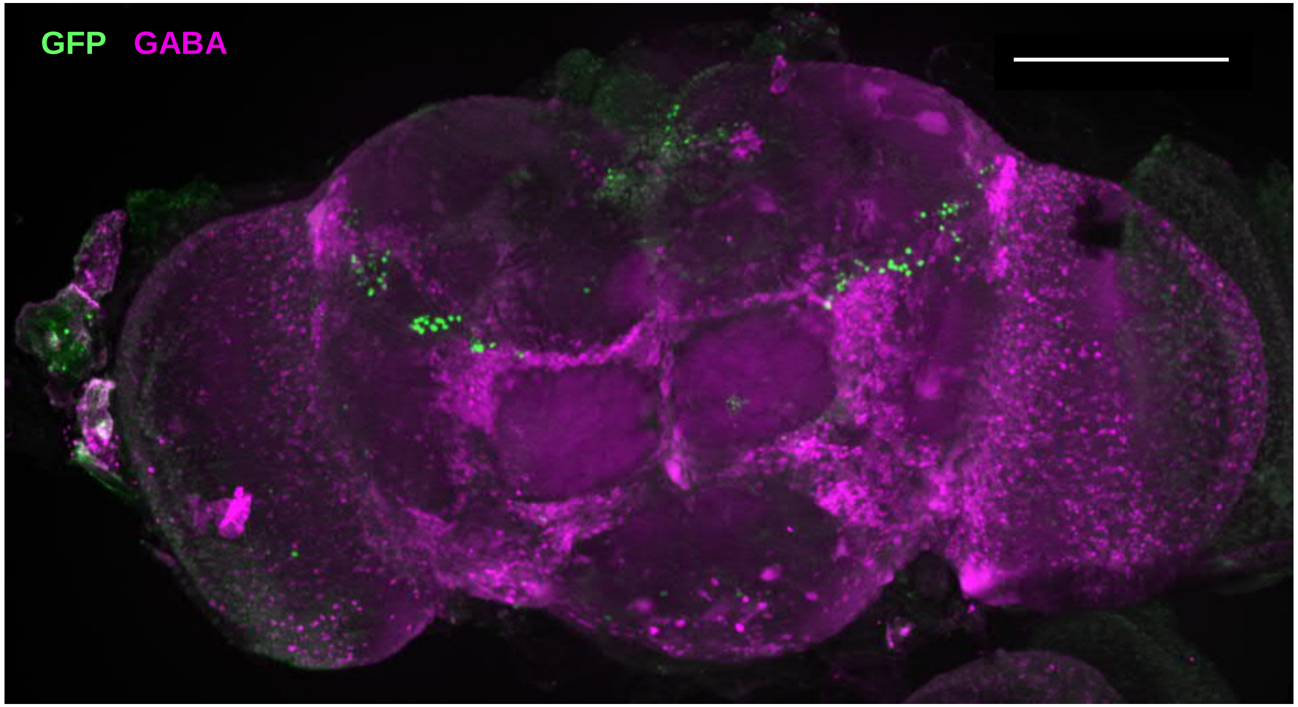

B

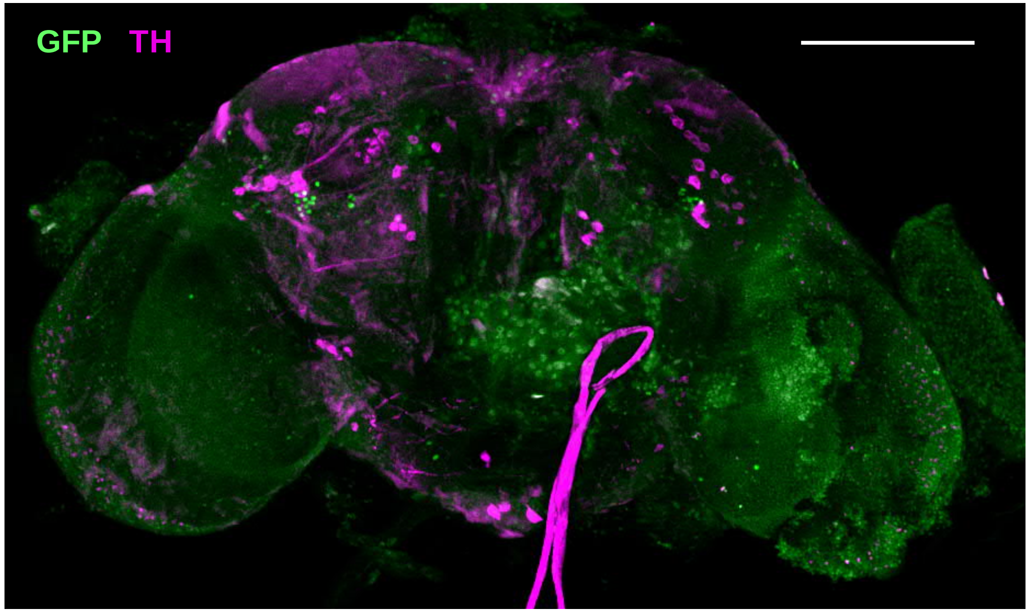

R51C09>nGFP

Yalgin et al, Figure S21

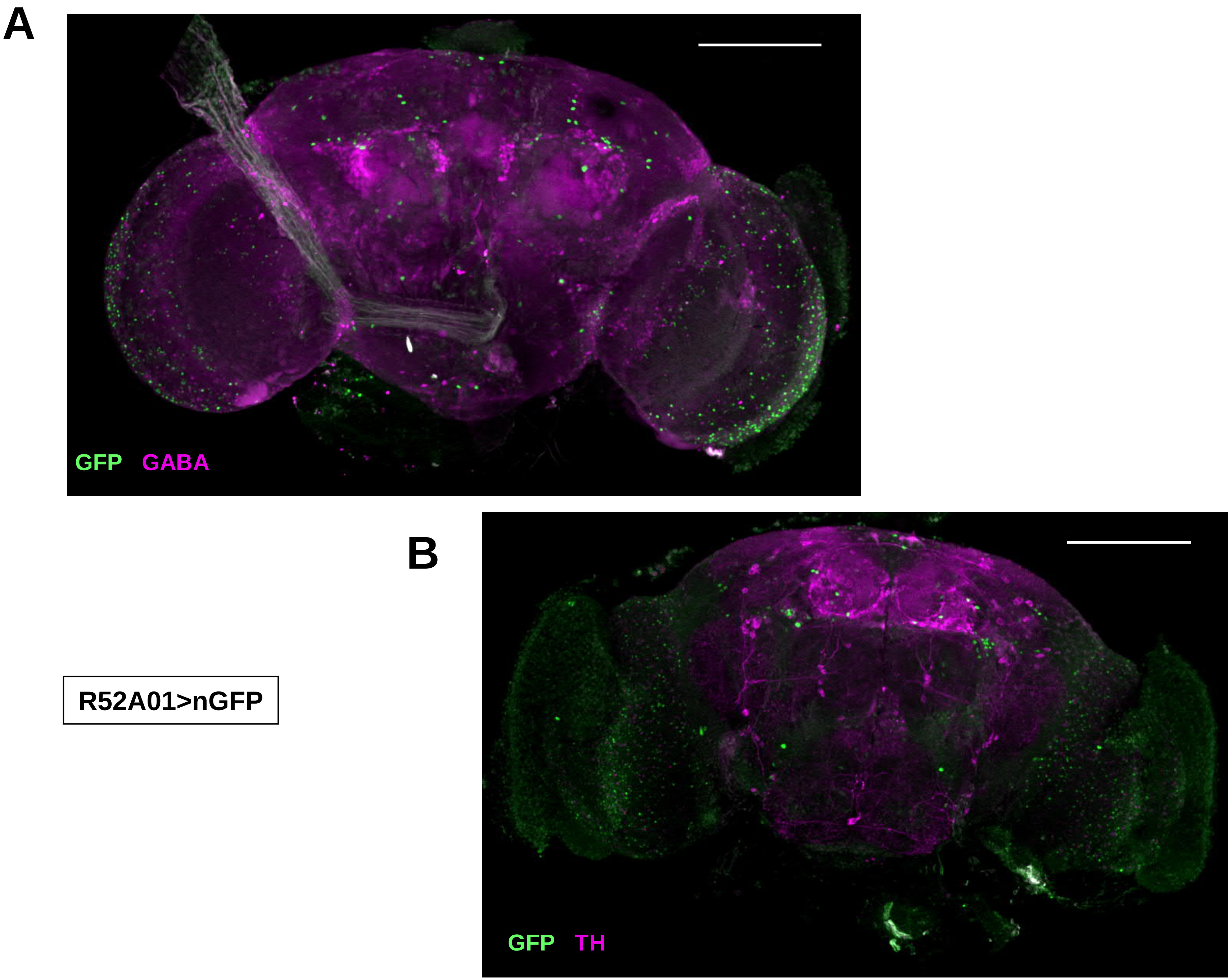

Yalgin et al, Figure S22

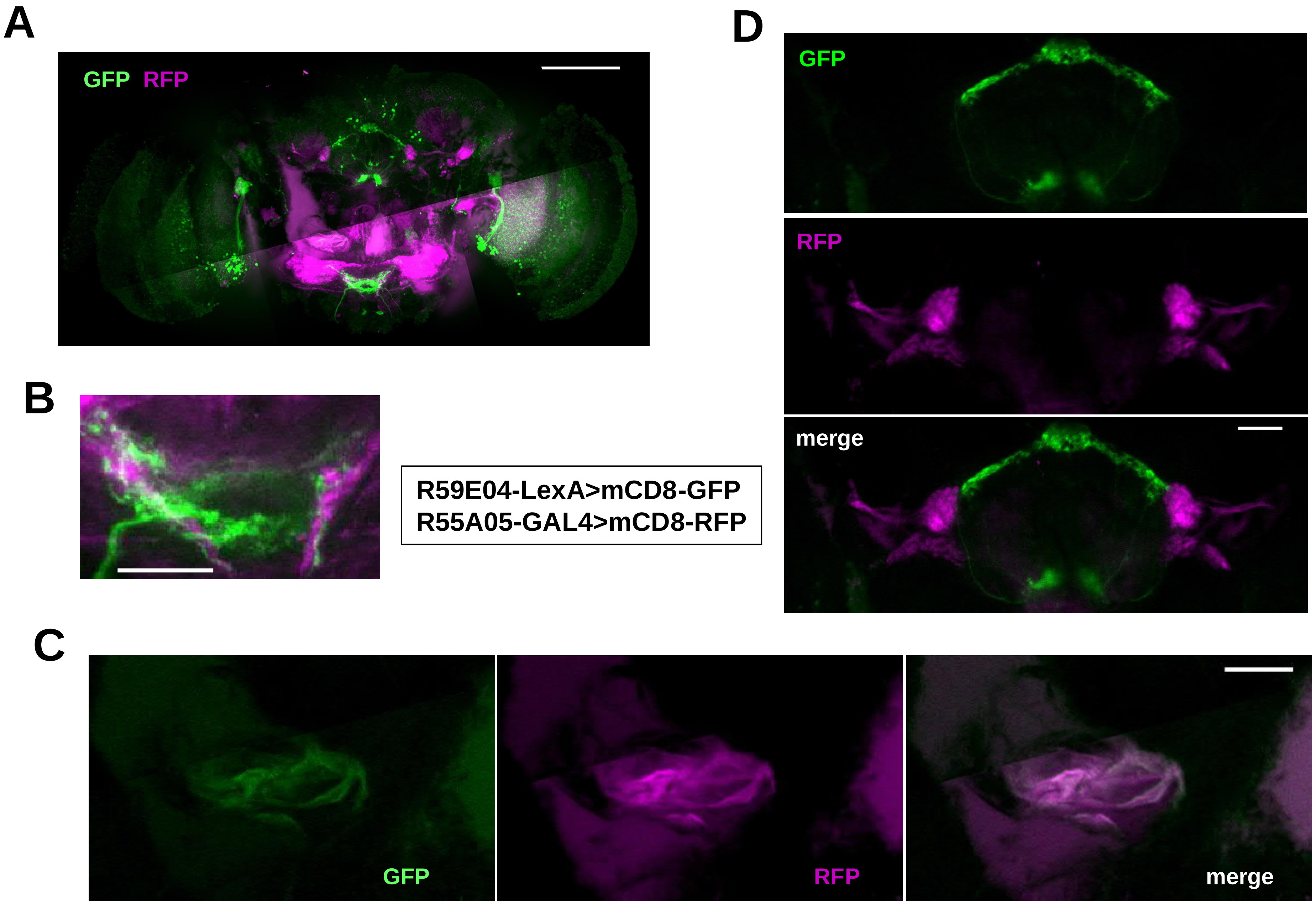

Yalgin et al, Figure S23

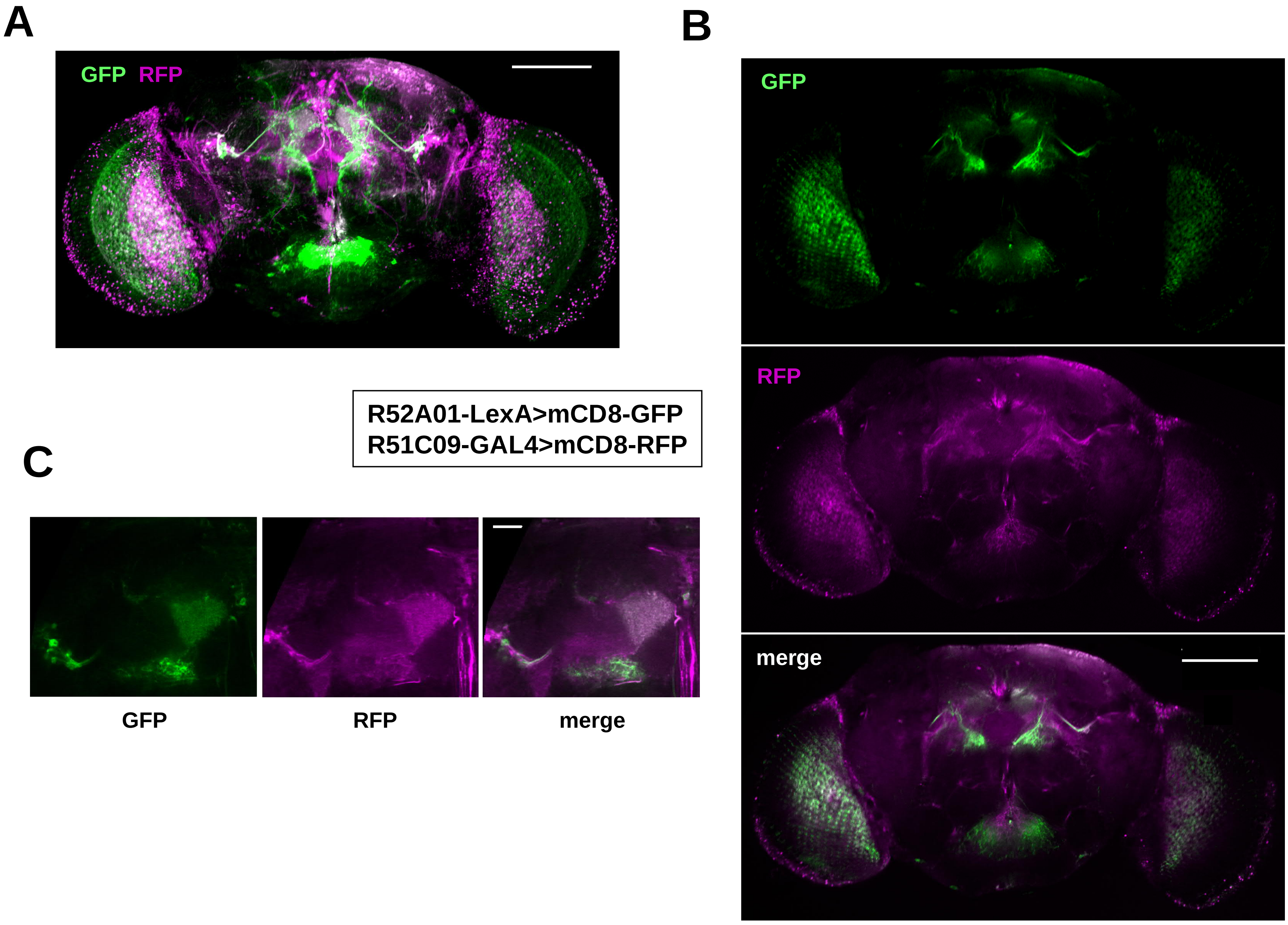

Yalgin et al, Figure S24

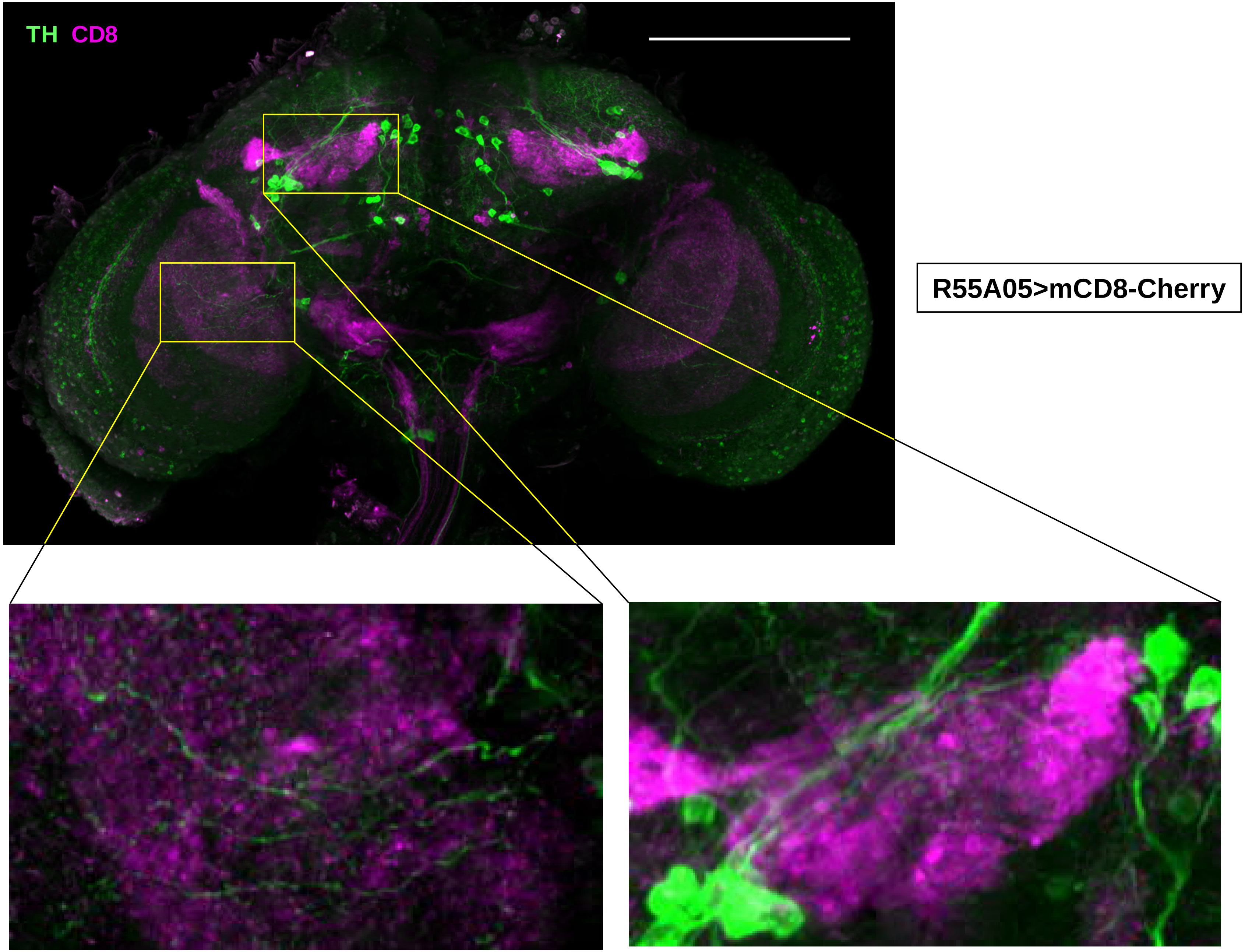

Yalgin et al, Figure S25

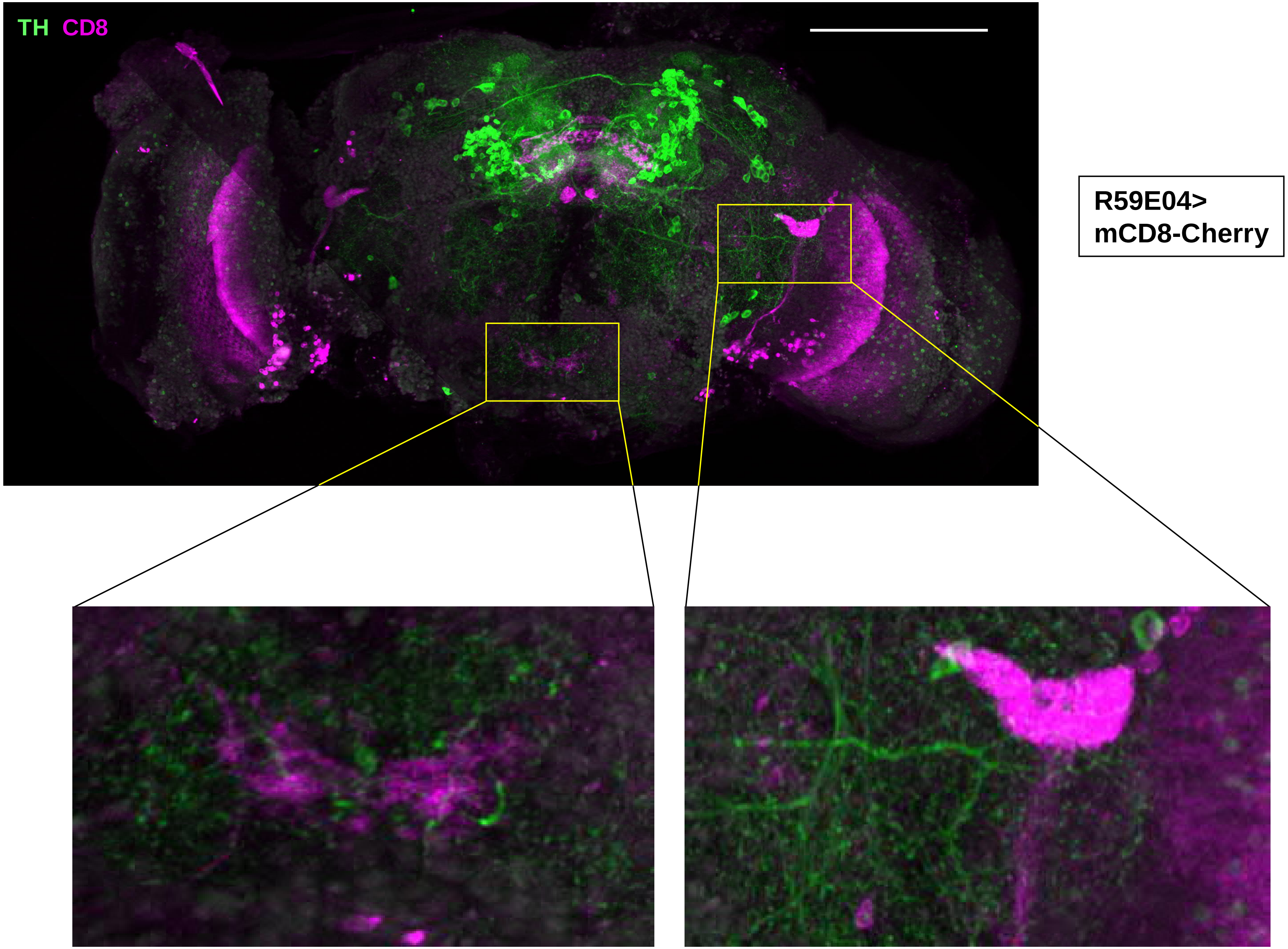

Yalgin et al, Figure S26

panels *v* and *iii* of Figure 7

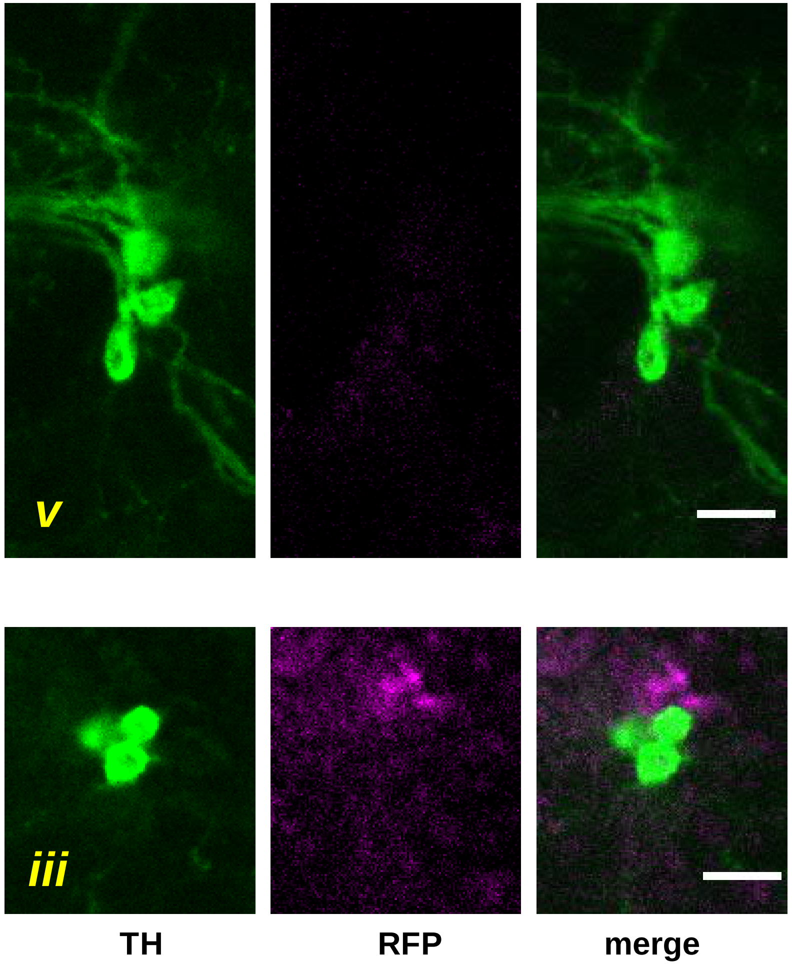

R55A05>trans-Tango

Yalgin et al, Figure S27

panels *ii*, *x* and *vii* of Figure 7

R55A05>trans-Tango

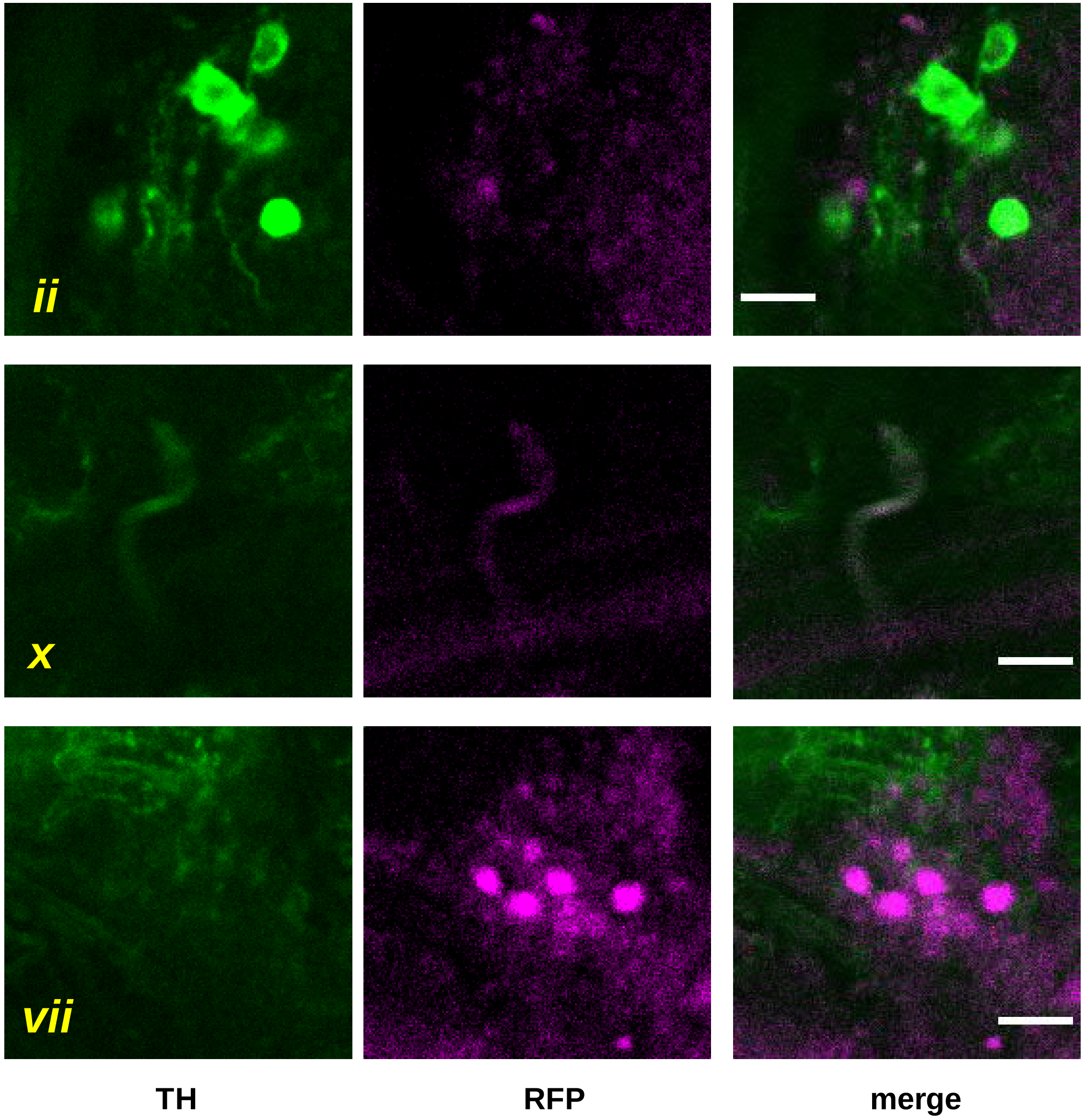

Yalgin et al, Figure S28

panel *xii* of Figure 7

R55A05>trans-Tango

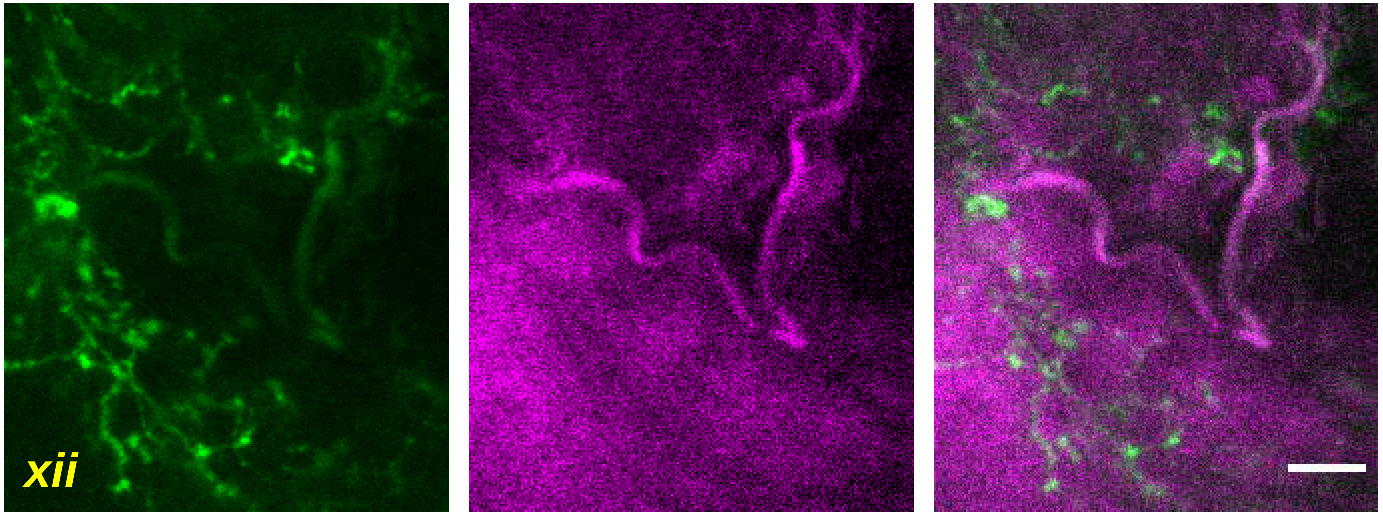

TH RFP merge

panels *v* and *iii* of Figure 7

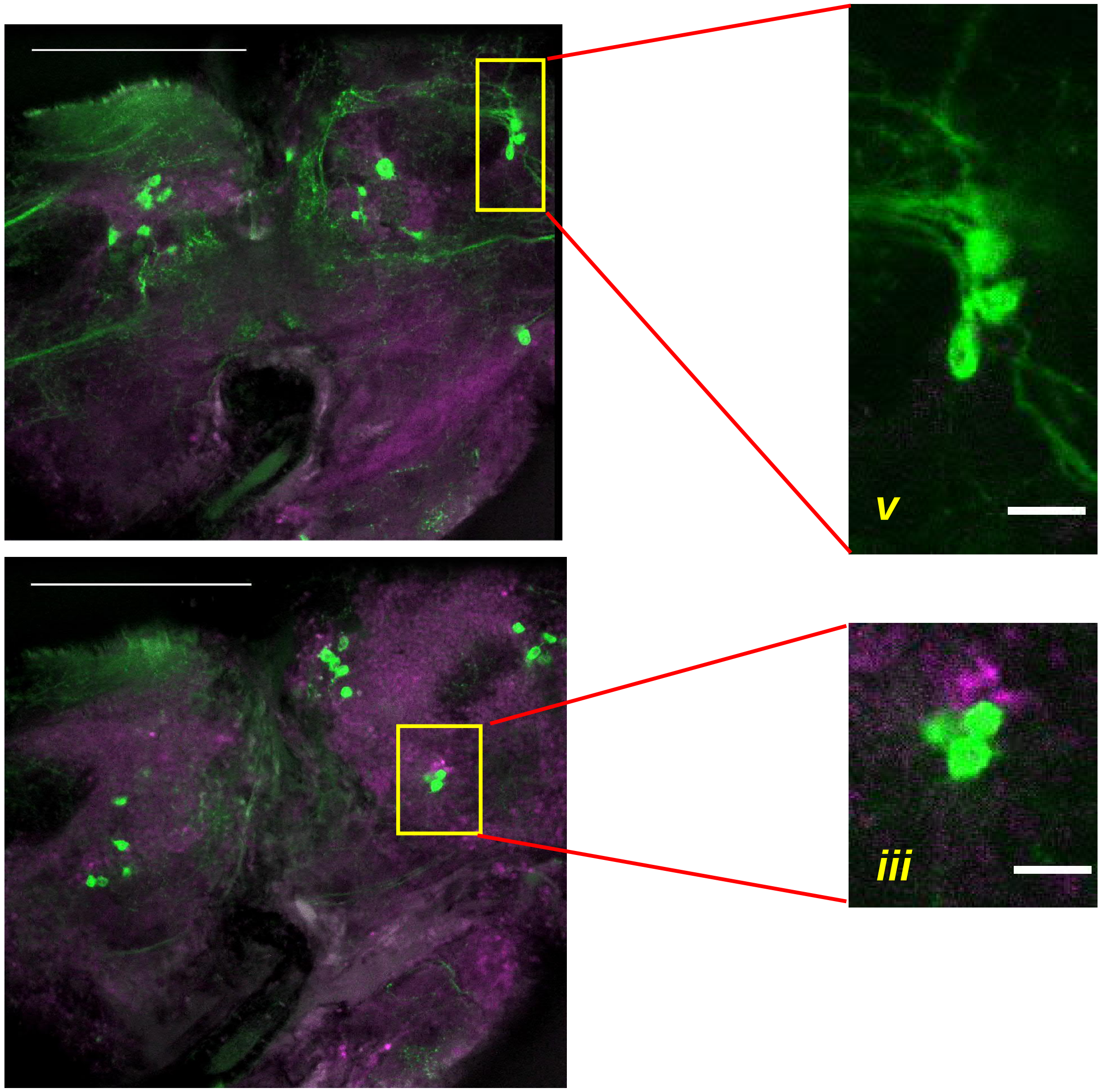

R55A05>trans-Tango

panels *i*, *ii* and *iv* of Figure 7

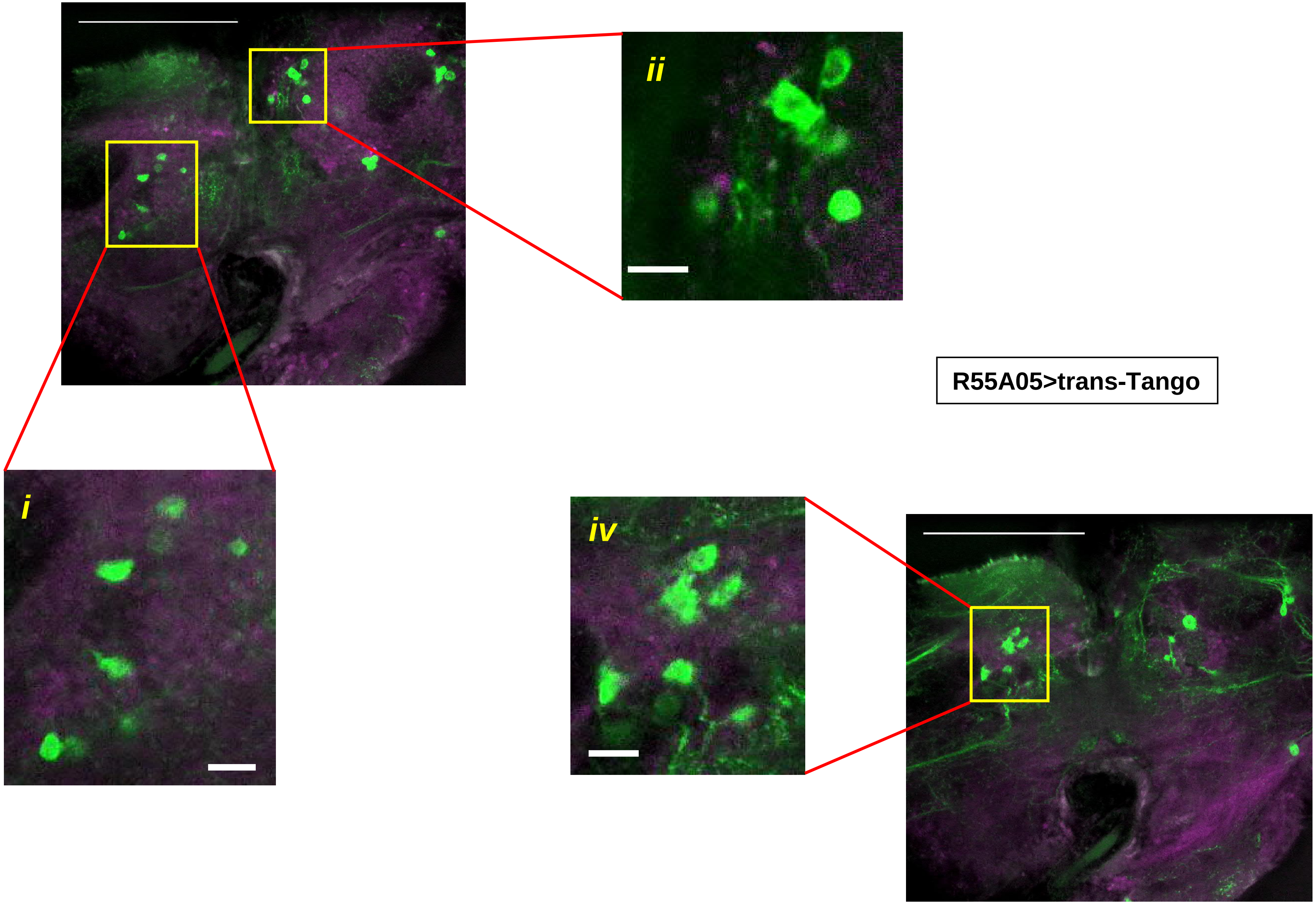

Yalgin et al, Figure S31

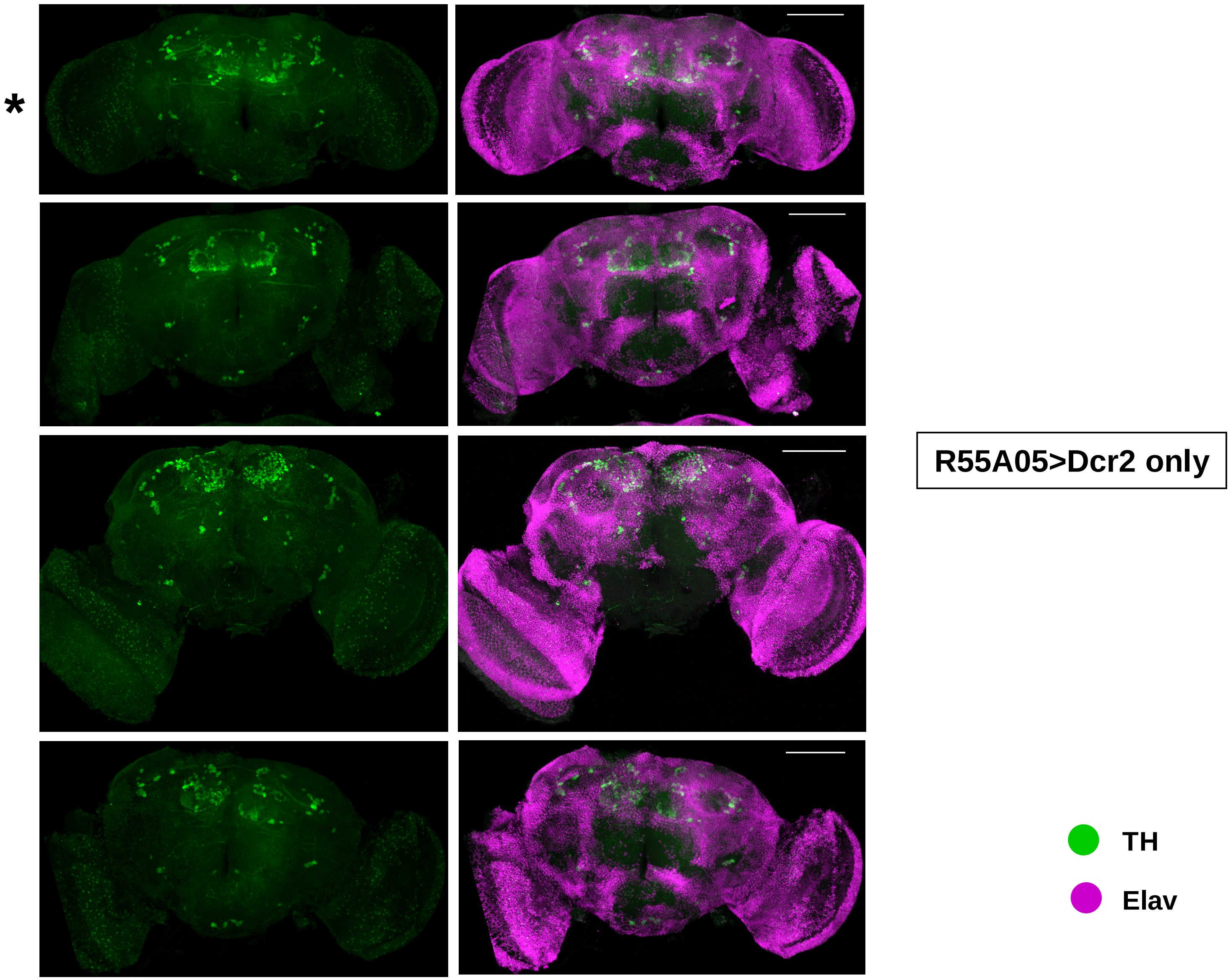

Yalgin et al, Figure S32

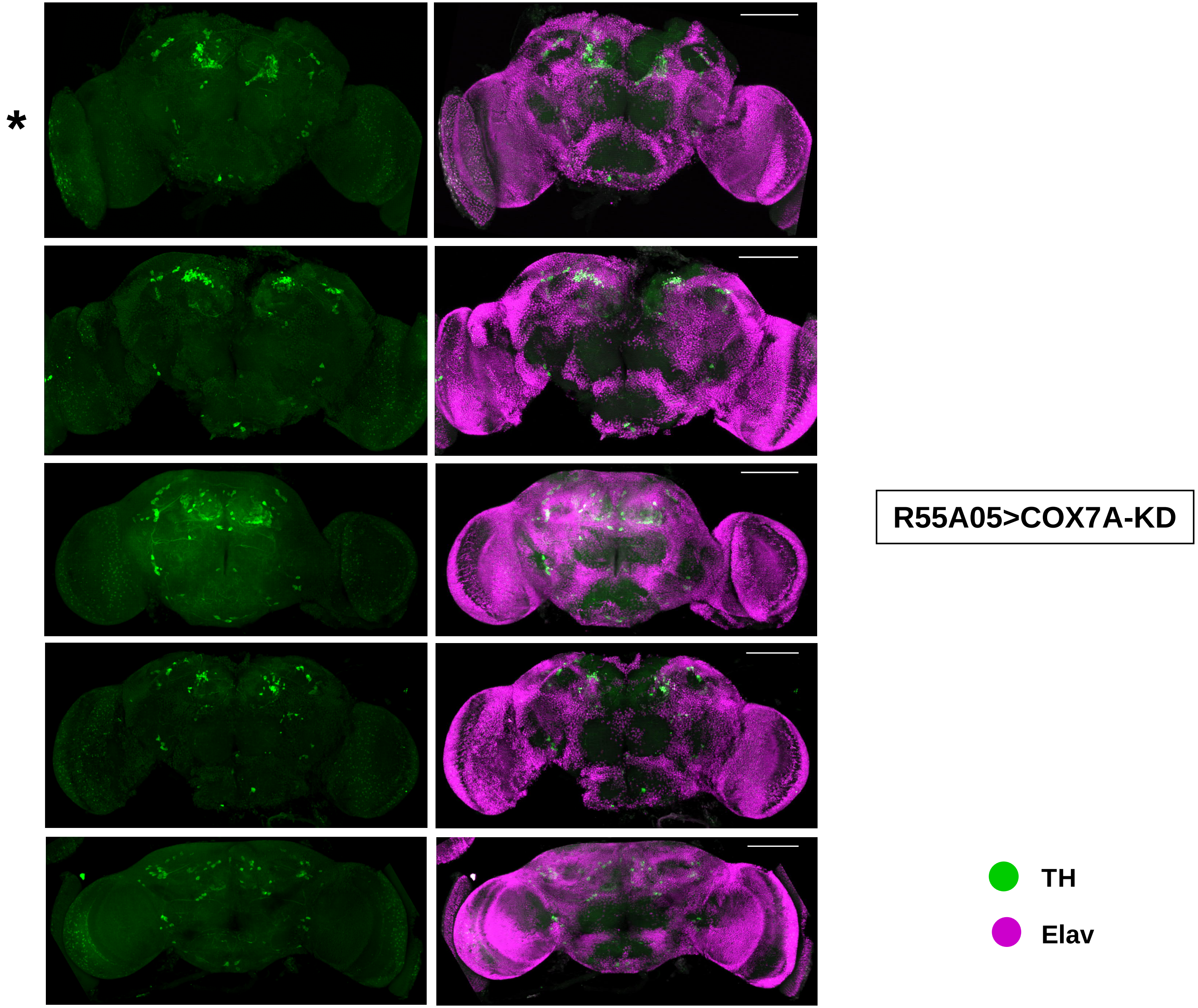

Yalgin et al, Figure S33

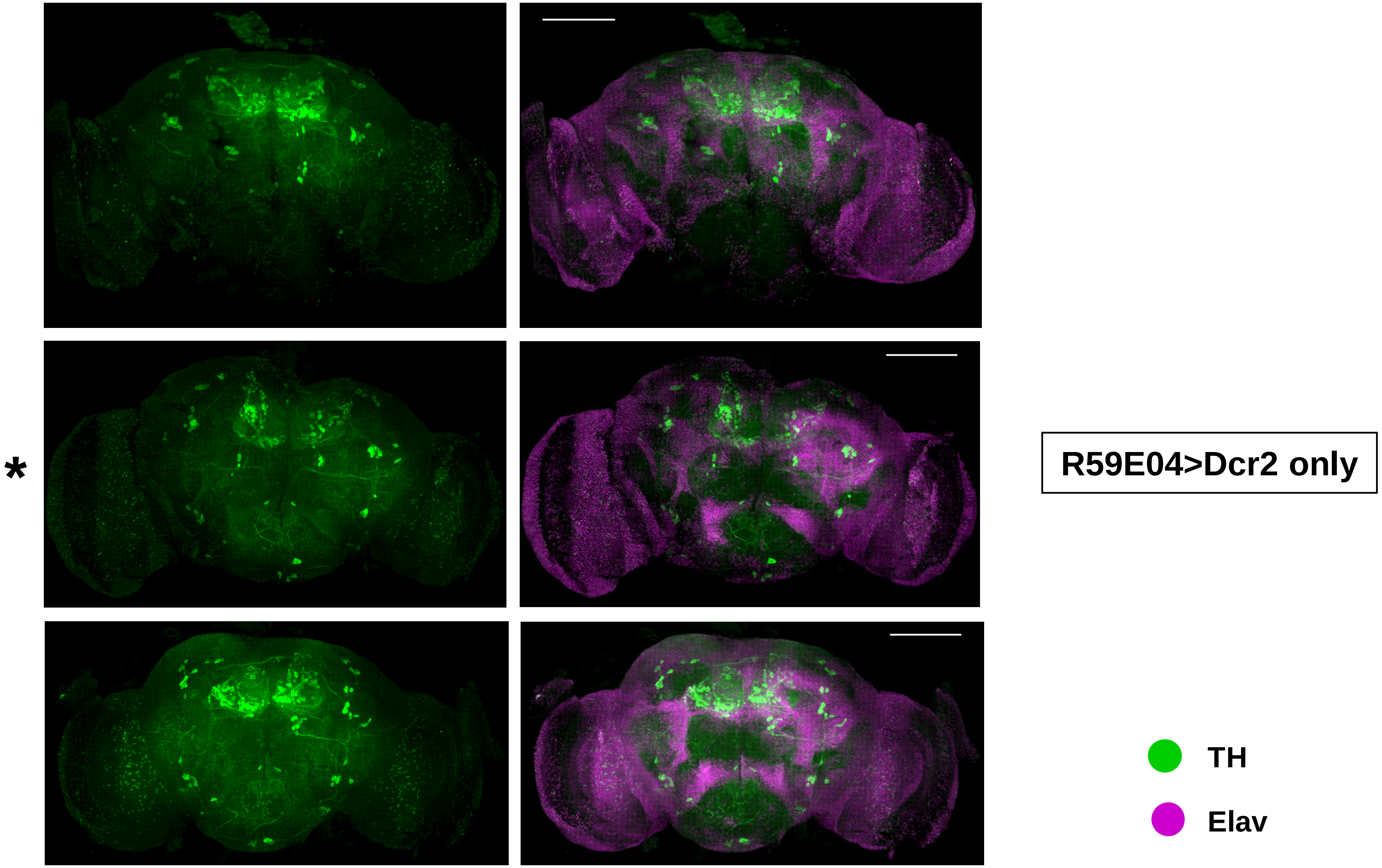

Yalgin et al, Figure S34

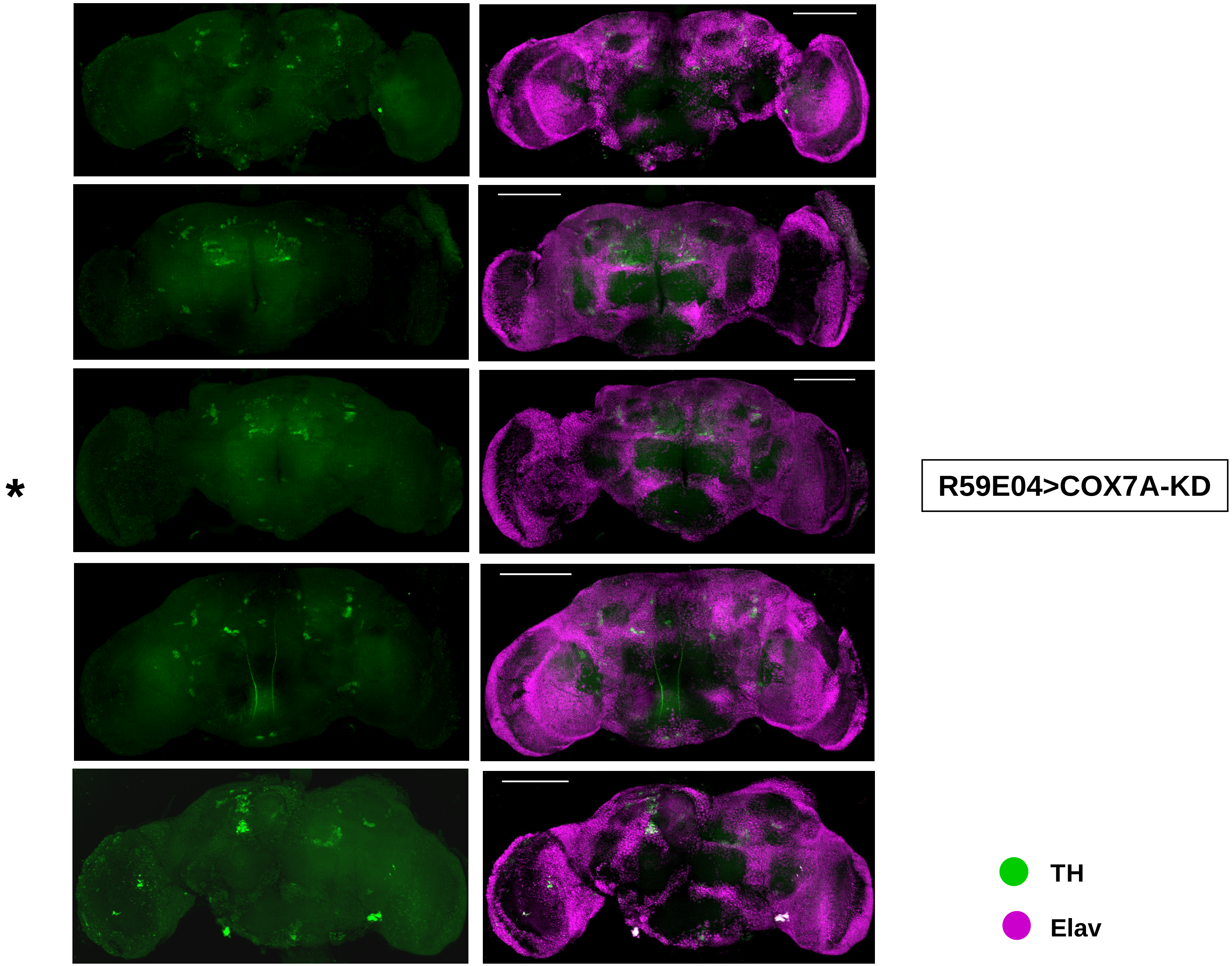

Yalgin et al, Figure S35

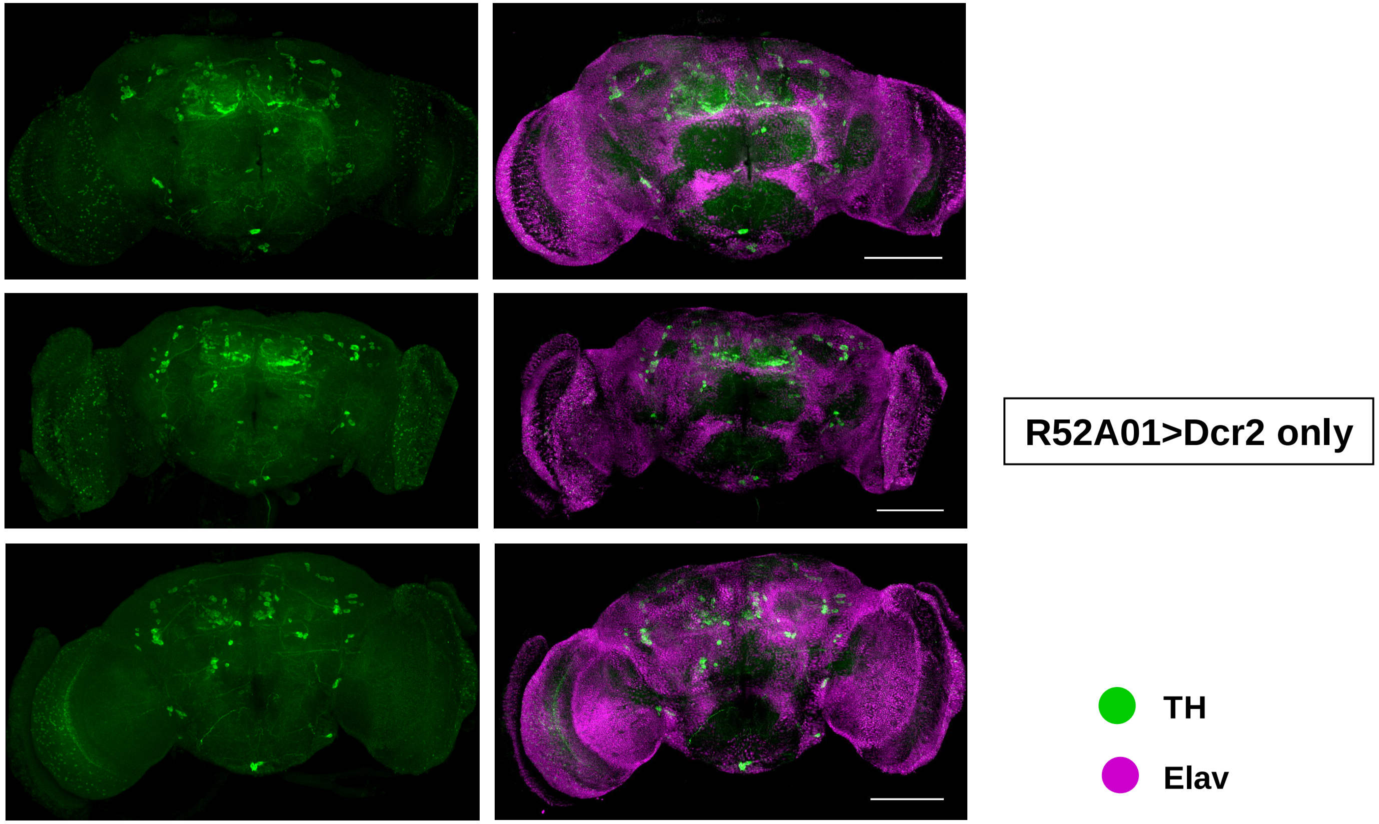

Yalgin et al, Figure S36

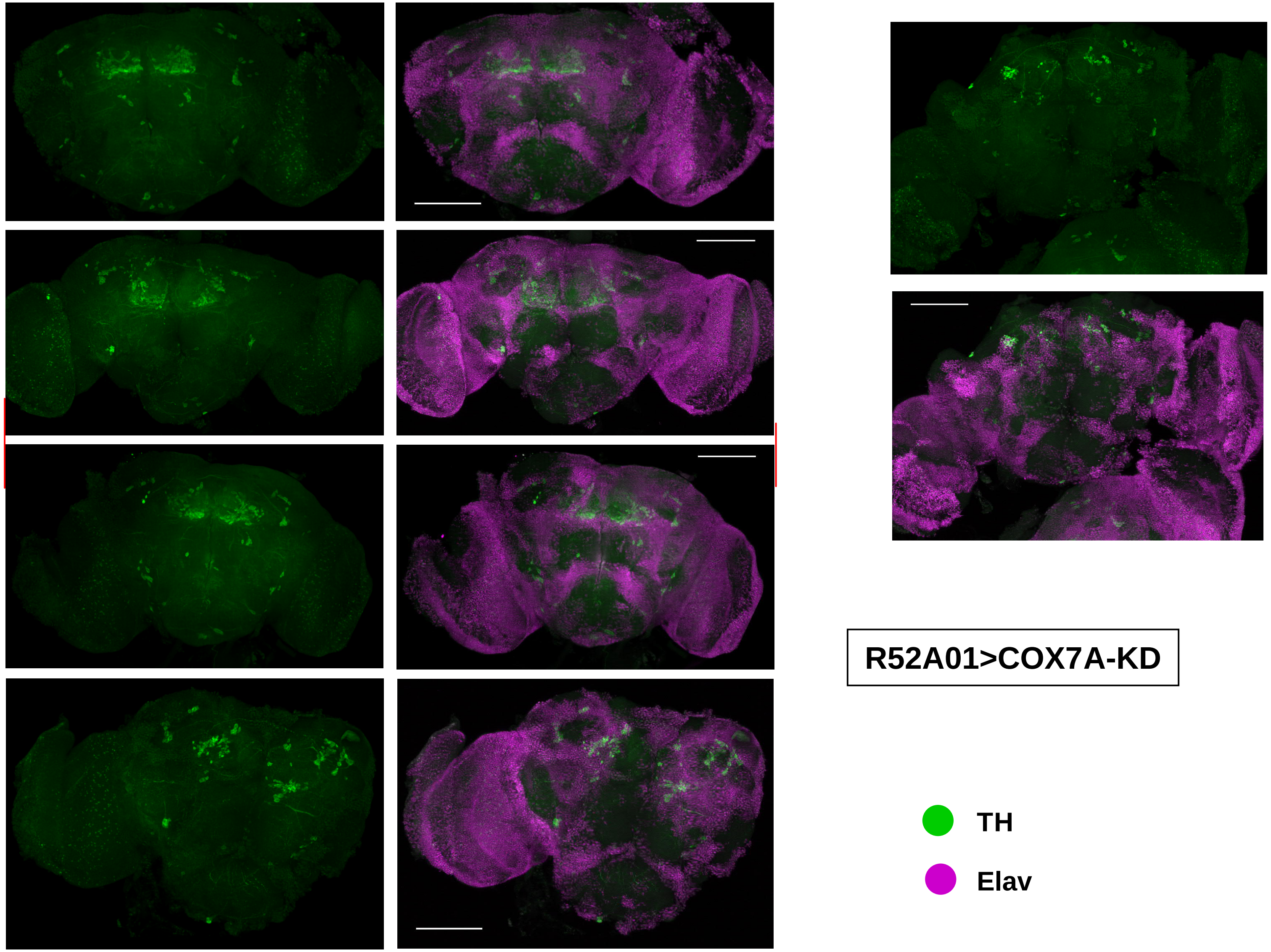

Yalgin et al, Figure S37

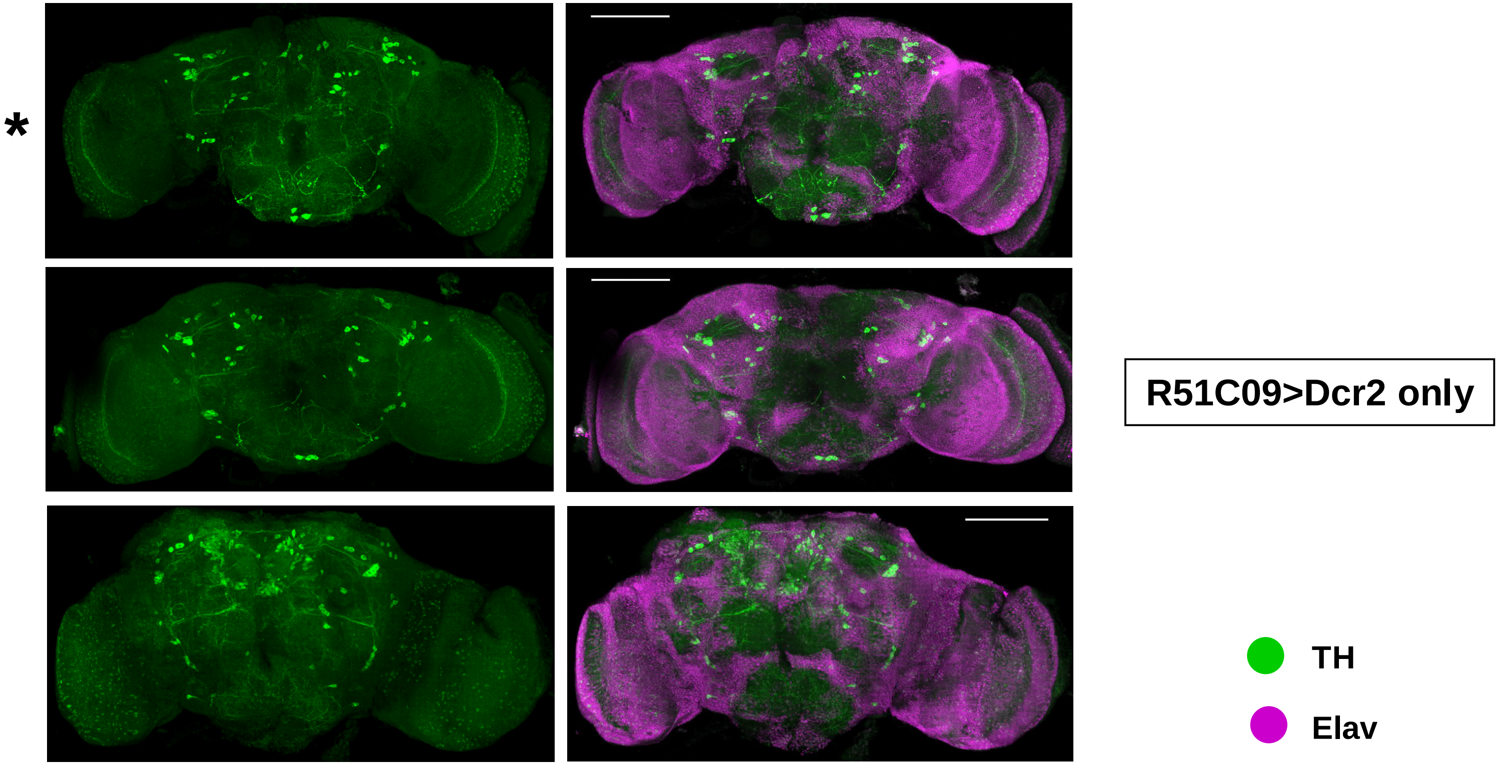

Yalgin et al, Figure S38

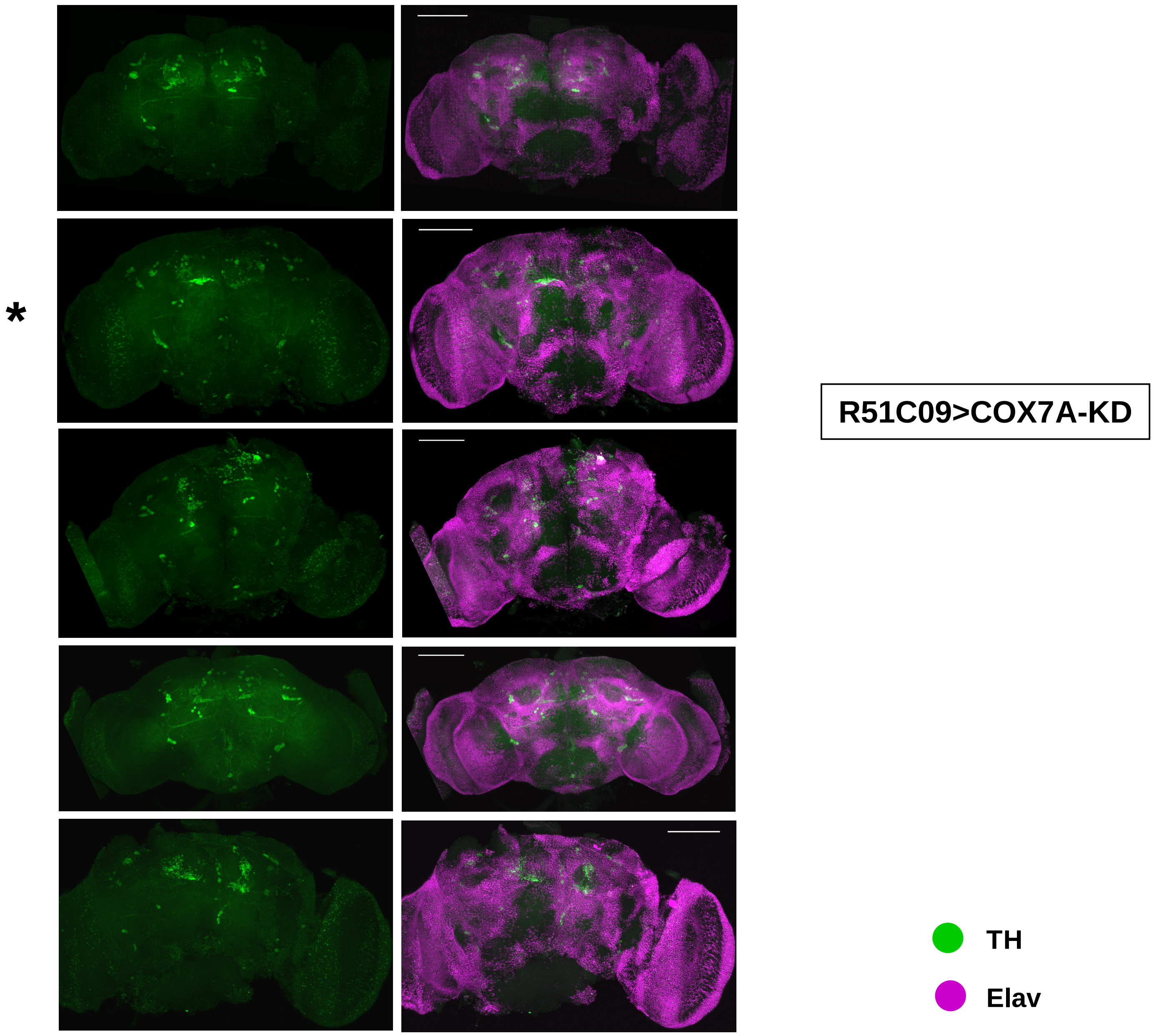

Yalgin et al, Figure S39
